# Farnesyltransferase Enables Modular Assembly of Dual‐Functional Nanobody Conjugates for in Vivo Imaging of Inflammation

**DOI:** 10.1002/anie.5009703

**Published:** 2026-08-24

**Authors:** Sneha Venkatachalapathy, Yoon Ho Lee, Ali Salehi Farid, Heydar Moravej, Sina Djafari Rouhani, Jennifer E. Rowley, Mohammad Rashidian, Mark D. Distefano

**Affiliations:** ^1^ Department of Chemistry University of Minnesota Minneapolis Minnesota USA; ^2^ Department of Cancer Immunology and Virology Dana‐Farber Cancer Institute Boston Massachusetts USA; ^3^ Parker Institute for Cancer Immunotherapy San Francisco California USA; ^4^ Department of Radiology, Brigham and Women's Hospital Harvard Medical School Boston Massachusetts USA

**Keywords:** click chemistry, farnesyltransferase, nanobodies, PET imaging, selective protein modification

## Abstract

Non‐invasive imaging of inflammation is critical for disease diagnosis, monitoring, and therapeutic evaluation. Nanobodies, due to their many unique properties, are emerging as powerful imaging agents; however, conventional non‐specific conjugation strategies often yield heterogeneous products that limit in vivo performance. Here, we report a chemoenzymatic strategy that enables precise and modular assembly of nanobody conjugates using farnesyltransferase (FTase)‐mediated site‐specific modification. We developed a trifunctional FTase substrate that introduces two orthogonal bioorthogonal handles, an aldehyde and an azide, onto nanobodies in a single enzymatic step. These handles enable independent and controlled conjugation of imaging payloads (fluorophores or radioisotopes) and pharmacokinetic modifiers (i.e. PEG), which enhance in vivo signal‐to‐noise ratios. Applying this strategy to nanobodies targeting CD45 and CD11b, markers broadly expressed on all immune cells and myeloid subsets, respectively, we generated well‐defined, dually modified constructs that enabled high‐contrast in vivo visualization of immune organs, including the spleen and lymph nodes. In a mouse model of localized inflammation, these probes robustly delineated inflamed tissue with high sensitivity and specificity. Together, this FTase‐enabled dual‐labeling approach provides a versatile and generalizable platform for the rapid generation of precisely defined nanobody‐based imaging agents, advancing non‐invasive detection and monitoring of inflammation across diverse disease contexts.

## Graphical abstract

A modular farnesyltransferase‐based chemoenzymatic platform enables fast, site‐specific labeling of nanobodies for PET imaging of inflammation. CD11b‐targeted PET agents selectively image myeloid‐driven inflammation, while CD45‐targeted PET agents provide sensitive whole‐body immune mapping. Together they provide the possibility of complementary, quantitative immuno‐PET for monitoring inflammatory burden potentially beyond ^18^F‐fluorodeoxyglucose imaging.

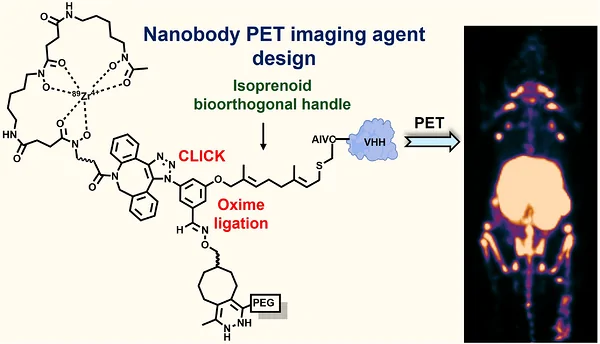

## 1 Introduction

Pathologic inflammation underlies a wide spectrum of diseases, including cancer, diabetes, neurodegenerative disorders, cardiovascular disease, non‐alcoholic fatty liver disease, and autoimmune conditions such as vasculitis, arthritis, and colitis [1, 2, 3]. Although these afflictions present with distinct clinical symptoms, they share a common underlying mechanism: systemic inflammation. This persistent response often creates a vicious cycle where disease symptoms exacerbate inflammation, which in turn drives further disease progression. Early detection and monitoring of inflammatory markers are therefore critical to interrupt this cycle and prevent worsening outcomes. Current non‐invasive clinical evaluations rely on physical examination [4], blood tests [5], and imaging techniques such as x‐rays [6], magnetic resonance imaging (MRI) [7], computed tomography (CT) [8], and ultrasound [9, 10]. However, these approaches often detect disease only at advanced stages or lack specificity. In contrast, new hybrid modalities such as positron emission tomography/computed tomography (PET/CT) use specific probes to integrate functional data from PET with anatomical detail from CT, offering a non‐invasive, whole‐body approach for early detection and monitoring of inflammation [10, 11, 12, 13].

Although widely used for inflammation imaging, ^18^F‐FDG lacks immune‐cell specificity and often shows limited sensitivity to inflammatory cell burden because it reports nonspecific glucose uptake [14, 15, 16]. An ideal imaging probe should be target‐specific, biologically inert, serum‐stable, minimally immunogenic, and capable of rapid biodistribution with low background retention [17]. While full‐length antibodies were first introduced for molecular imaging over four decades ago, beginning with the ^131^I‐labeled IgG targeting carcinoembryonic antigen in 1978 [18], their clinical utility has been limited by slow pharmacokinetics [19]. Prolonged circulation times, often persisting for days to weeks, result in extended patient radiation exposure and create significant logistical challenges for clinical imaging. In contrast, peptides exhibit strong target‐binding properties and rapid biodistribution but suffer from poor serum stability due to enzymatic degradation. Nevertheless, several peptide‐based PET probes have been developed, and their continued optimization remains an active area of research [20, 21]. To overcome these limitations of both full‐length antibodies and peptides, smaller engineered antibody fragments including scFvs, minibodies, and single‐domain antibodies (nanobodies/Variable Heavy domain of Heavy chain (VHH) have been developed. VHHs (∼14 kDa), derived from camelid heavy‐chain‐only antibodies, offer high specificity and affinity, exceptional stability, and rapid biodistribution. Their small size and the orientation of their C terminus, positioned away from the antigen‐binding site, make them ideal for site‐selective functionalization, enabling their widespread use in molecular imaging and targeted diagnostics [22, 23, 24]. Owing to their small size, nanobodies undergo rapid glomerular filtration (t_1/2_ of ∼0.5 h) followed by non‐specific reabsorption in the proximal renal tubules, resulting in high kidney accumulation [25, 26].

Therefore, the development of effective nanobody‐based PET probes requires careful consideration of two key design parameters. First, PEGylation provides an effective strategy to reduce renal retention and enhance in vivo signal‐to‐noise ratios [22, 23]. Second, the methodology used to attach PEG moieties and chelators for PET isotope installation must be mild and site‐selective. Precise control over the number and site of payload attachment enables the generation of well‐defined, efficient, and reproducible imaging agents while preserving nanobody binding capacity, a critical requirement for in vivo imaging. Enzymatic protein modification offers distinct advantages, including mild aqueous conditions that preserve biomolecular integrity, and supports site‐specific labeling through fewer steps when combined with high yielding bioorthogonal chemistry [27, 28]. This modular approach can significantly improve the stability, pharmacokinetics, and functional performance of protein‐based imaging agents compared to traditional, heterogeneous chemical conjugation methods.

Several site‐selective conjugation strategies have been employed to generate protein‐based reagents for molecular imaging [29, 30]. Common enzymatic approaches include the use of microbial transglutaminase (mTG) [31, 32], lipoic acid ligase [33, 34], galactosyltransferase [35, 36], glycine formyl generating enzyme [37, 38], and sortase A (SrtA) [39, 40]. In addition, self‐labeling protein domain‐based strategies have emerged as useful alternatives for site‐selective probe installation in molecular imaging, including the SpyTag/SpyCatcher protein ligase system [41] and recently developed self‐labeling single‐domain antibody platforms [42]. These methods enable modular and covalent attachment of imaging payloads, further broadening the toolbox for site‐specific protein labeling. Recent studies have demonstrated the successful use of SrtA‐mediated synthesis of monomeric and dimeric VHH PET probes for imaging inflammation in various murine models [22, 23, 40]. However, its low binding affinity for the LPXTG sequence necessitates a high molar excess of peptide nucleophiles and SrtA or extended reaction times making this approach costly for large‐scale applications [43]. Extensive research from our group has established farnesyltransferase (FTase) mediated modification as a robust, site‐selective strategy for C‐terminal protein labeling, enabling the installation of bioorthogonal handles for conjugation of fluorophores [44], cytotoxins [45], PEG [46], DNA [47], or for assembling multimeric protein complexes [48, 49, 50, 51]. FTase specifically recognizes a C‐terminal CaaX tetrapeptide motif, where C is cysteine, “a” represents aliphatic residues, and X is any amino acid, and catalyzes the transfer of a farnesyl group from farnesyl diphosphate (FPP) to the cysteine resulting in stable thioether bond formation (Figure 1a). Since FTase requires only a C‐terminal CaaX motif for efficient modification, it can be used to modify virtually any protein by fusion of the tetrapeptide recognition sequence to the target protein of interest. Due to its inherent substrate flexibility, numerous isoprenoid analogues incorporating biorthogonal functionality have been used for a broad range of applications ranging from metabolic incorporation for proteomic analysis [52, 53, 54, 55, 56] to selective protein modification [44, 45, 49, 57, 58, 59, 60]. In our recent study using ^89^Zr‐based PET imaging demonstrated that a VHH‐based PET probe targeting the pan‐leukocyte marker CD45 provides a favorable balance of sensitivity and selectivity for inflammation detection. Unlike tracers specific to individual immune subsets, CD45‐PET enables comprehensive imaging of immune cell infiltration, thereby enhancing sensitivity to inflammatory processes. In direct comparisons, CD45‐PET outperformed both ^18^F‐FDG and CD11b‐PET in models of lung inflammation and inflammatory bowel disease, exhibiting higher standardized uptake values (SUVs) and a stronger correlation with disease severity [61].

**FIGURE 1:**
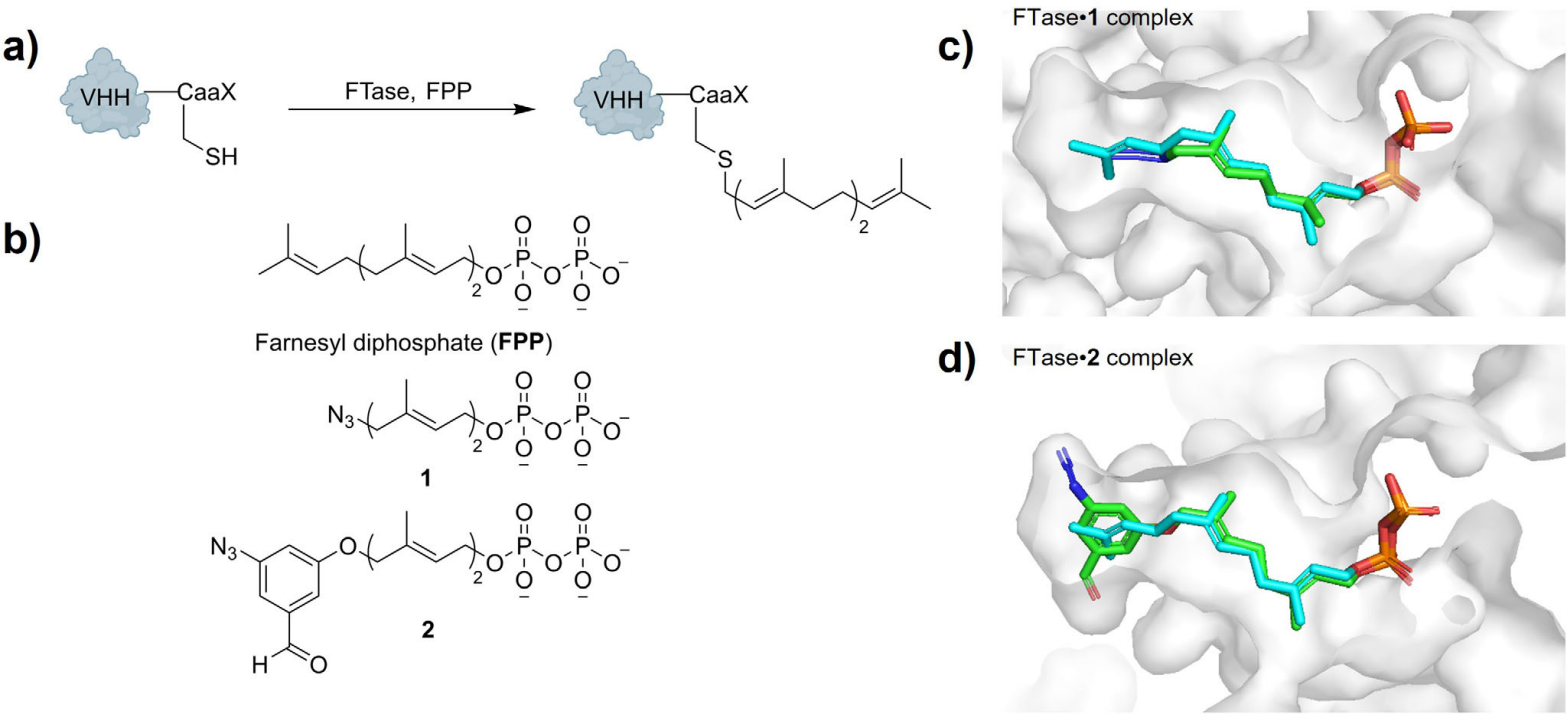
Reaction catalyzed by farnesyltransferase and substrates and analogs used in this study. (a) Schematic representation of farnesylation reaction with a protein containing a CaaX box at its C terminus. (b) Structures of FPP and alternative substrates **1** and **2**. (c) Docking of **1** with FTase. (d) Docking of **2** with FTase, superimposed with the natural substrate (FPP) bound to FTase (from x‐ray crystallographic data; PDB ID: 1JCR). FPP is shown in cyan, synthetic substrates are shown in green (C), blue (N), red (O), and orange (P). The protein structure is shown in white.

In this study, we investigated an FTase‐mediated strategy for the site‐specific preparation of PEGylated, ^89^Zr‐labeled nanobodies and evaluated their performance as PET imaging probes in vivo. Using nanobodies targeting CD45, a pan‐leukocyte marker, and CD11b, a myeloid‐specific marker, we assessed the ability of these probes to visualize immune organs and detect inflammation across multiple disease contexts. To enable nanobody‐based PET imaging, we first developed a site‐selective labeling approach using a bifunctional FTase substrate (FPP analog **1**; Figure 1b) bearing an azide handle for strain‐promoted azide‐alkyne cycloaddition (SPAAC) with a deferoxamine (DFO) chelator, allowing subsequent installation of the ^89^Zr radionuclide. We then advanced this platform by developing a trifunctional FTase substrate (FPP analog **2**; Figure 1b) incorporating both azide and aldehyde functionalities, enabling orthogonal dual conjugation via SPAAC and oxime ligation to install both the DFO chelator and PEG moieties (5 kDa or 10 kDa) in a controlled manner. Both strategies yielded well‐defined, high‐quality imaging probes; however, the dual‐functional approach enabled simultaneous PEGylation and radiolabeling, resulting in improved pharmacokinetics and enhanced in vivo image quality.

An important consideration in the use of prenylated FTase substrates is the potential effect of the appended hydrophobic group on protein solubility, serum interactions, and in vivo behavior. To place this in context, the physicochemical properties of related isoprenoid‐based substrates developed in our laboratory were analyzed in recent work (Table S1) [62], where calculated log P (c Log P) values were reported. The geraniol‐derived precursor used to access the FTase substrates in this study has a c Log P of 2.97, whereas a comparable straight‐chain alkyl alcohol has a c Log P of 6.64, corresponding to an approximately 4.7 × 10^3^‐fold increase in hydrophobicity. Notably, related C10 isoprenoid analogs of similar length developed in our laboratory span a more moderate c Log P range of 2.3–4.1, supporting the view that branched isoprenoid scaffolds generally have a less hydrophobic profile than analogous straight‐chain alkyl groups. In the present study, prenylation did not compromise nanobody stability, as the constructs remained soluble during purification, handling, and in vivo administration without observable aggregation; moreover, PEGylation is expected to further mitigate prenyl‐associated hydrophobicity by improving solubility and pharmacokinetics while reducing nonspecific interactions. It should also be noted that FTase‐catalyzed protein modification is limited to cysteine residues localized within CaaX motifs located at the C‐terminus. However, such a position is ideal for nanobody modification since it positions the site of conjugation remote from the antigen binding site. Moreover, if desired, circular permutation of the nanobody scaffold could be used to reposition the C‐terminus at a variety of other locations within the structure [63].

Overall, this approach provides a streamlined and modular workflow for site‐specific nanobody functionalization, enabling labeling in two steps using the bifunctional substrate **1** and in two or three steps using the trifunctional substrate **2** for PEGylated probes. This strategy enabled the efficient generation of well‐defined VHH‐based tracers optimized for high‐contrast ^89^Zr PET imaging of inflammation, achieving yields and conversion efficiencies of ≥90%. These probes enabled clear visualization of immune organs and robust detection of localized inflammation in a complete Freund's adjuvant (CFA)‐induced paw inflammation model. Collectively, these results establish FTase‐mediated nanobody‐PET probe assembly as a powerful platform for generating high‐quality imaging agents, with broad potential to support early disease detection, longitudinal disease monitoring, and personalized treatment guidance.

## 2 Results and Discussion

### 2.1 Design, Synthesis, and Molecular Docking of FTase Substrate Analogs

To develop nanobody‐based PET probes for in vivo imaging, site‐selective labeling was performed using FTase. Two substrate analogs were designed including a bifunctional analog (compound **1**) and a trifunctional analog (compound **2**). Both molecules feature a geranyl substructure with an allylic diphosphate allowing them to serve as FTase substrates and incorporate bioorthogonal groups for subsequent conjugation. Compound **1** includes an azide moiety for metal‐free strain‐promoted alkyne‐azide cycloaddition (SPAAC), while compound **2** contains both azide and aldehyde groups, enabling dual metal‐free reactions including SPAAC and oxime ligation with aminooxy‐based reagents. Docking studies showed that both compounds adopt conformations similar to the natural substrate FPP in the FTase active site (Figure 1c,d), and that the substrate binding pocket can accommodate the azide and 1,3,5‐trisubstituted aryl groups. Previous work with a related trifunctional analog indicated that ortho substitution near the phenoxide oxygen caused steric clashes, reinforcing the choice of a 1,3,5‐substitution pattern for compatibility with wild‐type FTase [59]. Compound **1** was synthesized following a previously published protocol [64]. Compound **2** was prepared from commercially available geraniol and 3‐bromo‐5‐hydroxybenzaldehyde in seven steps (Scheme S1).

### 2.2 The Expression and Refolding of Nanobody‐CVIA

To enable FTase‐mediated modification, the nanobody was engineered with a C‐terminal CaaX motif (CVIA). Nanobodies adopt an immunoglobulin β‐sandwich fold and contain a conserved intramolecular disulfide bond that stabilizes this structure and contributes to their high thermal and chemical stability. Accordingly, nanobodies are typically expressed in *Escherichia coli* (*E. coli*) using periplasmic expression systems to facilitate correct disulfide bond formation. Under these conditions, periplasmic expression commonly yields ∼5–10 mg of correctly folded nanobody per liter of bacterial culture. We initially attempted periplasmic expression of the nanobody‐CVIA using established protocols; however, repeated attempts failed to produce detectable protein. We reasoned that the additional cysteine introduced by the C‐terminal CaaX motif likely interfered with oxidative folding in the periplasm, leading to misfolding or degradation. We therefore pursued an alternative strategy based on cytoplasmic expression followed by in vitro refolding.

The nanobody–CVIA was expressed in *E. coli* as insoluble inclusion bodies, yielding high protein levels (>100 mg/L culture). Inclusion bodies were isolated, solubilized, and subjected to oxidative refolding using glutathione redox buffers with varying GSH/GSSG ratios (Figure S1a). Although several conditions produced soluble protein, a 5:1 GSH/GSSG ratio consistently gave properly folded and functional nanobody (Figure S1b). For comparison, the parental anti‐CD11b nanobody expressed by periplasmic secretion (**N11bp**) was purified and confirmed by SDS–PAGE (Figure S1f). Under the optimized refolding conditions, the crude refolded nanobody–CVIA directly from the refolding buffer showed strong binding to CD11b^+^ macrophages (Figure S1b). Using these conditions, the nanobody was then refolded and purified, and the purified product retained binding activity comparable to **N11bp** in a head‐to‐head affinity experiment, with *K*_D_ values of 2.57 nM for **N11b** and 3.22 nM for **N11bp** (Figure S2a,b). Overall, this refolding protocol was highly reproducible and enabled recovery of >70 mg/L of soluble, correctly folded nanobody–CVIA (Figure S1c).

### 2.3 FTase‐Catalyzed Site‐Selective Labeling of Anti‐CD11b VHH Using Bifunctional FPP Analog 1

Successful and selective incorporation of analog **1** into peptides and proteins by FTase has been previously reported [48, 51, 64]. To establish site‐specific VHH‐PET chelator conjugates for in vivo imaging, the refolded CVIA CaaX‐box‐containing anti‐CD11b nanobody (hereafter referred to as **N11b**) was used. The yeast FTase enzyme (EC number: 2.5.1.58; accession number α‐subunit: Q02293, β‐subunit: Q04631) was produced in *E. coli* and purified using Ni‐NTA affinity chromatography, following established protocols [65, 66]. Prior to generating the protein‐chelator conjugates, a proof‐of‐concept fluorophore‐labeled construct, **N11b‐TAM**, was synthesized to allow characterization by SDS‐PAGE and to enable visualization of nanobody binding to CD11b^+^ cells using confocal microscopy and flow cytometry. Accordingly, **1** (10 µM) was incubated with **N11b** (5 µM) in the presence of FTase (400 nM) and DTT (5 mM) for 3 h at 32°C yielding azide‐functionalized **N11b** (**N11b‐Az**, Figure 2a) with >98% conversion. Successful completion of the prenylation reaction was confirmed by LC‐ESI‐MS analysis, with MS spectra showing the unmodified **N11b** prior to reaction (Figure S3a) and a major peak corresponding to the prenylated **N11b‐Az** after reaction (Figure S3b). The **N11b‐Az** (50 µM) product was subsequently reacted with a strained alkyne‐bearing fluorophore, TAMRA‐DBCO (250 µM), via SPAAC for 1 h at r.t. to yield the **N11b‐TAM** conjugate (Figure 2b). Successful conjugation was further verified by LC‐ESI‐MS, which showed a mass shift consistent with TAMRA addition (Figure S3c). SDS‐PAGE analysis demonstrated increased electrophoretic mobility of the prenylated VHH (**N11b‐Az**) relative to the unmodified protein, a shift commonly observed for prenylated proteins in gel‐based assays [67, 68, 69]. Upon TAMRA addition, the protein (**N11b‐TAM**) exhibited reduced mobility, consistent with the increased molecular weight, and the corresponding band was fluorescent, confirming successful labeling (Figure 2c, lane 3–4). A higher molecular weight fluorescent band was observed only in the crude reaction sample (lane 3) and is attributed to nonspecific labeling of cysteine residues on FTase by excess TAMRA‐DBCO during SDS PAGE sample heating. This band is not observed after desalting, as removal of excess reagent prevents further nonspecific labeling. Accordingly, only desalted samples were used for all downstream experiments. Incubation of CD11b‐expressing RAW 264.7 macrophage cells with the **N11b‐TAM** construct demonstrated low‐nanomolar affinity binding (*K*_D_ = 2.13 nM, measured via flow cytometry), comparable to unmodified **N11b** (*K*_D_ = 2.57 nM, Figure S2b). The modest difference falls within the expected experimental variation, indicating that the addition of the prenyl group and fluorophore does not affect conjugate binding (Figure S4). Binding of **N11b‐TAM** to the macrophage cell surface was visualized by confocal microscopy using two detection channels: TAMRA fluorescence (Figure S5b) and anti‐His‐PE/Cy5 antibody labeling of the nanobody's His‐tag (Figure S5c). Merged images displayed light pink fluorescence, confirming colocalization of TAMRA (green) and PE/Cy5 (magenta) signals at the membrane (Figure S5d).

**FIGURE 2:**
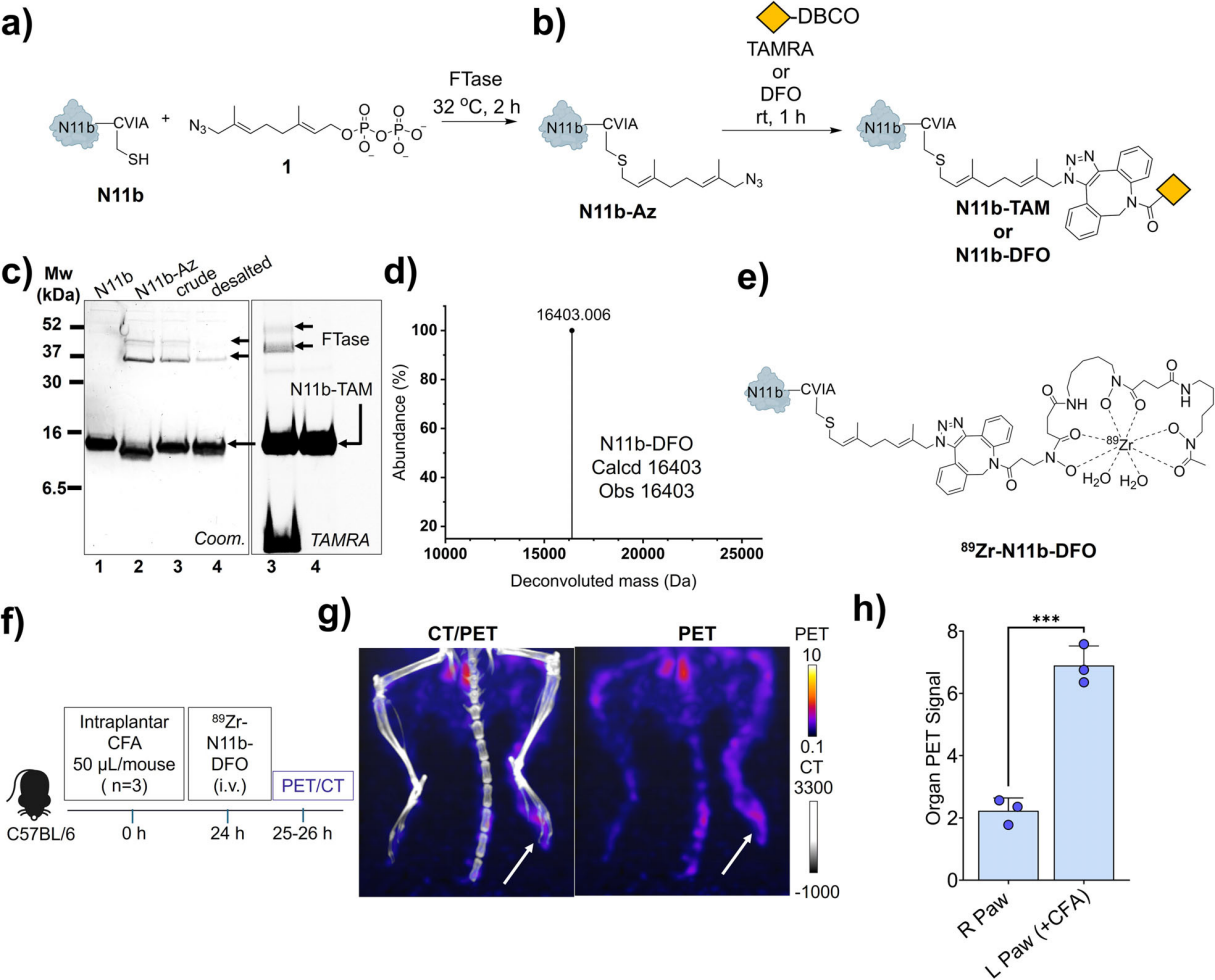
Probe assembly and in vivo imaging using a CD11b‐targeted PET reagent for detecting localized CFA‐induced paw inflammation. (a) Schematic representation of the synthesis of the **N11b‐Az**. (b) Schematic representation of the synthesis of the **N11b‐TAM** and **N11b‐DFO**. (c) SDS‐PAGE analysis of the protein constructs; left gel (Coomassie blue staining for total protein): Lane 1, unmodified **N11b**; Lane 2, azide‐modified **N11b** (**N11b‐Az**) obtained using substrate **1** (≥98% conversion); Lane 3, crude sample of fluorescent **N11b** (**N11b‐TAM**) after SPAAC reaction with TAMRA‐DBCO (≥99% conversion); Lane 4, desalted N11b‐TAM using PD‐10 prepacked columns; right gel: fluorescence scan of the previous gel, with Lane 3 and 4 showing a fluorescent band confirming TAMRA conjugation. Uncropped gels provided in supporting information. (d) Deconvoluted ESI‐MS of **N11b‐DFO** obtained after SPAAC reaction between **N11b‐Az** and DFO‐DBCO confirming product mass. (e) Structure of ^89^Zr‐labeled PET probe (**^89^Zr‐N11b‐DFO**). (f) Workflow for PET imaging experiments with **^89^Zr‐N11b‐DFO**. C57BL/6 mice received intraplanar CFA (*n* = 3) and were given 50 µCi of **^89^Zr‐N11b‐DFO** probe after 24 h and imaged ∼2 h later. (g) PET/CT MIP and PET only images of CFA‐treated mice show diffuse inflammation (white arrow) in the left paw. (h) Quantification of the SUV_mean_ of the **^89^Zr‐N11b‐DFO** probe in each organ in CFA‐treated (*n* = 3) mice reveals substantial difference between left paw uptakes ∼2 h post probe injection (SUV_mean_ 2.234 ± 0.409 and 6.899 ± 0.624, respectively. *P* = 0.0004) using Welch's t test. The PET and CT scale bars are in µCi mm^−3^ and Hounsfield units, respectively. In the bar graph, the bars show the SUV_mean_ and the error bars indicate the s.d. Significance is indicated as *P < 0.05, **P < 0.01, and ***P < 0.001.

### 2.4 Imaging CFA‐Induced Inflammation With ^89^Zr‐N11b‐DFO

To evaluate whether FTase‐based preparation of nanobody PET probes enables sensitive detection of localized inflammation, we employed a CFA‐induced paw inflammation model, a well‐established model of severe, localized inflammation, to assess probe sensitivity and in vivo imaging performance. Local CFA injection induces rapid recruitment of CD11b^+^ myeloid cells, particularly neutrophils and macrophages, making this model well suited for evaluating the CD11b‐targeting nanobody probe. To generate the radiolabeled tracer, the azide‐functionalized nanobody (**N11b‐Az**) was conjugated to DBCO‐DFO via SPAAC, yielding **N11b‐DFO** with near‐quantitative efficiency as confirmed by SDS‐PAGE analysis (Figure S6a). Successful conjugation was confirmed by LC‐ESI‐MS, which showed the expected mass shift corresponding to DFO addition and confirmed the absence of contaminating divalent metal ions that could interfere with ^89^Zr chelation (Figure 2d).

Next, the ability of the **N11b** PET probe to visualize CD11b positive myeloid cell‐rich organs was evaluated in vivo. DFO‐conjugated nanobodies (**N11b‐DFO**) were labeled with ^89^Zr yielding **^89^Zr‐N11b‐DFO** (Figures 2e and S28a peak at origin *R*_f_ < 0.1) with over 95% yield by radio‐thin‐layer chromatography (RadioTLC) and injected into healthy C57BL/6 mice. For PET imaging studies, **N11b‐DFO** containing residual FTase was used directly for radiolabeling. We note that purification by standard methods such as size‐exclusion chromatography (SEC) requires the use of Chelex resin‐treated, metal‐free buffers to avoid adventitious metal contamination of DFO, which can reduce radiochemical yield during ^8^ ^9^Zr labeling. Nevertheless, SEC analysis demonstrated effective separation of FTase (P1, eluting from 27.6–35 min) and **N11b‐DFO** (P2, eluting at ∼42 min), which was confirmed by SDS‐PAGE analysis of the corresponding fractions (Figure S6b,c). CD11b‐based PET imaging performed ∼2 h post‐injection enabled visualization of CD11b‐expressing myeloid cells, with prominent tracer accumulation observed in the spleen, inguinal lymph nodes, femoral bone marrow, and terminal ileum (Figure S7), supporting the suitability of this probe for CD11b‐targeted PET imaging. Subsequently, **^89^Zr‐N11b‐DFO** was evaluated in the CFA‐induced paw inflammation model. Mice received intraplantar CFA injection into the left foot prior to imaging (Figures 2f and S8a). PET imaging ∼26 h after CFA induction and ∼2 h after tracer administration revealed diffuse accumulation of the probe in the inflamed left paw (Figure 2g). Quantitative analysis showed that the SUV_mean_ of the CFA‐injected left foot was approximately threefold higher than that of the untreated contralateral right foot (6.90 ± 0.62 vs 2.23 ± 0.41, *p* < 0.0004; Figure 2h). Organ‐level biodistribution in CFA‐treated mice was comparable to that of healthy controls in immune‐associated tissues, including the spleen, inguinal lymph nodes, and femoral bone marrow, with additional tracer uptake localized at the inflamed footpad (Figure S8b).

### 2.5 FTase‐Catalyzed Dual Labeling of N11b With Trifunctional FPP Analog 2

To further enhance PET signal‐to‐noise ratios, PEGylation of nanobodies was evaluated as a general strategy to improve imaging performance. PEGylation is known to reduce renal retention and improve signal‐to‐noise ratios, albeit at the cost of extending circulatory half‐life in a size‐dependent manner, with larger PEG moieties conferring prolonged systemic retention [70, 71, 72, 73, 74, 75]. Using the trifunctional substrate **2**, PEGylated PET probes were synthesized through a streamlined three‐step process. Prenylation of the nanobody with **2** (10 µM) generated the dual‐labeled aldehyde–azide nanobody intermediate (**N11b‐AzAl**) in >95% conversion (Figure S9). For this reaction, **N11b** was first reduced with 50 mM DTT in ice for 30 min, followed by desalting to remove the reductant, and then incubated at 5 µM nanobody with 400 nM FTase. The removal of the reductant is a crucial step to prevent reduction of aryl azide to aryl amine.

Next, one‐pot dual labeling of **N11b‐AzAl** (50 µM) with 5 kDa aminooxy‐PEG (250 µM) and TAMRA‐DBCO or DFO‐DBCO (250 µM) via oxime ligation and SPAAC, respectively, afforded the desired conjugates **N11b‐PEG5‐TAM** and **N11b‐PEG5‐DFO** (Figure 3a). One‐pot oxime ligation and SPAAC reactions using 10 kDa PEG were evaluated using different catalysts, concentrations, and pH conditions. The SPAAC reaction with TAMRA‐DBCO proceeded efficiently under all tested conditions (>99% conversion), as evidenced by fluorescent protein bands (**N11b‐TAM‐Al**, Figures S10–S12) and their reduced mobility in the gel. In contrast, oxime ligation showed limited efficiency, with a maximum conversion of approximately 15%. Under standard conditions (reaction pH 6.0, 100 mM para‐phenylenediamine (pPDA) catalyst), the conversion was <5% (Figure S10). Among the catalysts tested, 10 mM anthranilic acid, 10 mM 3,5‐diaminobenzoic acid, 40 mM mPDA, and 200 mM pPDA, the anthranilate‐containing reaction gave the best result (∼10% conversion) (Figure S11). At pH 5.5, 5‐methoxyanthranilic acid at 50–100 mM yielded a similar maximum conversion of ∼15%, with no further improvement observed (Figure S12). The limited efficiency is likely due to the slower kinetics of oxime ligation with bulky aminooxy‐PEG, which necessitates the use of excess reagent, thereby increasing the viscosity of the reaction mixture and further reducing conversion. To address this, a small TCO‐containing spacer was introduced that enables a faster, more efficient inverse electron demand Diels‐Alder (IEDDA) reaction using lower equivalents (1.5 equiv.) of PEG reagent. For that, a one‐pot sequential reaction process was employed. First, a SPAAC reaction with TAMRA‐DBCO or DFO‐DBCO was performed followed by oxime ligation with an aminooxy‐TCO linker. The resulting intermediates, **N11b‐TAM‐TCO** and **N11b‐DFO‐TCO** (50 µM), subsequently underwent IEDDA reaction with 10 kDa mPEG‐tetrazine (75 µM) to yield the final products, **N11b‐PEG10‐TAM** and **N11b‐PEG10‐DFO** (Figure 3b). SDS‐PAGE analysis of the fluorescent nanobody conjugates showed a PEG size‐dependent decrease in electrophoretic mobility, with **N11b‐PEG10‐TAM** (>90% conversion) migrating the slowest, followed by **N11b‐PEG5‐TAM** (≥90% conversion), and the fastest migration observed for the non‐PEGylated **N11b‐TAM** (Figure 3c).

**FIGURE 3:**
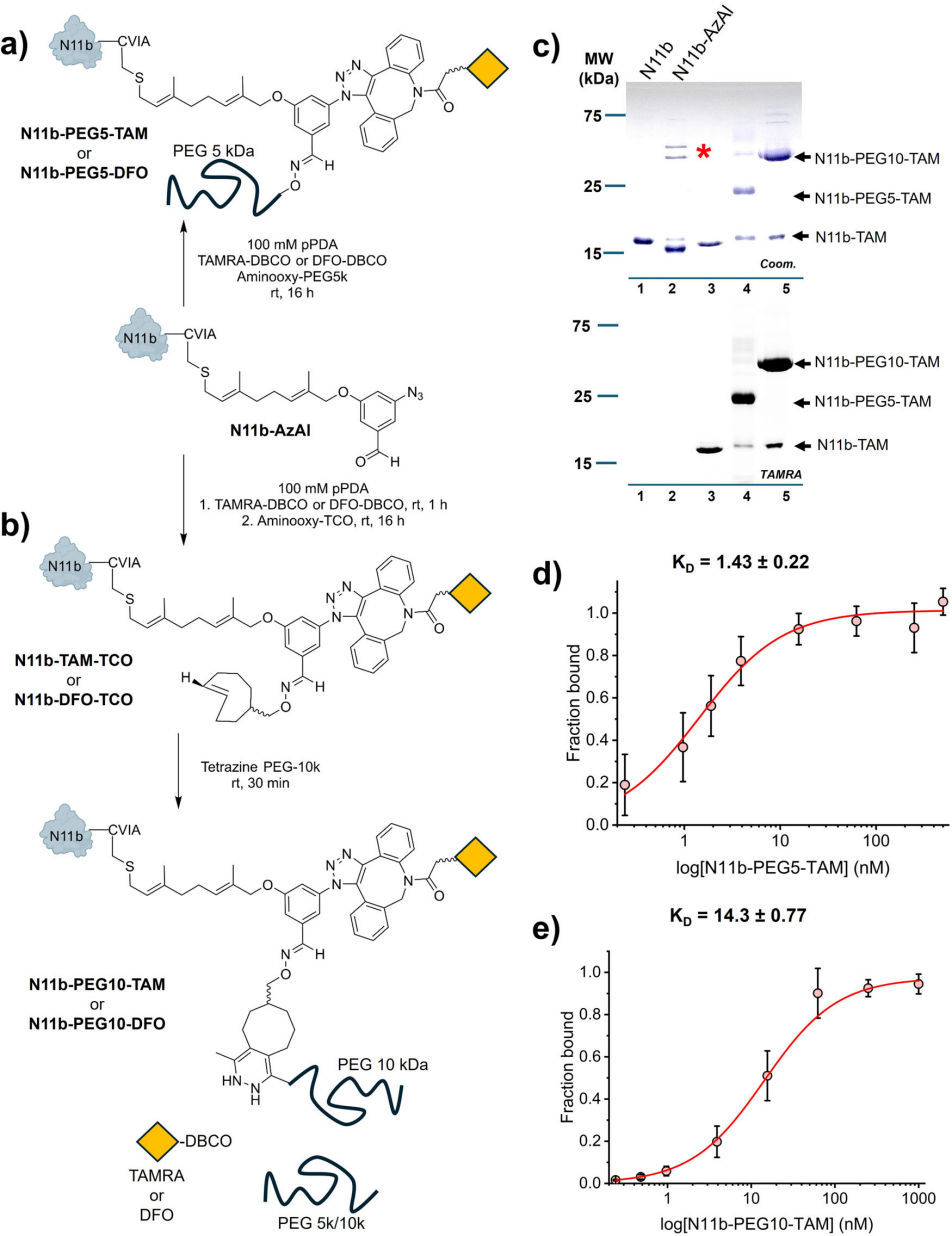
FTase‐catalyzed dual labeling of **N11b** using trifunctional FPP analog **2**. Enzymatic modification of **N11b** with **2** introduced two orthogonal reactive handles, an azide and an aldehyde, yielding **N11b‐AzAl**. (a) Schematic representation of one‐pot conjugation strategy for incorporating TAMRA or DFO and PEG 5 kDa. PEG 5 kDa was conjugated in a one‐pot reaction by SPAAC with TAMRA‐DBCO or DFO‐DBCO, followed by direct oxime ligation with aminooxy‐PEG 5 kDa. (b) Schematic representation of sequential conjugation strategy for incorporating TAMRA or DFO and PEG 10 kDa. Following SPAAC with TAMRA‐DBCO or DFO‐DBCO, a short TCO‐functionalized spacer was introduced via oxime ligation with aminooxy‐TCO (yielding **N11b‐TAM‐TCO or N11b‐DFO‐TCO**), followed by an inverse electron demand Diels‐Alder reaction with PEG 10 kDa‐tetrazine to generate **N11b‐PEG10‐TAM** or **N11b‐PEG10‐DFO**. (c) SDS‐PAGE analysis confirming PEGylation and TAMRA conjugation. Top: Coomassie‐stained gel. Lane 1: unmodified **N11b**; Lane 2: **N11b‐AzAl** (≥93% conversion); Lane 3: **N11b‐TAM** (≥99%); Lane 4: **N11b‐PEG5‐TAM** (≥91%); Lane 5: **N11b‐PEG10‐TAM** (≥90%). The red asterisk shows the FTase α (48 kDa) and β (34 kDa) subunit bands. Bottom: fluorescent gel scan showing TAMRA‐labeled bands, validating successful conjugation. Uncropped gels provided in supporting information. (d) and (e) Flow cytometry‐based affinity titration of **N11b‐PEG5‐TAM** and **N11b‐PEG10‐TAM** binding to CD11b on RAW 264.7 macrophages. Cells were incubated with increasing concentrations of PEGylated nanobodies, and TAMRA fluorescence was used to assess binding. Error bars indicate s.d.

Notably, when oxime ligation was first performed with the aldehyde present in **N11b‐AzAl** to generate **N11b‐Az‐TCO**, and subsequent execution of SPAAC and tetrazine ligation in a one‐pot format resulted in an unintended side reaction. Unreacted excess aminooxy‐TCO reagent from the oxime ligation step underwent a 1,3‐dipolar cycloaddition with the aryl azide on **N11b‐AzAl**, thereby preventing the intended SPAAC reaction. This interference was evidenced by the absence of the expected fluorescent band at ∼35 kDa in the SDS‐PAGE gel (blue arrow, Figure S13).

Importantly, the trifunctional linker strategy also enables efficient one‐pot dual labeling when the reaction pairings are judiciously selected. By leveraging the rapid kinetics of SPAAC, use of PEG‐DBCO in combination with aminooxy‐TAMRA allows simultaneous installation of both functionalities without cross‐reactivity. Indeed, reaction of **N11b‐AzAl** with DBCO‐PEG20 kDa and aminooxy‐TAMRA in a one‐pot format afforded **N11b‐PEG20‐TAM** with >97% conversion (Figure S14a–c), highlighting the versatility of this platform. These results highlight that reaction efficiency in such systems is governed not only by linker design but also by the relative kinetics of the conjugation chemistries. Notably, oxime ligation performs well with small molecules and short PEG reagents but becomes less efficient with larger, more sterically demanding PEG chains.

Despite this advantage, this route was not extended to PET tracer development in the present study. Translation to DFO‐based constructs requires aminooxy‐DFO, which is not currently commercially available, although synthetic routes have been reported [76]. More importantly, DFO incorporation requires stringent metal‐free conditions, as trace metal contaminants can occupy the chelator and significantly reduce radiochemical yield during subsequent ^8^ ^9^Zr labeling. Consequently, purification steps such as HPLC must be carried out using Chelex‐treated buffers, and all solvents and reagents must be similarly treated. In light of these considerations, we adopted an alternative strategy in which a short TCO linker was first installed at the aldehyde site, followed by PEGylation via tetrazine ligation, while DFO was introduced at the azide site using SPAAC.

Flow cytometry analysis of RAW 264.7 macrophages incubated with **N11b‐PEG‐TAM** constructs (5, 10, and 20 kDa) showed low‐nanomolar binding affinities. The PEG5 conjugate retained affinity comparable to unmodified **N11b** (*K*_D_ = 1.43 nM vs 2.57 nM; Figure S2b), whereas the PEG10 construct exhibited a moderate decrease (*K*_D_ = 14.3 nM, ∼6‐fold reduction; Figure 3d,e). The PEG20 construct showed a further decrease in affinity (*K*_D_ = 20.70 nM, ∼8‐fold reduction, Figure S14d). Overall, these results indicate that prenylation and subsequent PEGylation do not abrogate nanobody binding, although increasing PEG size progressively reduces affinity. Notably, despite reduced binding, the extended circulatory half‐life of higher molecular weight PEG constructs probably enhances PET imaging contrast by improving target accumulation and retention.

### 2.6 PEGylated N11b PET Probes Enhances Imaging of Inflammation

Following synthesis of the **N11b‐PEG5‐DFO** and **N11b‐PEG10‐DFO** containing 5 and 10 kDa PEG polymers (Figure 3a,b) they were subsequently labeled with ^89^Zr to yield PEGylated PET probes, **^89^Zr‐N11b‐PEG5‐DFO** and **^89^Zr‐N11b‐PEG10‐DFO** with over 95% radiochemical yield by RadioTLC (Figure S28b,c peaks at origin *R*_f_ < 0.1). As with the non‐PEGylated nanobody probes, the performance of these new imaging agents was evaluated in healthy mice and in the CFA‐induced paw inflammation model. Similar to the non‐PEGylated **N11b‐DFO** construct, the **N11b‐PEG5‐DFO** and **N11b‐PEG10‐DFO** proteins containing residual FTase were used directly for PET radiolabeling. Importantly, when stringent metal‐free conditions are employed, SEC can be employed as an additional purification step to purify the PEGylated proteins prior to labeling. For **N11b‐PEG5‐DFO**, SEC provided effective separation of FTase (P1, 27.6–35 min) and **N11b‐PEG5‐DFO** (P2, ∼34 min), affording the target fraction at ∼89% purity with only trace FTase carryover. The non‐PEGylated **N11b‐DFO‐Al** species (P3, ∼42 min) was efficiently removed. These assignments were confirmed by SDS‐PAGE analysis of the corresponding fractions (Figure S15a,b). Likewise, for **N11b‐PEG10‐DFO**, SEC enabled clear separation of non‐PEGylated **N11b‐DFO‐Al** (∼42 min) from **N11b‐PEG10‐DFO** (∼32 min, P1), consistent with the SEC trace and SDS‐PAGE analysis of the crude sample (lane 5) and P1 fraction containing pure **N11b‐PEG10‐DFO** (lane 4) (Figure S15c,d).

For **N11b‐PEG10‐DFO**, residual FTase was less readily distinguished because its SEC retention time and SDS‐PAGE mobility overlapped with those of the PEGylated nanobody construct. In contrast, for **N11b‐PEG5‐DFO**, SEC afforded substantial purification of the target product (∼89% purity), with only trace FTase carryover. Accordingly, for PEG10 constructs, and for PEG5 constructs when higher purity is desired, FTase is best removed by anti‐FLAG affinity purification, since only the nanobody carries the FLAG tag (Figure S16). After SEC separation of PEGylated and non‐PEGylated nanobody species, anti‐FLAG purification can be used as an additional step to eliminate residual FTase. SEC remains particularly important for removing the non‐PEGylated nanobody counterparts, which also contains the FLAG tag and therefore cannot be separated from the PEGylated product by anti‐FLAG affinity purification alone. Removal of excess PEG reagents from protein conjugates is similarly best achieved by an orthogonal purification method such as cation‐exchange chromatography (e.g., SP‐Sepharose), as reported previously [70, 77]. SEC alone is not sufficient because PEG has a large effective hydrodynamic volume arising from its flexible, highly hydrated ethylene oxide units, causing free PEG to elute similarly to PEGylated proteins. In this study, the PEG reagents used for PET probe synthesis were aminooxy‐mPEG 5 kDa (5 equiv relative to nanobody) for oxime ligation and tetrazine‐mPEG 10 kDa (1.5 equiv) for IEDDA. Because aminooxy‐mPEG 5 kDa shows minimal absorbance at 280 nm, the corresponding tetrazine‐mPEG 5 kDa reagent was used for SEC comparison. SEC analysis showed that tetrazine‐mPEG 5 kDa and tetrazine‐mPEG 10 kDa eluted at 35.5 and 31.5 min, respectively, closely matching the retention times of the corresponding PEGylated nanobody conjugates **N11b‐PEG5‐DFO** and **N11b‐PEG10‐DFO** (Figure S17). Given that the principal size difference between these conjugates arises from the appended PEG chain, this SEC behavior strongly supports successful installation of PEG of the expected molecular weight onto the nanobody. This interpretation is further supported by the corresponding PEG size‐dependent shifts observed by SDS‐PAGE. At the same time, the overlap in retention behavior between free PEG and PEGylated nanobody indicates that excess PEG cannot be reliably removed by SEC alone and instead requires an affinity‐based purification step; in our workflow, anti‐FLAG affinity purification enabled selective recovery of nanobody species. Thus, the purification workflow consists of anti‐FLAG affinity purification to remove residual FTase and excess PEG reagents, followed by SEC to separate PEGylated from non‐PEGylated nanobody species and isolate the desired DFO‐labeled PEGylated product.

Densitometric analysis of the crude SDS‐PAGE samples prior to purification (lane 7, Figure S15b; lane 5, Figure S15d) showed that the DFO‐labeled nanobody fraction used for PET imaging was predominantly PEGylated. For **N11b‐PEG5‐DFO**, the nanobody‐containing fraction (PEGylated plus non‐PEGylated, that is, **N11b‐PEG5‐DFO** and **N11b‐Al‐DFO**) accounted for ∼85% of the sample, of which 90% corresponded to **N11b‐PEG5‐DFO** and only 10% to **N11b‐Al‐DFO**; the remaining material was FTase. For **N11b‐PEG10‐DFO**, the amount of residual FTase could not be reliably resolved by SDS‐PAGE because of overlapping electrophoretic mobility. However, assuming a similar level of FTase carryover as observed for **N11b‐PEG5‐DFO**, we estimate that the nanobody fraction comprised ∼85% of the sample, with 96% present as **N11b‐PEG10‐DFO** and only 4% as **N11b‐Al‐DFO**. Importantly, both the PEGylated and non‐PEGylated nanobody species carry DFO, as evidenced by the gel shift relative to **N11b‐AzAl** (Figure S15), indicating that ^89^Zr labeling occurs on both populations. This is a key advantage of the system, as it minimizes the presence of unlabeled nanobody in the final preparation and thereby reduces the risk of receptor blocking by nonradioactive protein.

The **^89^Zr‐N11b‐PEG10‐DFO** probe was administered to healthy mice, where it localized predominantly to immune cell‐rich tissues, as expected (Figure S18). Next, C57BL/6 mice were injected intraplantarly with CFA to induce localized inflammation in the left paw. At 12 h post‐CFA administration, mice were intravenously injected with ∼50 µCi of **^89^Zr‐N11b‐PEG10**, and PET/CT imaging was conducted at 12 h and 24 h post‐injection to assess probe distribution and inflammation‐specific uptake (Figure 4a). PET/CT and PET‐only images showed increased tracer uptake in the inflamed left paw (white arrow) at both 12 h and 24 h post‐injection time points (Figure 4b, Supporting videos SV1 and SV2).

**FIGURE 4:**
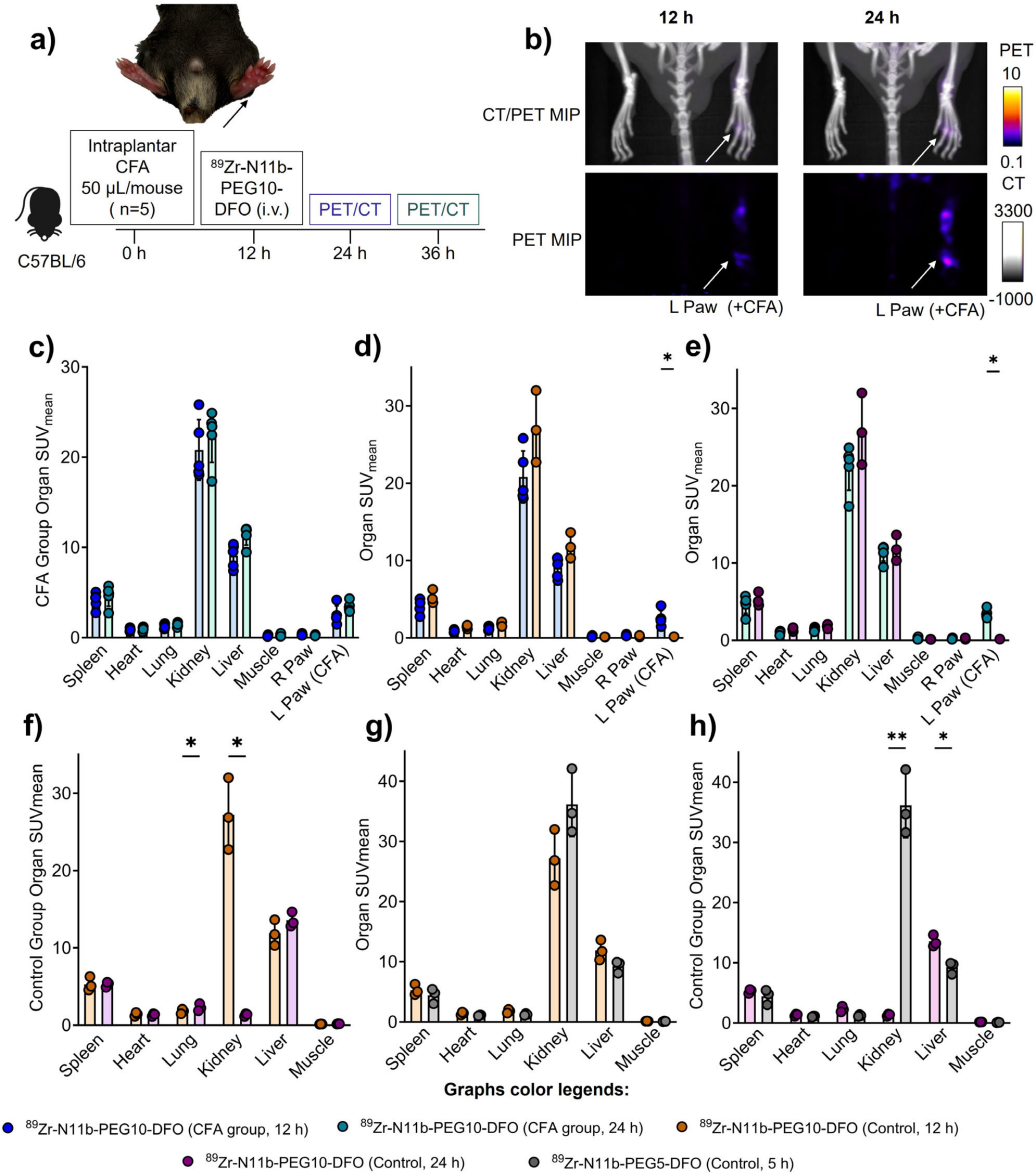
Use of **^89^Zr‐N11b‐PEG10‐DFO** to detect CFA‐induced paw inflammation and assessment of biodistribution. C57BL/6 mice received intraplantar CFA in the left paw (*n* = 5) and were injected intravenously with 50 µCi of **^89^Zr‐N11b‐PEG10‐DFO** 12 h later, followed by PET imaging at 12 h and 24 h post‐injection. Healthy C57BL/6 mice (*n* = 3) were injected intravenously with 50 µCi of **^89^Zr‐N11b‐PEG5‐DFO** and imaged 5 h later as controls. (a) Experimental scheme for CFA induction and tracer administration. (b) Representative PET/CT and PET‐only maximum‐intensity projection (MIP) images showing increased tracer uptake in the inflamed left paw (white arrow) at 12 h and 24 h post‐injection. PET and CT scale bars are in µCi mm^−^^3^ and Hounsfield units, respectively. (c) Organ‐wise SUV_mean_ quantification in CFA‐treated mice imaged with **^89^Zr‐N11b‐PEG10‐DFO** at 12 h and 24 h post‐injection (n = 5; multiple Wilcoxon matched‐pairs rank test). (d) Comparison of organ‐wise SUV_mean_ between CFA‐treated (n = 5) and control mice (n = 3) at 12 h post‐injection (Welch's t‐test). (e) Comparison of organ‐wise SUV_mean_ between CFA‐treated (*n* = 5) and control mice (*n* = 3) at 24 h post‐injection (Welch's t‐test). (f) Organ‐wise SUV_mean_ in control mice following **^89^Zr‐N11b‐PEG10‐DFO** administration (*n* = 5; paired t test). (g) Comparison of organ‐wise SUV_mean_ between **^89^Zr‐N11b‐PEG5‐DFO** (n = 3) and **^89^Zr‐N11b‐PEG10‐DFO** (*n* = 3) in control mice at 12 h post‐injection (multiple Mann‐Whitney test). (h) Comparison of SUV_mean_ between **^89^Zr‐N11b‐PEG5‐DFO** (*n* = 3) and **^89^Zr‐N11b‐PEG10‐DFO** (*n* = 3) probes in each organ in control mice. In the bar graphs, the bars show the mean SUV_mean_ and the error bars indicate the s.d. Significance is indicated as *P < 0.05, **P < 0.01, and ***P < 0.001.

In the CFA model, quantification of the SUV_mean_ at the site of inflammation (left paw) revealed significantly elevated probe uptake compared to the corresponding paw in control animals, approximately 12‐fold higher at 12 h (2.55 ± 0.99 vs. 0.21 ± 0.03, *p* =  0.0061) (Figures 4d and S19a) and ∼28‐fold higher at 24 h (3.44 ± 0.55 vs. 0.12 ± 0.05, *p* = 0.0002) (Figures 4e and S19b). The SUV_mean_ in inflamed paws between 12 and 24 h did not significantly differ, suggesting a plateau in accumulation at the target within this timeframe (Figure 4c). However, comparison of the left‐to‐right paw SUV_mean_ ratios highlighted stronger PET image contrast in CFA‐treated mice at 24 h. At 12 h, the CFA: healthy paw ratio was ∼7.5‐fold higher than the control mice left paw: right paw ratio (6.88 ± 2.09 vs. 0.92 ± 0.30, P =  0.0027), increasing to ∼16‐fold at 24 h (17.26 ± 5.28 vs. 1.06 ± 0.26, P =  0.0023), supporting an improved signal‐to‐noise ratio at the later time point (Figure S19c,d). Biodistribution analysis of **^89^Zr‐N11b‐PEG10‐DFO** in CFA‐treated mice revealed no significant differences in organ uptake between the 12 h and 24 h imaging timepoints, except for the kidney. A marked decline in kidney retention was observed, from an SUV_mean_ of 27.19 ± 4.65 at 12 h to 1.35 ± 0.18 at 24 h (P = 0.0104), suggesting substantial renal clearance by 24 h, which likely contributes to the enhanced imaging contrast (Figure 4f).

Next, to compare the influence of PEG size, healthy mice were administered **^89^Zr‐N11b‐PEG5‐DFO** (*n* = 3) and imaged 5 h post‐injection (Figure S20). While organ‐level uptake patterns were largely comparable between the PEG5 and PEG10 agents, important differences were observed in kidney and liver retention when each agent was evaluated at its respective optimal imaging time point (5 h for PEG5 and 12–24 h for PEG10). The PEG5 tracer at 5 h showed substantially higher kidney retention than the PEG10 tracer at 24 h (36.16 ± 5.36 vs. 1.35 ± 0.18, *p* = 0.0078), consistent with lower kidney retention of the PEG10 construct at the later imaging time point. In contrast, kidney retention of the PEG5 tracer at 5 h was not significantly different from that of the PEG10 tracer at 12 h, indicating that the more pronounced reduction is observed for PEG10 by 24 h, consistent with slower renal clearance and prolonged circulation of the higher‐molecular‐weight PEG construct. In the same control mice, PEG10 also showed greater liver accumulation at 24 h than at 12 h (13.56 ± 0.98 vs. 9.27 ± 1.01, *p* = 0.0061) (Figure 4g,h). These results underscore how PEG size modulates in vivo biodistribution and clearance profiles of the nanobody PET probe. Overall, these results demonstrate that FTase‐mediated, site‐specifically PEGylated and radiolabeled CD11b nanobodies enable clear detection of inflammation in vivo with both PEG5 and PEG10 modifications, and that PEGylation enhances image quality in a size‐dependent manner by modulating the pharmacokinetic properties of the probe.

Conventional maleimide–cysteine chemistry has been employed for PEGylating proteins and has been applied to nanobodies [70] and interleukins [77] through introduction of engineered cysteine residues for installation of linear or branched PEG chains ranging from 10 to 40 kDa. Although these studies demonstrated effective purification of PEGylated products, reaction conversions or efficiency were not clearly reported. Importantly, maleimide conjugation efficiency depends strongly on target cysteine pKa and accessibility. Consistent with this, our initial attempts to modify the C‐terminal cysteine of the nanobody (**N11b**) with maleimide‐PEG10 kDa (Figure S21a) gave poor product conversion (**N11b‐PEG10**) at pH 7.4 of ∼15%. Increasing the pH to 8.5 improved conversion to 23% but also led to formation of higher‐molecular‐weight species (∼20%), potentially arising from nonspecific side reactions (Figure S21b,c). These reactions were performed using 100 µM **N11b** and 10 equivalents of maleimide reagent (1 mM). Although further optimization of protein concentration, reagent stoichiometry, and reaction time may improve the outcome, the current results point to an inherent limitation of direct maleimide‐based PEGylation in this system, likely arising from the poor reactivity of the cysteine, steric bulk of the PEG chain together with the increased solution viscosity of high‐molecular‐weight PEG reagents, all of which can diminish conjugation efficiency. In addition to low conversion, maleimide‐based labeling can introduce product heterogeneity. Because maleimide reagents react indiscriminately with accessible thiols, labeling can generate heterogeneous products with multiple sites of modification and, if CDR‐associated cysteines are involved, may also compromise antigen binding. Moreover, cysteine‐maleimide chemistry enables installation of only a single functionality at a time, such that either PEG or DFO can be introduced, but not both simultaneously. Thus, even when bifunctional PEG reagents such as N_3_‐PEG‐maleimide (10 kDa) are used, access to dual‐labeled products still requires a multistep process. In contrast, our FTase‐based strategy enables efficient, site‐selective dual labeling, providing PEGylated, DFO‐containing nanobody constructs in ≥90% conversion using low‐micromolar substrate concentrations. Additionally, due to this modular approach, multiple conjugates can be produced from a single protein functionalized with the trifunctional substrate **2** using different combinations of alkyne and aminooxy reagents. Together, these results underscore the practical and mechanistic advantages of the FTase platform for rapid generation of dual‐functional nanobody imaging agents.

### 2.7 N45 PET Probe Enables High‐Sensitivity Visualization of Lymphoid Organs and Inflammation

Our **N11b** PET probe provides a more selective readout of CD11b^+^ myeloid cells. However, like many other PET probes that target a restricted subset of immune cells, such as CD8^+^ T cells [78, 79, 80] or CD206^+^ macrophages [81], **N11b** PET probes remain limited to CD11b^+^ myeloid cells [82]. To address this, we extended our FTase‐mediated strategy to generate PEGylated CD45‐targeting nanobody PET agents. CD45 is stably and highly expressed across both lymphoid and myeloid lineages. Its restricted expression on hematopoietic cells, together with its non‐internalizing nature [83], enables CD45‐targeted PET imaging to provide a sensitive and immune‐specific readout of inflammatory burden across diverse disease contexts. Within this framework, CD45‐targeted imaging could identify immune‐cell infiltration, followed by specific **N11b** imaging to define CD11b+ myeloid involvement.

An anti‐CD45 nanobody engineered with a C‐terminal CVIA sequence (denoted **N45**) was generated following protocols analogous to those used for **N11b**, yielding ≥70 mg/L of protein from *E. coli* liquid culture. The purified N45 was characterized by SDS–PAGE (Figure S1d,e) and LC–ESI–MS (Figure S22a), which revealed a minor disulfide‐linked glutathione adduct in the refolded stock protein; this was reduced with 50 mM DTT, followed by removal of the reductant prior to FTase modification to prevent reduction of the aryl azide in substrate **2** to the corresponding aryl amine. Binding to CD45 epitope was evaluated by flow cytometry‐based affinity titration, showing affinities comparable to the periplasmically expressed counterpart (**N45p**) in a head‐to‐head comparison (Figures S1e and S2c,d). Using the trifunctional FPP analog **2**, **N45‐AzAl** was generated and its structure confirmed by LC‐ESI‐MS (Figure S22b). Enzymatic modification with **2** enabled the synthesis of **N45‐PEG10‐TAM**, following the same procedures as for **N11b‐PEG10‐TAM**, and was analyzed by SDS‐PAGE. The appearance of a slower‐migrating, higher molecular weight fluorescent band near 37 kDa indicated successful PEGylation via IEDDA and TAMRA incorporation via SPAAC, with ≥96% conversion (Lane 3, Figure S23). Similarly, **N45‐PEG10‐DFO** was synthesized and characterized by SDS‐PAGE, achieving ≥98% conversion (Figure 5a). Flow cytometry analysis of RAW 264.7 macrophages incubated with **N45‐PEG10‐TAM** showed low‐nanomolar binding affinity (K_D_ = 8.3 nM, ∼2.4 fold reduction, Figure 5b), comparable to unmodified **N45** (*K*_D_ = 3.48 nM, Figure S2d). In addition, **N45‐PEG20‐TAM** was generated using the same strategy as for **N11b‐PEG20‐TAM** with ≥97% conversion and confirmed by SDS‐PAGE (Figure S14b), with a measured *K*_D_ of 16.77 nM (Figure S14e). These results indicate, as observed with **N11b**, that prenylation and subsequent PEGylation do not compromise the inherent binding functionality of the **N45** constructs.

**FIGURE 5:**
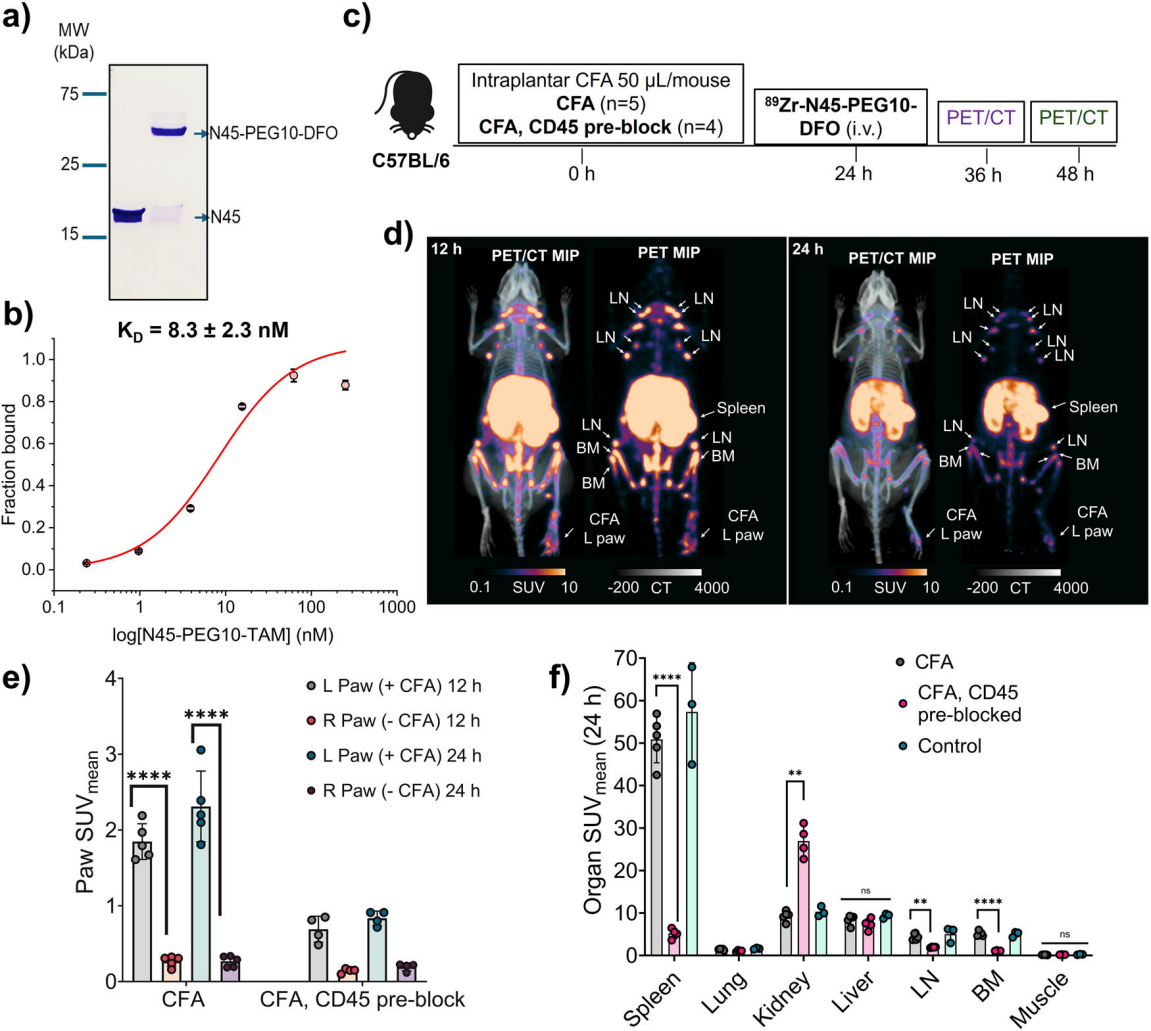
**^89^Zr‐N45‐PEG10‐DFO** detects immune cells in lymphoid organs and at the inflamed paw with high sensitivity. (a) SDS‐PAGE analysis of **N45‐PEG10‐DFO** conjugate generated by FTase‐mediated modification with trifunctional substrate **2** shows ≥98% conversion to product. Coomassie stain. Lane 1, unmodified **N45**; Lane 2, **N45‐PEG10‐DFO**. Uncropped gels provided in supporting information. (b) Flow cytometry‐based affinity titration of **N45‐PEG10‐TAM** binding to CD45 on RAW 264.7 macrophages. Cells were incubated with increasing concentrations of **N45‐PEG10‐TAM**, and TAMRA fluorescence was used to assess binding. Error bars indicate s.d. (c) PET imaging workflow. C57BL/6 mice received intraplantar CFA (*n* = 5) or intraplantar CFA following CD45 blockade with unmodified **N45** (*n* = 4). The mice were given ∼50 µCi of **^89^Zr‐N45‐PEG10‐DFO** probe intravenously 24 h after receiving CFA and imaged 12 h and 24 h post‐injection time points. (d) PET/CT MIP and PET‐only images of CFA‐treated mice at 12 h and 24 h time points. Clear uptake in lymphoid organs enriched in CD45^+^ immune cells is noted as well as at the inflammation site in the left paw (white arrows). (e) Quantification of SUV_mean_ (after **^89^Zr‐N45‐PEG10‐DFO** treatment) in the CFA‐induced inflamed left paw and control right paw both at 12 h and 24 h timepoints (Welch's t test) for two treatment groups, CFA treated and CFA treated with CD45 blocked. Grey bar: L paw (+CFA, 12 h); pink bar: R paw (−CFA, 12 h); teal bar: L paw (+CFA, 24 h); violet bar: R paw (−CFA, 24 h). (f) Comparison of organ‐wise SUV_mean_ values between CFA‐treated, CFA treated with CD45 blocking, and control mice (*n* = 3) imaged with **^89^Zr‐N45‐PEG10‐DFO** at 24 h imaging time point (Welch's t test). Grey bar: CFA treated; pink bar: CFA treated with CD45 blocking; teal bar: healthy mice without CFA treatment serving as controls. In both the bar graphs, the bars show the SUV_mean_ and the error bars indicate the s.d. LN: lymph node; BM: bone marrow. Error bars indicate s.d. The PET and CT scale bars are in µCi mm^−3^ and Hounsfield units, respectively. Significance is indicated as *P < 0.05, **P < 0.01, and ***P < 0.001.

Next, ^89^Zr‐labeled **N45‐PEG10‐DFO** (**^89^Zr‐N45‐PEG10‐DFO**) was prepared and evaluated as a PET imaging agent to assess its ability to detect inflammation. As with the **N11b** PET probes, SEC purification was not performed for the **N45** constructs prior to radiolabeling. However, SEC analysis demonstrated efficient removal of non‐PEGylated **N45** (42 min) from PEGylated N45 (32 min, P1), as supported by the SEC trace (Figure S24a) and SDS‐PAGE of the crude (lane 4) and purified fractions (P1, lane 3) (Figure S24b). Owing to the high and ubiquitous expression of CD45 on immune cells, the **N45**‐based tracer exhibited markedly enhanced sensitivity for imaging inflammation and immune‐rich tissues compared to the **N11b**‐based imaging agent. For in vivo PET imaging, three groups of C57BL/6 mice were examined: (i) mice receiving intraplantar CFA injection in the left paw to induce localized inflammation, (ii) CFA‐treated mice pre‐blocked with excess unmodified **N45** to competitively inhibit CD45 binding, and (iii) healthy mice without CFA treatment serving as controls. The CD45‐blocked cohort was included to directly assess the specificity of tracer uptake and to confirm that the observed PET signal arises from CD45‐mediated binding rather than nonspecific accumulation. Mice were administered CFA intraplantarly in the left paw, followed by intravenous injection of the imaging agent 24 h later. PET imaging was performed at 12 h and 24 h post‐injection, with no significant differences observed between these two time points, indicating stable tracer distribution and target engagement over this period (Figure 5c,d).

In the CFA‐treated group, pronounced tracer accumulation was observed at the inflamed left paw. At 12 h post‐injection, the SUV_mean_ of the left paw was approximately 7‐fold higher than that of the contralateral right paw (1.85 ± 0.24 vs 0.27 ± 0.07, *p* = 0.00009). This difference increased to nearly 9‐fold at 24 h post‐injection (2.31 ± 0.47 vs 0.27 ± 0.07, *p* = 0.0004). These results indicate robust and selective accumulation of the tracer at sites of inflammation, consistent with increased immune cell infiltration and elevated CD45 expression (Supporting videos SV3 (PET/CT) and SV4 (PET‐only). In contrast, mice in the CD45‐blocked CFA group exhibited markedly reduced tracer uptake at the inflamed site, with no significant difference between left and right paws (Figure S25). At 12 h post‐injection, SUV_mean_ values were 0.69 ± 0.17 (left) and 0.14 ± 0.03 (right), while at 24 h they were 0.84 ± 0.09 (left) and 0.19 ± 0.05 (right) (Figure 5e). The substantial attenuation of signal following CD45 blockade strongly supports a CD45‐specific uptake mechanism and confirms that the enhanced PET signal observed in the CFA group is driven by targeted binding rather than nonspecific inflammation‐associated accumulation. At the organ level, tracer uptake profiles were comparable at 12 h and 24 h post‐injection, indicating stable biodistribution. The healthy control and CFA‐treated groups showed similar overall organ uptake, with the CFA‐treated group additionally exhibiting strong signal at the inflamed left paw. In contrast, the CD45‐blocked CFA group displayed markedly reduced uptake across immune‐rich organs, consistent with effective target blockade. In this group, PET signal was dominated by kidney accumulation, reflecting renal clearance of unbound tracer, and kidney SUV_mean_ values were significantly higher than those observed in the CFA‐treated group. For both the CFA‐treated and control groups, strong tracer accumulation was observed in immune cell‐rich tissues, including the spleen, lymph nodes, and bone marrow, consistent with CD45 expression on immune cells in these organs and reinforcing the specificity of the imaging agent for CD45^+^ immune cells (Figures 5f and S26). Collectively, these results demonstrate that the FTase‐mediated **^89^Zr‐N45‐PEG10‐DFO** PET probe enables sensitive and specific CD45‐targeted imaging, supporting CD45‐PET as a broadly applicable approach for detecting diverse leukocyte populations without compromising sensitivity relative to immune cell subset‐restricted tracers.

Given the robustness of the PET agents generated via our FTase‐mediated strategy, it is important to delineate the respective roles of PEGylation and target selection (CD11b vs CD45) in governing imaging specificity, sensitivity, and signal‐to‐background ratios. PEGylation of **N11b** with a 10 kDa PEG moiety significantly enhanced imaging sensitivity by increasing target accumulation, consistent with the prolonged circulation half‐life of PEGylated PET agents compared to their non‐PEGylated counterparts (Figure S27a,b). In addition to strong signal at the CFA‐induced footpad inflammation site, uptake was observed in lymph nodes and bone marrow. Although **N11b** and **N45** exhibit comparable binding affinities toward their respective CD11b and CD45 targets (Figure S2b,d), head‐to‐head comparisons of PEGylated **N11b** and PEGylated **N45** constructs bearing identical PEG sizes revealed a marked difference in in cellulo and in vivo performance. Confocal imaging of RAW 264.7 cells with **N11b‐PEG10‐TAM** and **N45‐PEG10‐TAM** showed that, in both cases, fluorescence was localized predominantly to the cell membrane rather than intracellular compartments, confirming that the PEGylated nanobodies remained surface‐bound and were not internalized, while also exhibiting high signal‐to‐background ratios. This provides information complementary to flow cytometry, which quantifies overall fluorescence intensity but does not resolve subcellular localization. Within the confocal images, **N45‐PEG10‐TAM** exhibited markedly stronger TAMRA fluorescence than **N11b‐PEG10‐TAM** (Figure 6b,c), consistent with the higher surface abundance of CD45 relative to CD11b on RAW 264.7 cells [84]. This difference was further reflected in flow cytometry of the 20 kDa PEG constructs, where **N45‐PEG20‐TAM** gave a substantially higher TAMRA mean fluorescence intensity (MFI = 22,923) than **N11b‐PEG20‐TAM** (MFI = 6,736), corresponding to an approximately 3.4‐fold difference (Figure 6d). Consistent with these observations, the **^89^Zr‐N45‐PEG10‐DFO** enabled comprehensive visualization of immune‐rich organs, including the spleen, bone marrow, and lymph nodes, in addition to the inflamed footpad (Figure S27b,c). In contrast, **^89^Zr‐N11b‐PEG10‐DFO** generated a less complete view of immune‐rich tissue.

**FIGURE 6:**
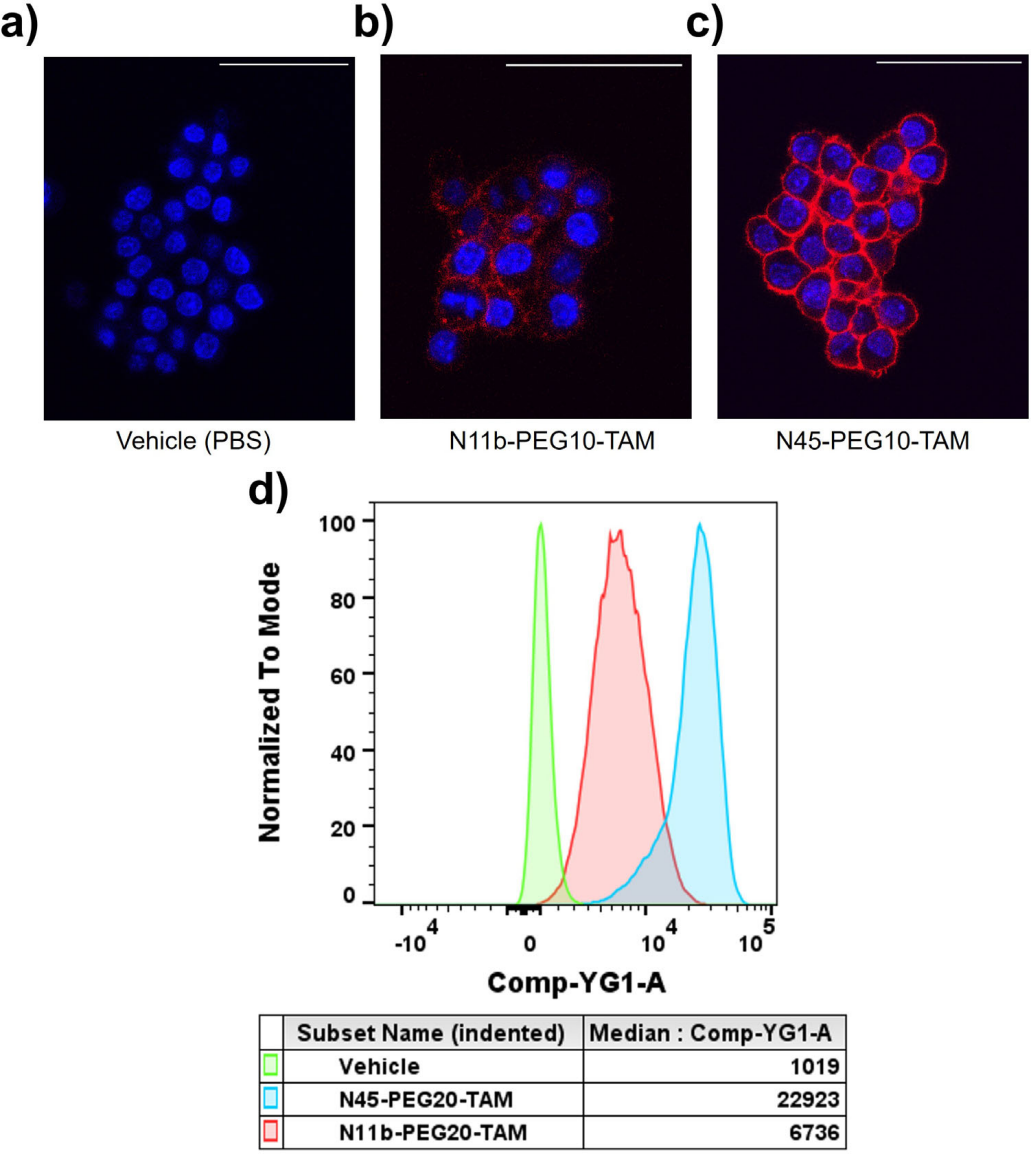
Comparative binding of N11b and N45 to immune cells assessed by confocal microscopy and flow cytometry. Confocal imaging of CD11b^+^ and CD45^+^ RAW 264.7 macrophages showing differential binding of N11b‐PEG10‐TAM and N45‐PEG10‐TAM based on TAMRA fluorescence. (a) Cells treated with PBS only. (b) Cells treated with 150 nM N11b‐PEG10‐TAM. (c) Cells treated with 150 nM N45‐PEG10‐TAM. After incubation with the protein constructs, cells were washed and stained with Hoechst 34580 to visualize nuclei. Nuclei are shown in blue and TAMRA fluorescence in red. Scale bar, 50 µm. (d) Flow cytometric analysis of RAW 264.7 cells incubated with 500 nM N11b‐PEG20‐TAM or N45‐PEG20‐TAM. After washing, TAMRA fluorescence was measured using a 561 nm yellow‐green laser with the YG1 channel (577/20 nm bandpass filter). Mean fluorescence intensity (MFI) values are listed in the table: N45‐PEG20‐TAM: 22,923; N11b‐PEG20‐TAM: 6,736; Vehicle: 1019.

The dual‐labeling strategy reported here provides a compelling foundation for the clinical translation of nanobody‐based inflammation imaging agents by enabling high‐efficiency installation of both PEG and a radiometal chelator in only two steps. From a manufacturing perspective, protein A affinity chromatography is an established platform capture method for antibody therapeutics, whereas peptide affinity tags such as His or FLAG are primarily suited to research‐scale purification and are less favorable for clinical production due to scalability and regulatory considerations. Synthetic nanobody platforms incorporating protein A‐binding frameworks enable clinical translation of this workflow by replacing anti‐FLAG capture with protein A purification after dual labeling, followed by SEC to isolate pure DFO‐labeled PEGylated PET probes. Together, these features underscore the modularity, manufacturability, and translational potential of this trifunctional linker platform for the development of clinically relevant nanobody‐based PET imaging agents for inflammation.

## 3 Conclusion

In this work, we developed a streamlined chemoenzymatic platform based on FTase for the efficient generation of nanobody‐based PET imaging agents. Using nanobodies targeting CD11b and CD45 to establish this approach, high production yields and favorable in vivo imaging profiles were obtained for both probes. This approach leverages short, readily synthesized, aqueous‐stable isoprenoid diphosphate substrates and enables near‐quantitative (>95%) site‐specific modification under mild conditions within hours with minimal substrate input, providing a practical and cost‐efficient alternative to existing enzymatic labeling strategies. The use of bifunctional and trifunctional substrates further expands modularity, allowing precise installation of PET chelators and auxiliary handles, such as PEG, without compromising protein integrity.

The nanobody‐based PET probes were evaluated in both healthy mice and a CFA‐induced paw inflammation model, which represents a well‐established model of severe, myeloid‐driven localized inflammation. As expected, the CD11b‐targeted nanobody enabled clear visualization of the inflamed tissue. Notably, the CD45‐targeted nanobody also robustly detected inflammation in this model, consistent with high CD45 expression on neutrophils and other infiltrating immune cells. Quantitative analysis revealed higher tracer uptake and SUVs for CD45‐PET compared with CD11b‐PET, demonstrating that even in a predominantly myeloid‐driven inflammatory setting, pan‐leukocyte targeting can provide superior sensitivity relative to immune subset‐restricted tracers.

To further assess the specificity of FTase‐mediated nanobody PET probe generation, we performed in vivo blocking studies using excess unlabeled CD45 nanobody, which resulted in a substantial reduction in CD45‐PET signal. Incomplete blocking is likely attributable to the rapid clearance of the unlabeled nanobody (within ∼1–2 h), whereas the PEGylated PET probe exhibits a prolonged circulation half‐life, allowing it to competitively bind CD45 molecules over time. In addition, ongoing immune cell turnover and de novo CD45 expression during the imaging window, conducted 12 h and 24 h post‐injection, may further contribute to residual signal.

FTase‐mediated nanobody modification enabled precise installation of PEG moieties with defined sizes, which emerged as a critical design parameter for optimizing nanobody‐based PET imaging performance by mitigating pharmacokinetic limitations inherent to small protein tracers. Modulation of PEG size provided a tunable handle over nanobody‐based PET probe pharmacokinetics, allowing the balance between circulation time, renal clearance, and imaging contrast to be optimized for imaging time windows.

A direct head‐to‐head comparison will be required in future studies to rigorously evaluate FTase‐mediated nanobody‐PET probe generation relative to other site‐specific conjugation strategies, such as sortase‐mediated labeling. Nonetheless, qualitative comparison of the PET probes developed here with prior reports using sortase‐based approaches reveals broadly comparable in vivo imaging performance. Differences in pharmacokinetics and renal clearance may arise from the distinct physiochemical features of the isoprenoid group and linker architectures used to attach the DFO chelator and PEG moieties, which could contribute to observed variations between conjugation strategies.

Collectively, FTase‐mediated nanobody modification offers a compelling, reproducible, and straightforward platform for producing highly pure, chemically defined imaging probes in high yield, features that are particularly advantageous for clinical translation. Together, these findings position FTase‐engineered nanobody‐PET probes as a broadly applicable and adaptable imaging modality with strong potential to support future immune monitoring across diverse immune‐mediated diseases.

## Author Contributions

**Sneha Venkatachalapathy**: conceptualization, investigation, writing – original draft, writing – review and editing, formal analysis, data curation. **Yoon Ho Lee**: formal analysis, investigation. **Ali Salehi Farid**: investigation, formal analysis. **Heydar Moravej**: investigation, formal analysis. **Sina Djafari Rouhani**: investigation, formal analysis. **Jennifer E. Rowley**: investigation, formal analysis. **Mohammad Rashidian**: conceptualization, funding acquisition, writing – original draft, writing – review and editing, project administration, supervision, resources, formal analysis. **Mark D. Distefano**: conceptualization, funding acquisition, writing – original draft, writing – review and editing, formal analysis, project administration, supervision, resources.

## Conflicts of Interest

The authors declare no conflicts of interest.

## Supporting information



The complete description of the materials, methods, and additional data is provided in Supporting Information and Supplementary videos.**Supporting File 1**: anie74168‐sup‐0001‐SuppMat.pdf.

**Supporting File 2**: anie74168‐sup‐0002‐SuppVideos.zip.

## Data Availability

All data supporting the findings of this study are openly accessible through the Data Repository for the University of Minnesota (DRUM) at https://doi.org/10.13020/9qtz‐yg79.
