# Supplementary material for: Farnesyltransferase Enables Modular Assembly of Dual‐Functional Nanobody Conjugates for in Vivo Imaging of Inflammation
Source: Angew Chem Int Ed Engl. 2026 Aug 24;65(42):e5009703. doi: 10.1002/anie.5009703 (PMC13653577; doi:10.1002/anie.5009703)
Supplement: Supplementary file 1 [file ANIE-65-e5009703-s001.pdf]

Supporting Information

Farnesyltransferase Enables Modular Assembly of Dual-Functional Nanobody Conjugates for In Vivo Imaging of Inflammation.

Sneha Venkatachalapathy[1], Yoon Ho Lee[2], Ali Salehi Farid[2], Heydar Moravej[2], Sina Djafari Rouhani[2], Jennifer E. Rowley[2], Mohammad Rashidian[2,3,4]*, Mark D. Distefano[1]*

Affiliations:

[1] Department of Chemistry, University of Minnesota, Twin Cities, 207 Pleasant Street SE, Minneapolis, MN 55455, USA

[2] Department of Cancer Immunology and Virology, Dana-Farber Cancer Institute, Boston, MA 02115, USA.

[3] Parker Institute for Cancer Immunotherapy, San Francisco, CA, USA.

[4] Department of Radiology, Brigham and Women's Hospital, Harvard Medical School, Boston, MA, USA.

*Corresponding authors (mohammad_rashidian@dfci.harvard.edu, diste001@umn.edu)

Table of Contents

List of chemical structures with numbers and acronyms S5

Abbreviations S9

Figure S1. Nanobody-CVIA refolding optimization.. S10

Figure S1 continued: SDS-PAGE analysis of **N11b**, **N45**, **N11bp** and **N45p**. S11

Figure S2: Flow cytometry affinity comparison of refolded and periplasmically expressed anti-CD11b and anti-CD45 nanobodies S12

Figure S3: LC-ESI-MS analysis of **N11b** conjugates generated using compound **1**.......... S13

Figure S4: Flow cytometry affinity titration of **N11b-TAM** for CD11b................................ S14

Figure S5: Confocal imaging of **N11b-TAM** binding on RAW 264.7 cells S15

Figure S6: SDS-PAGE analysis and SEC purification of **N11b-DFO** S16

Figure S7: PET imaging of healthy following administration of **^{89}Zr-N11b-DFO** S17

Figure S8: In vivo organ-level biodistribution of **^{89}Zr-N11b-DFO** S18

Figure S9: SDS PAGE and LC-ESI-MS analysis of **N11b-AzAl** S19

Figure S10: SDS-PAGE analysis of dual labeling at pH 6.0 with pPDA S20

Figure S11: SDS-PAGE analysis of dual labeling with amine catalysts at pH 6.0 .... S21

Figure S12: SDS-PAGE analysis of dual labeling with amine catalysts at pH 5.5 and 6 ... S22

Figure S13: SDS-PAGE analysis of dual labeling on **N11b-Az-TCO**................................ S23

Figure S14: One-pot FTase-catalyzed dual labeling of **N11b** and **N45** with PEG 20 kDa and TAMRA.. S24

Figure S15: SDS-PAGE analysis and SEC purification of **N11b-PEG5-DFO** and **N11b-PEG10-DFO** S25

Figure S16: SDS-PAGE analysis of anti-FLAG affinity purification of **N11b-AzAl** and **N45-AzAl** conjugates.. S26

Figure S17: SEC chromatograms (280 nm) of mPEG-tetrazine reagents (5 and 10 kDa).S27

Figure S18: PET imaging of healthy mice after injection of **^{89}Zr-N11b-PEG10-DFO**......... S28

Figure S19: Quantitative comparison of the paw SUV_{mean} in PET imaging using **^{89}Zr-N11b-PEG10-DFO**.. S29

Figure S20: PET imaging of healthy mice post injection with **^{89}Zr-N11b-PEG5-DFO**........ S30

Figure S21: Maleimide-thiol based PEGylation of the CVIA cysteine of **N11b** S31

Figure S22: LC-ESI-MS analysis of N45 and N45-AzAl S32

Figure S23: SDS-PAGE analysis of N45-PEG10-TAM S32

Figure S24: SEC purification of **N45-PEG10-DFO** and SDS PAGE analysis S33

Figure S25: PET imaging of CFA-treated mice post injection with **^{89}Zr-N45-PEG10-DFO** with CD45 pre-blocking S34

Figure S26: Comparison of organ-wise SUV_{mean} values across treatment groups for PET imaging using **^{89}Zr-N45-PEG10-DFO** at 12 h S35

Figure S27: Side-by-side PET imaging comparison assessing the influence of PEGylation and target-specific binding in PEGylated nanobodies S36

Figure S28: Radio-thin-layer-chromatogrphy of ^{89}Zr labeled N11b conjugates S37

Uncropped gels S38

Experimental procedures

General S42
Docking S43
Design and expression of the anti-murine CD11b and CD45 nanobodies (N11b and N45)
I. Expression of N11b and N45 .... S44
II. Isolation of N11b and N45 from inclusion bodies S44
III. Refolding of purified N11b and N45 .... S45
Nanobody refolding optimization and functional validation by flow cytometry S46
Design and periplasmic expression of the anti-mouse CD11b and CD45 nanobodies (N11bp and N45p) S47
Head-to-head comparison of binding affinities of purified periplasmically expressed nanobodies (N11bp and N45p) and purified refolded nanobodies (N11b and N45) S47
In vitro farnesyltransferase-catalyzed nanobody labeling S48
Metal-free click reaction between azide functionalized nanobody with TAMRA-DBCO or DFO-DBCO S48
One-pot simultaneous coupling of nanobodies with TAMRA-DBCO or DFO-DBCO and aminooxy-PEG (5, 10, or 20 kDa) S48
Sequential labeling of nanobodies with TAMRA-DBCO or DFO-DBCO followed by aminooxy-TCO S49
Tetrazine ligation of TCO-modified intermediates with methyltetrazine-mPEG S49
One-pot simultaneous coupling of nanobodies with aminooxy-TAMRA and DBCO-mPEG (20 kDa) S49
Flow cytometry-based affinity titration of TAMRA-labeled nanobodies S49
Affinity titration data analysis S50
Anti-FLAG affinity purification of nanobodies S50
Size-exclusion chromatographic purification of labeled nanobodies S50
Confocal imaging of N11b-TAM binding to CD11b-expressing RAW 264.7 cells S51

Confocal imaging of RAW 264.7 cells showing differential binding of N11b-PEG10-TAM and N45-PEG10-TAM S51
Maleimide-thiol Michael addition reaction S51
PET/CT imaging experiments S51
Image quantification and organ PET mean SUV_{mean} analysis S52
RadioTLC S52

Procedures for the synthesis of compounds 1 and 2 ..... S53

Spectral data for synthetic compounds

Compound **2** ^{1}H NMR S58
Compound **2** ^{13}C NMR S59
Compound **2** ^{31}P NMR S60
Compound **2** HRMS S60
Compound **6** ^{1}H NMR S61
Compound **6** ^{13}C NMR S62
Compound **6** HRMS S62
Compound **7** ^{1}H NMR S63
Compound **7** ^{13}C NMR S64
Compound **7** HRMS S64
Compound **8** ^{1}H NMR S65
Compound **8** ^{13}C NMR S66
Compound **8** HRMS S66

References S67

List of chemical structures and protein conjugates with numbers and acronyms

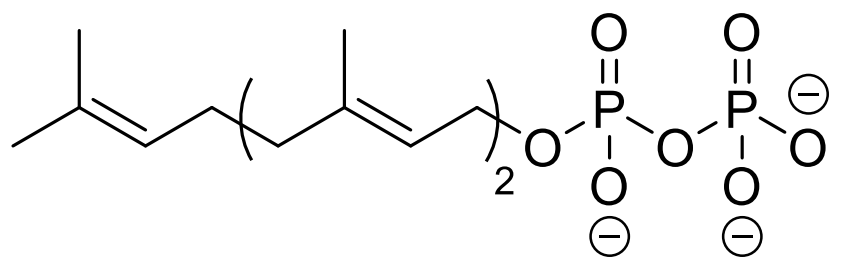

FPP

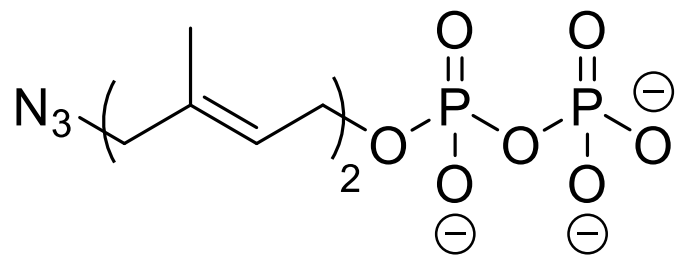

1

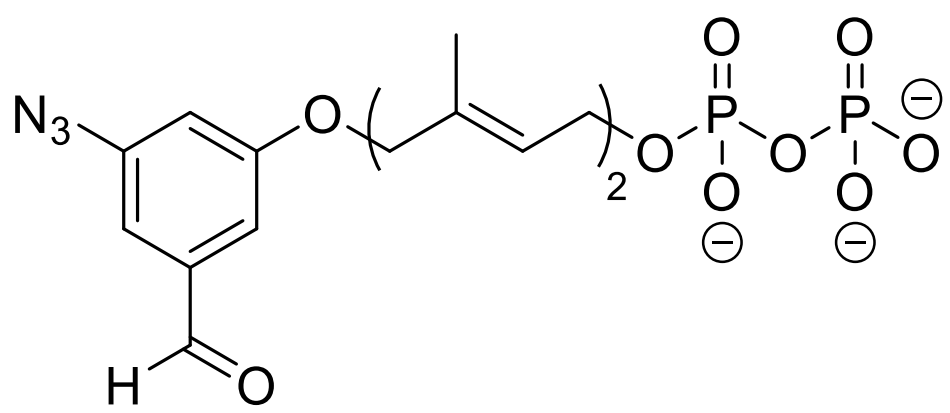

2

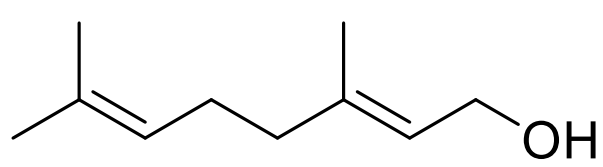

Geraniol

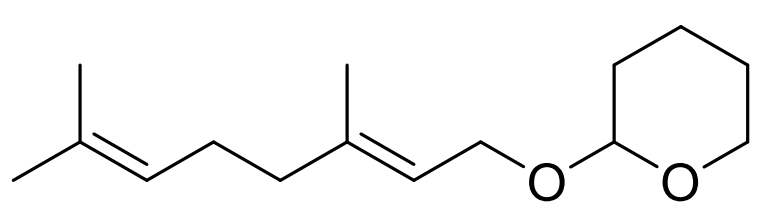

3

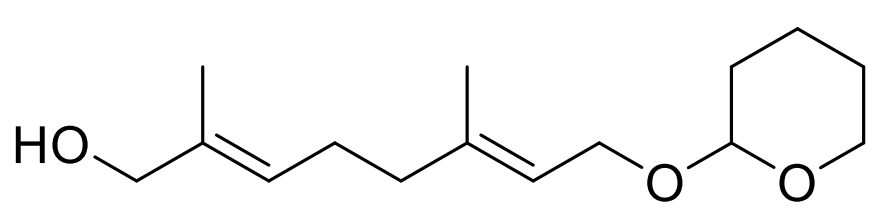

4

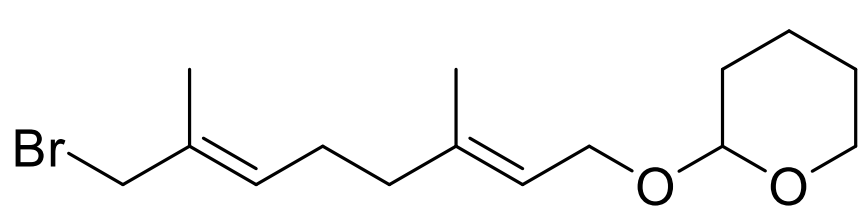

5

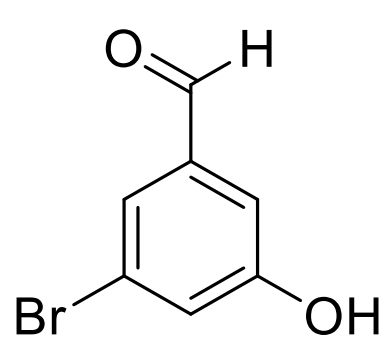

3-Bromo-5-hydroxybenzaldehyde

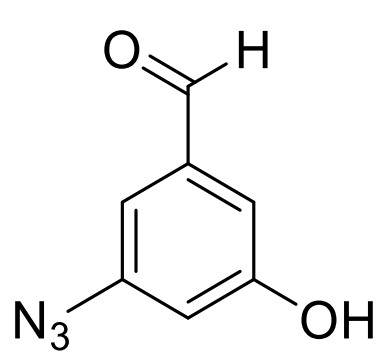

6

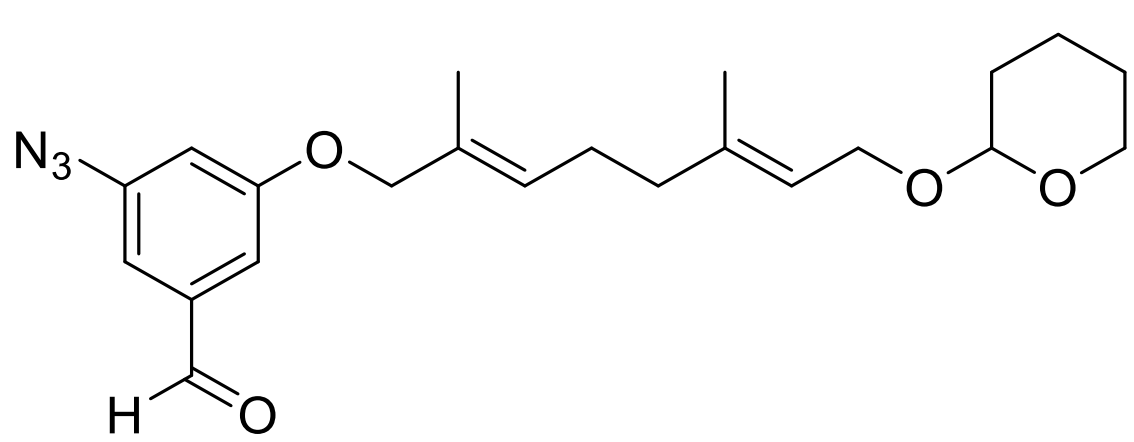

7

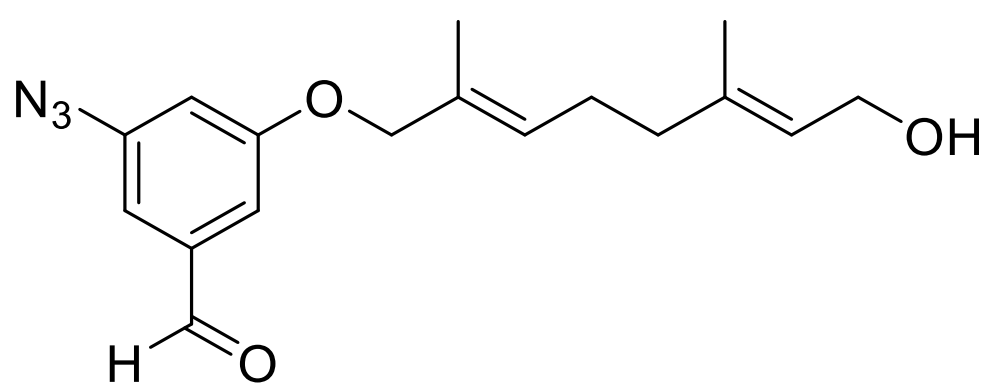

8

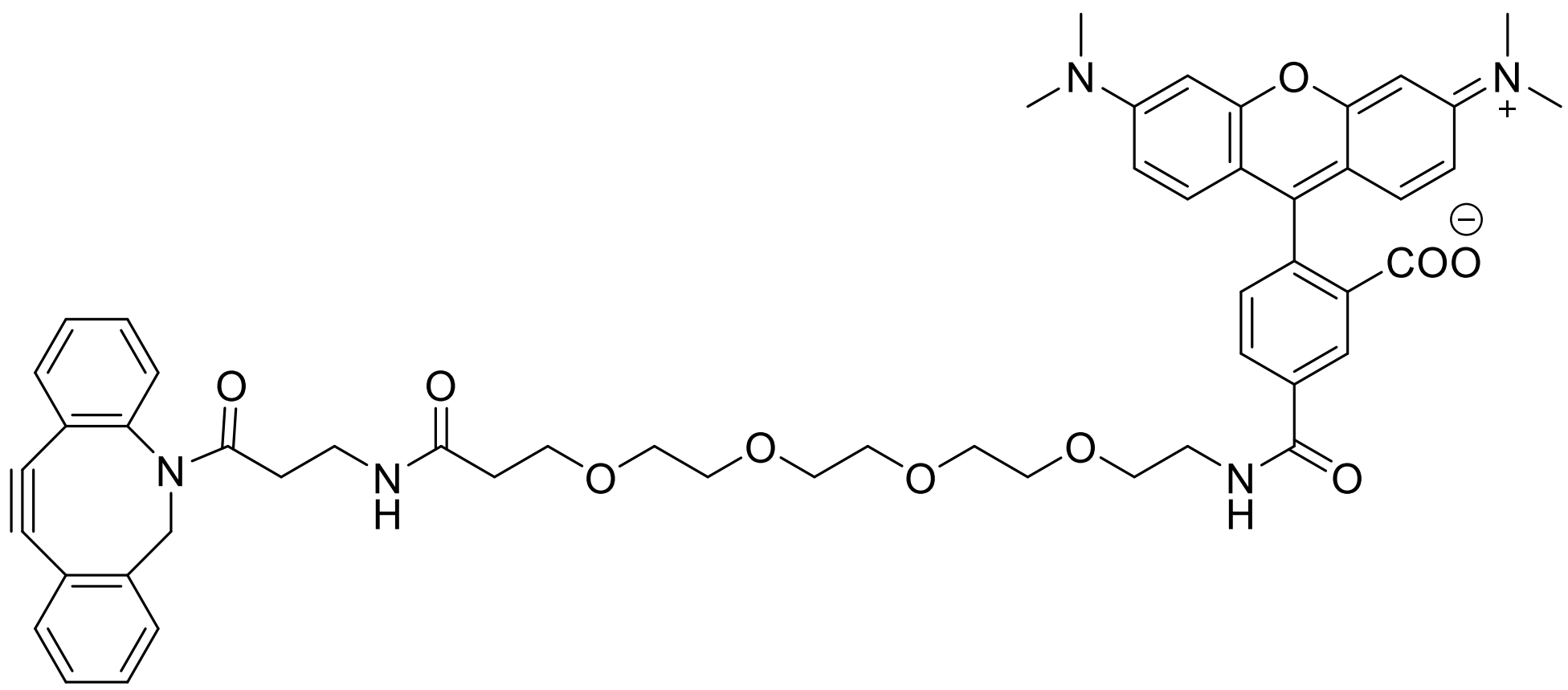

TAMRA-DBCO

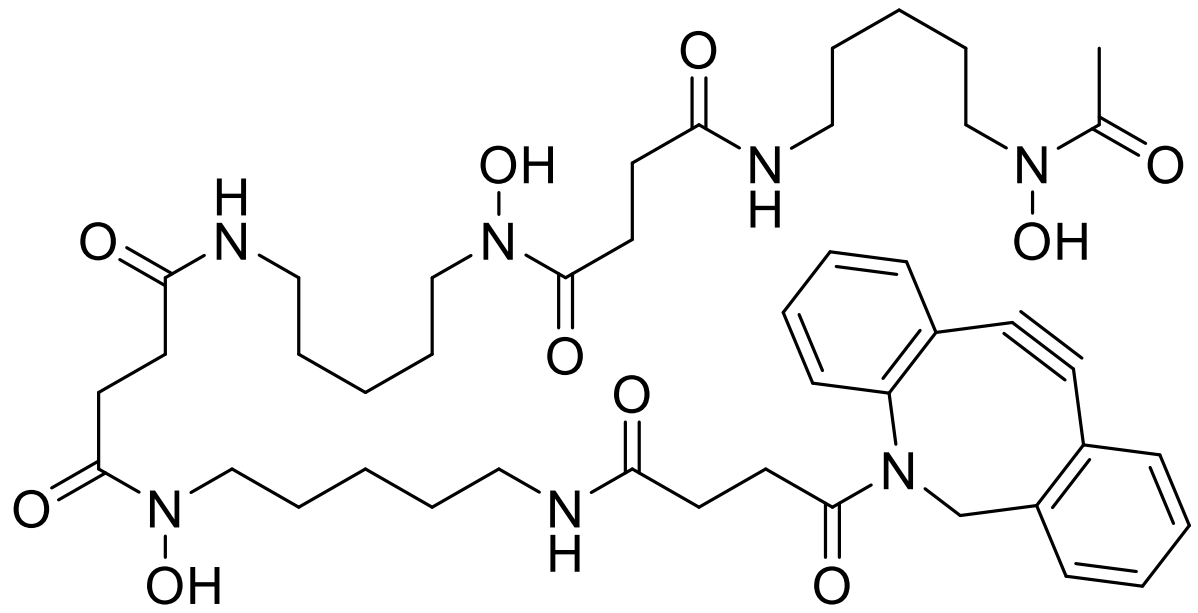

DFO-DBCO

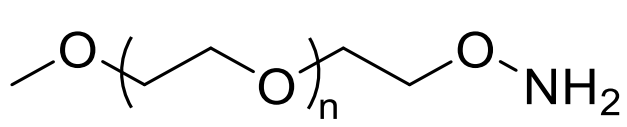

Aminooxy-mPEG

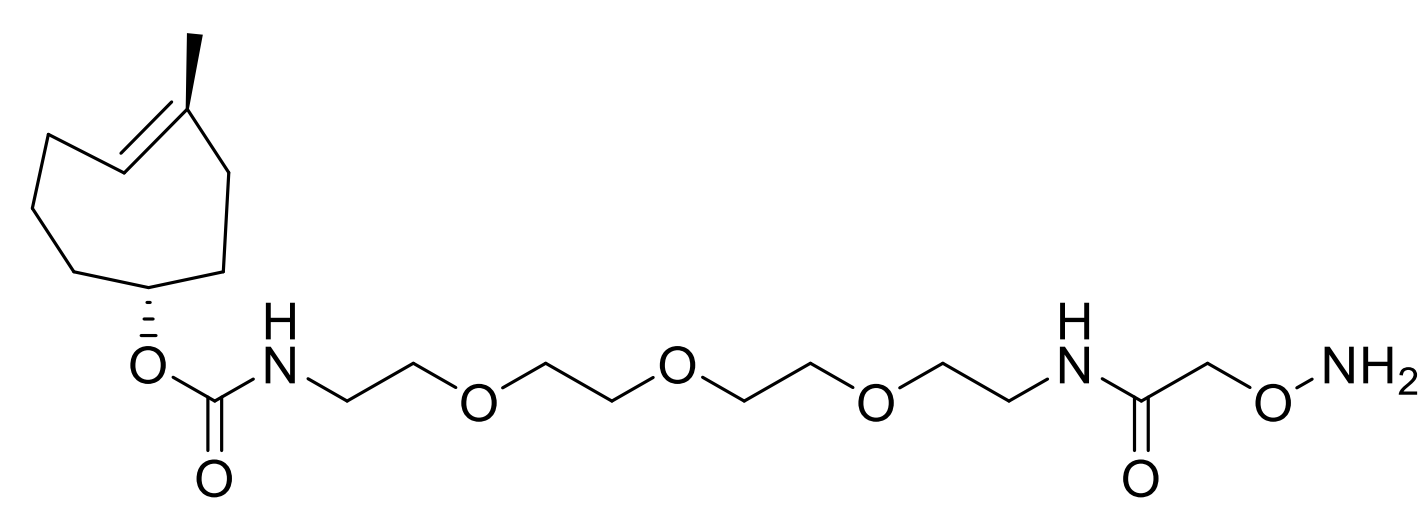

Aminooxy-TCO

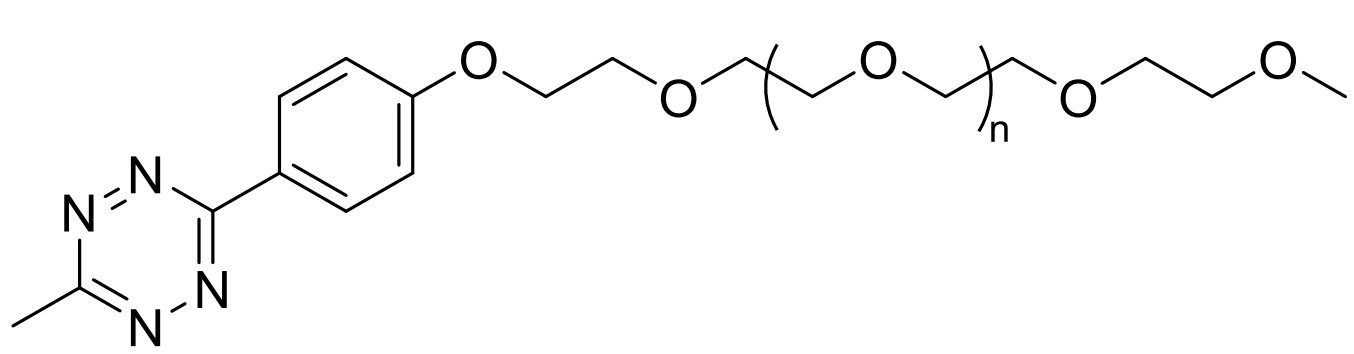

Methyltetrazine-mPEG

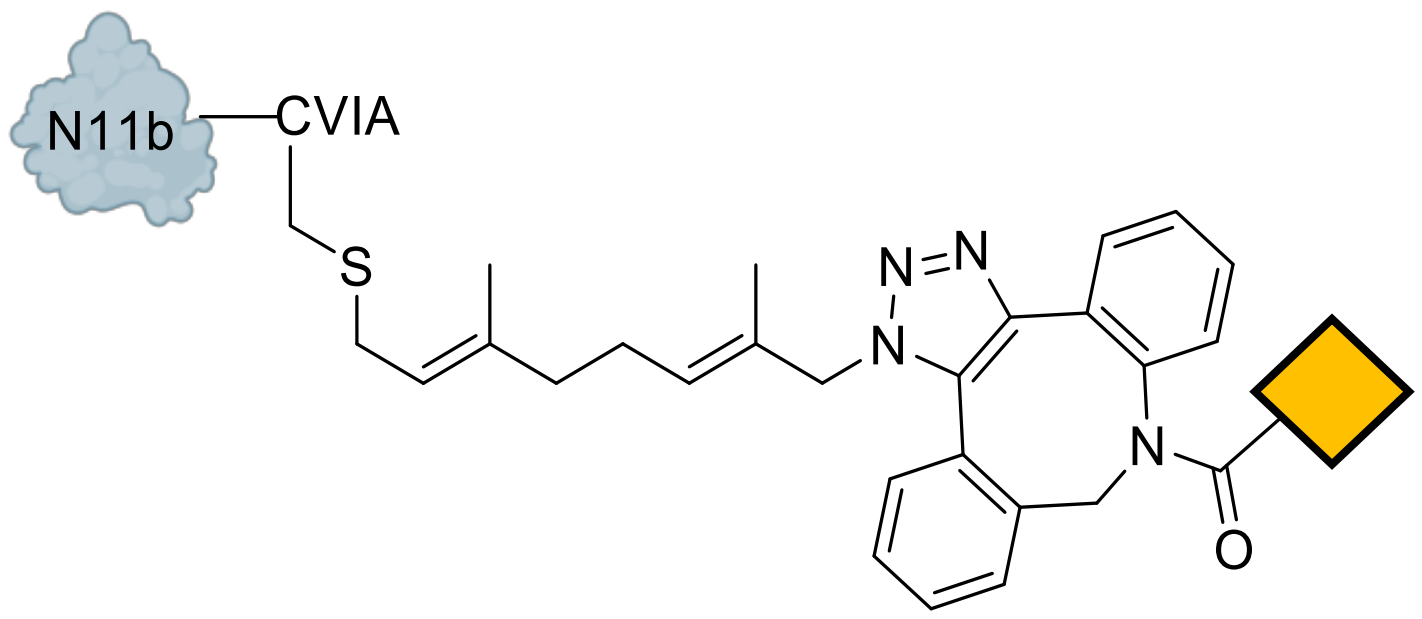

N11b-TAM or N11b-DFO

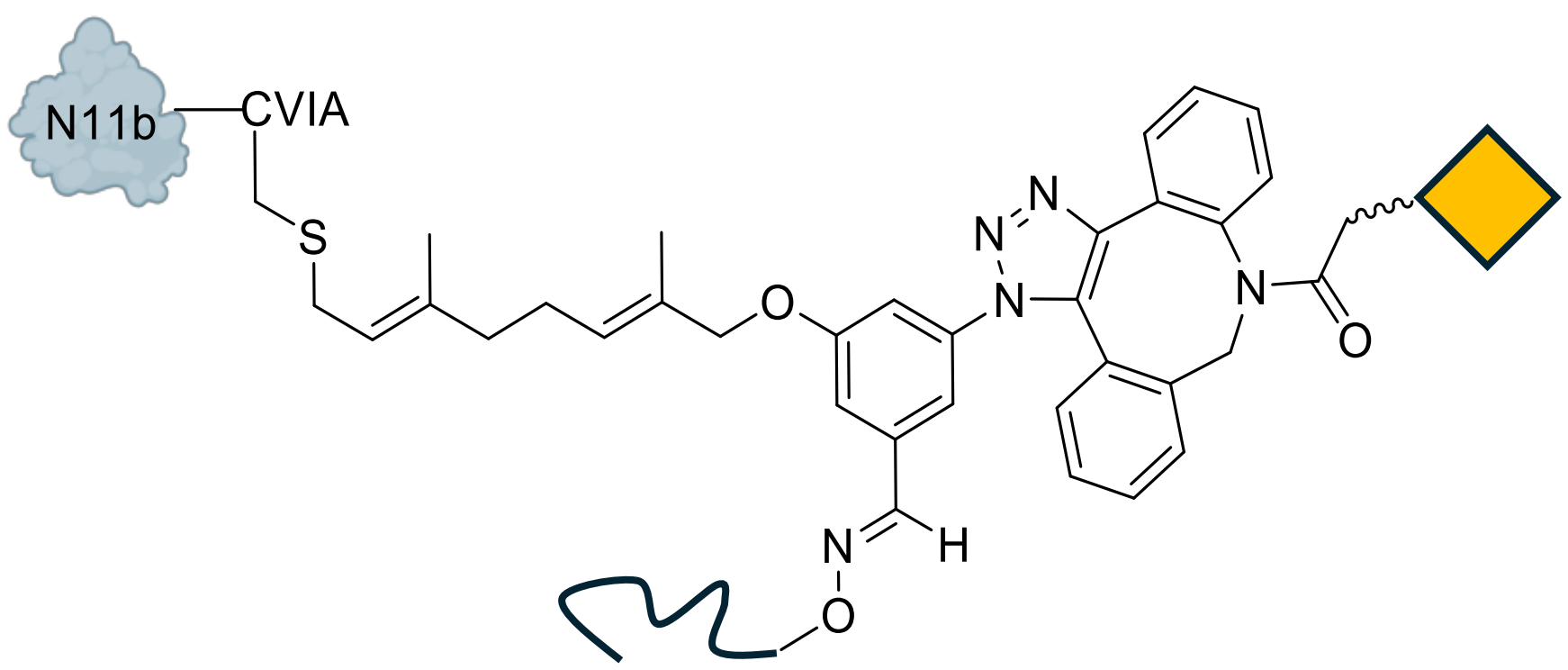

N11b-PEG5-TAM

or

N11b-PEG5-DFO

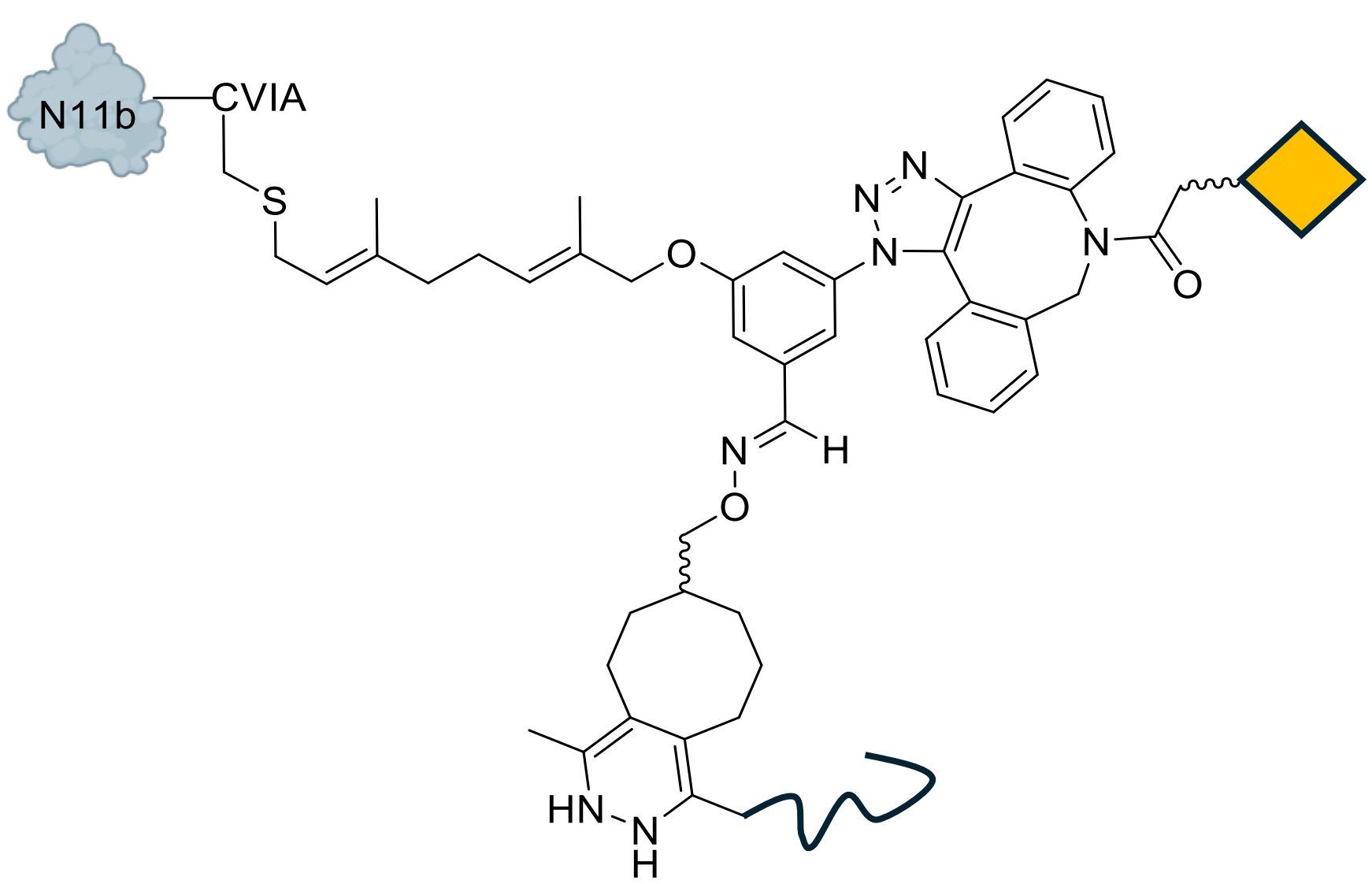

N11b-PEG10-TAMRA

Or

N11b-PEG10-DFO

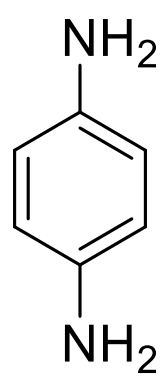

p-PDA (para-phenylene diamine)

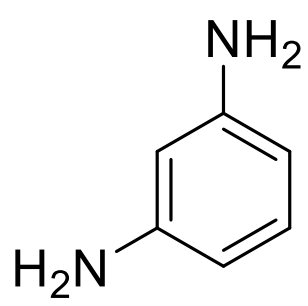

m-PDA (meta-phenylene diamine)

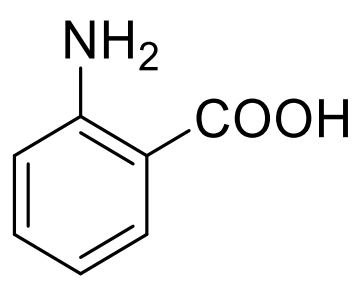

Anthranilic acid

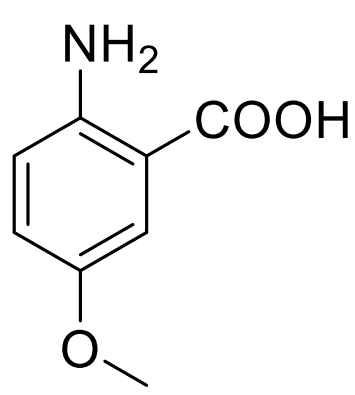

5-Methoxy anthranilic acid

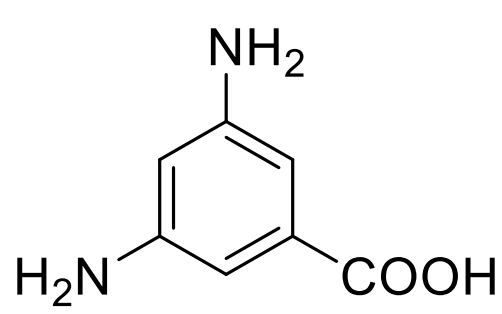

3,5-Diaminobenzoic acid

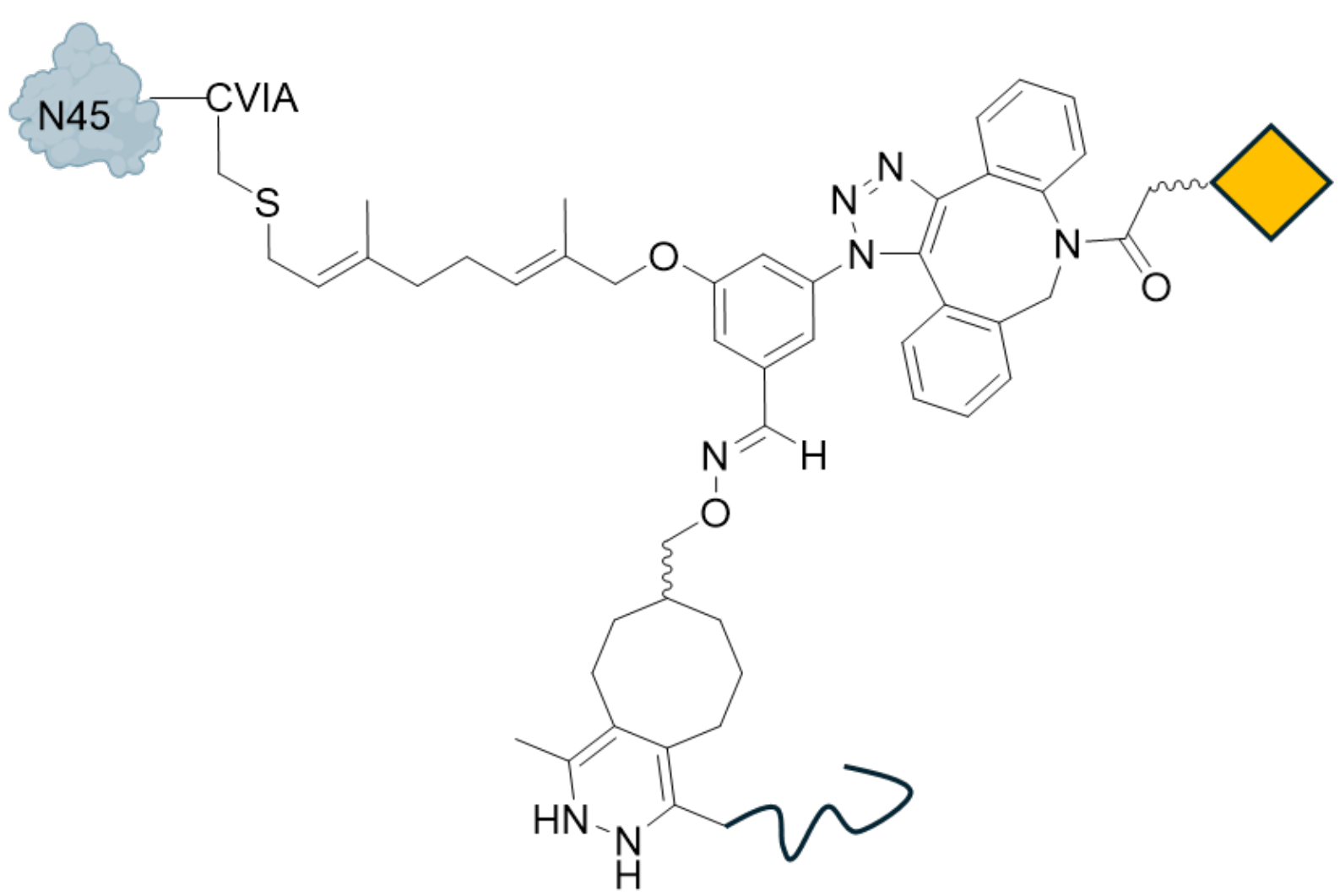

N45-PEG10-TAMRA
or
N45-PEG10-DFO

Abbreviations

FarnesylTransferase (FTase)

Computed Tomography (CT)

Positron Emission Tomography (PET)

^{18}F-fluorodeoxyglucose (^{18}F-FDG)

Variable Heavy domain of Heavy chain (VHH/nanobodies)

Microbial Transglutaminase (mTG)

Sortase A (SrtA)

Farnesyl diphosphate (FPP)

Deferoxamine (DFO)

Tetramethylrhodamine (TAMRA)

Strain Promoted Azide-Alkyne Click reaction (SPAAC)

Complete Freund's Adjuvant (CFA)

Standardized Uptake Value (SUV)

Maximal Intensity Projections (MIP)

Radio-thin-layer-chromatography (RadioTLC)

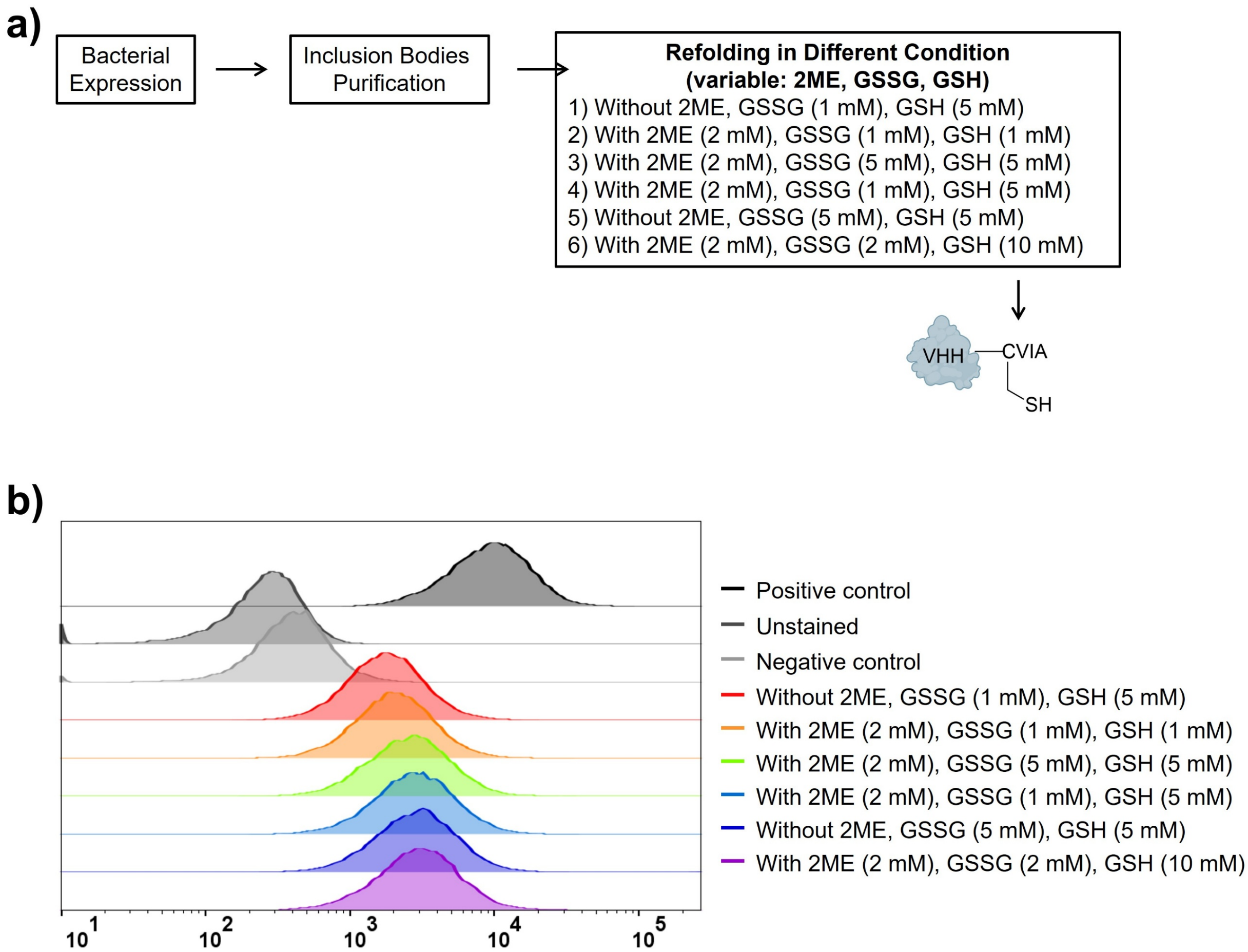

Figure S1. Nanobody refolding optimization and functional validation by flow cytometry. a) Refolding optimization workflow. Nanobody was expressed in E. coli, isolated as inclusion bodies, and subjected to oxidative refolding under varying redox and buffer conditions to identify conditions yielding soluble, functional protein. b) Initial functional validation by flow cytometry. RAW 264.7 cells were incubated with ~100 nM crude refolded nanobody (prior to purification) in refolding buffer, washed, and stained with anti-His APC antibody to assess binding via APC fluorescence. As a positive control, cells were stained with 100 nM periplasmically expressed purified anti-CD11b nanobody (**N11bp**) lacking the CVIA tag, followed by anti-His APC antibody staining. As a negative control, cells were incubated with anti-His APC alone in the absence of nanobody.

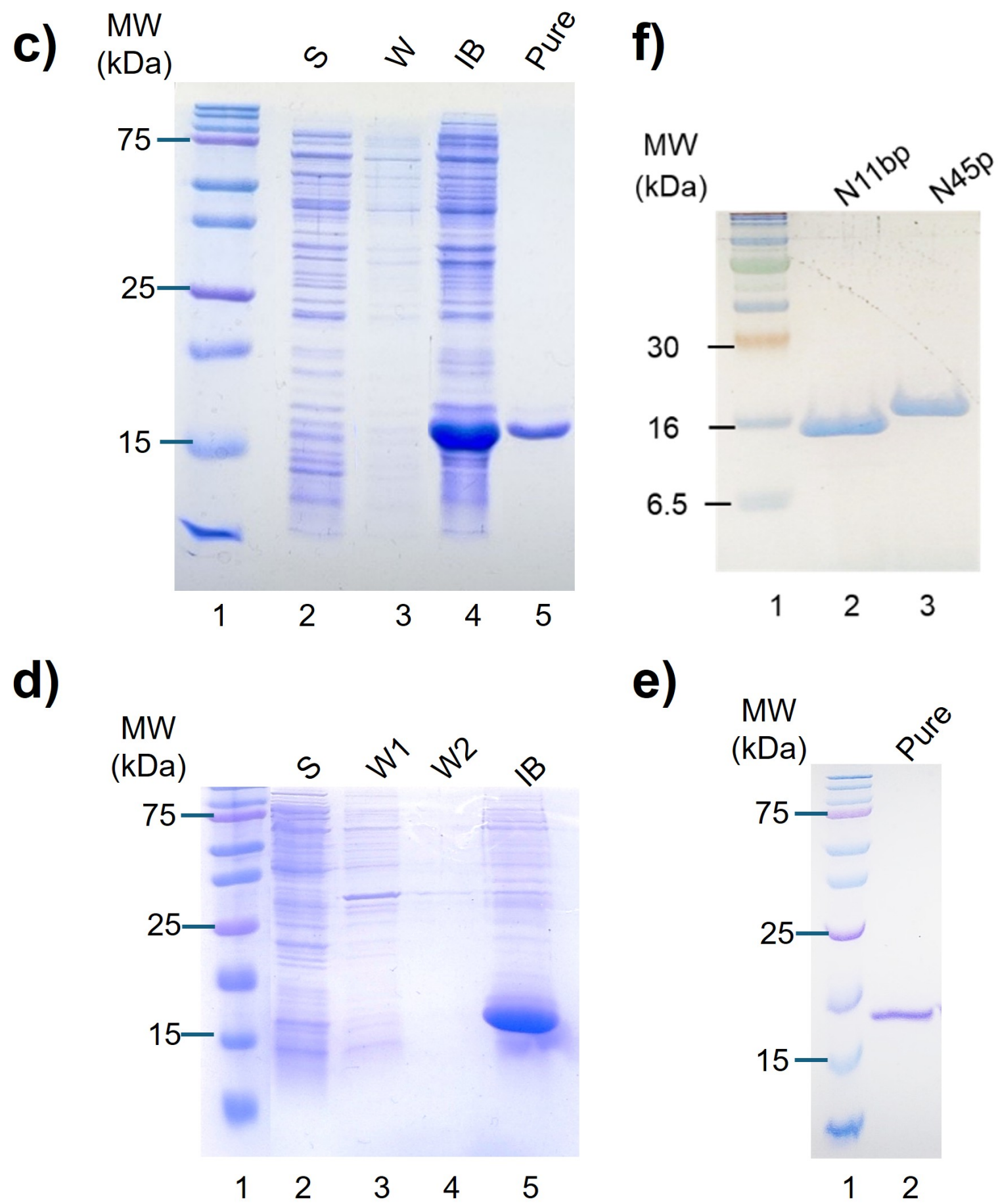

Figure S1 continued: SDS-PAGE analysis of anti-CD11b- and anti-CD45-targeting refolded nanobodies **N11b** and **N45** and periplasmically expressed **N11bp** and **N45p**. Each construct carries a C-terminal CVIA motif to enable recognition by FTase. c) Characterization of **N11b** following refolding from inclusion bodies and subsequent purification. Lane 1: protein ladder; Lane 2: soluble fraction after lysis (S); Lane 3: wash of the insoluble fraction (W); Lane 4: inclusion bodies after solubilization in 8 M urea (IB); Lane 5: purified and refolded **N11b** bearing the CVIA tag (Pure). d) Characterization of the **N45** following refolding from inclusion bodies and subsequent purification. Lane 1: protein ladder; Lane 2: soluble fraction (S); Lane 3 and 4: washes of the insoluble fraction (W1 and W2); Lane 5: inclusion bodies after solubilization in 8 M urea (IB); e) Lane 1: protein ladder; Lane 2: purified and refolded N45 bearing the CVIA tag. In gel d, there was an empty lane between lane 1 and lane 2 which was removed and the lanes 1 and 2 are spliced together. f) Lane 1: protein ladder; Lane 2: purified **N11bp**; Lane 3: purified **N45p**. Uncropped gel provided in uncropped gels section.

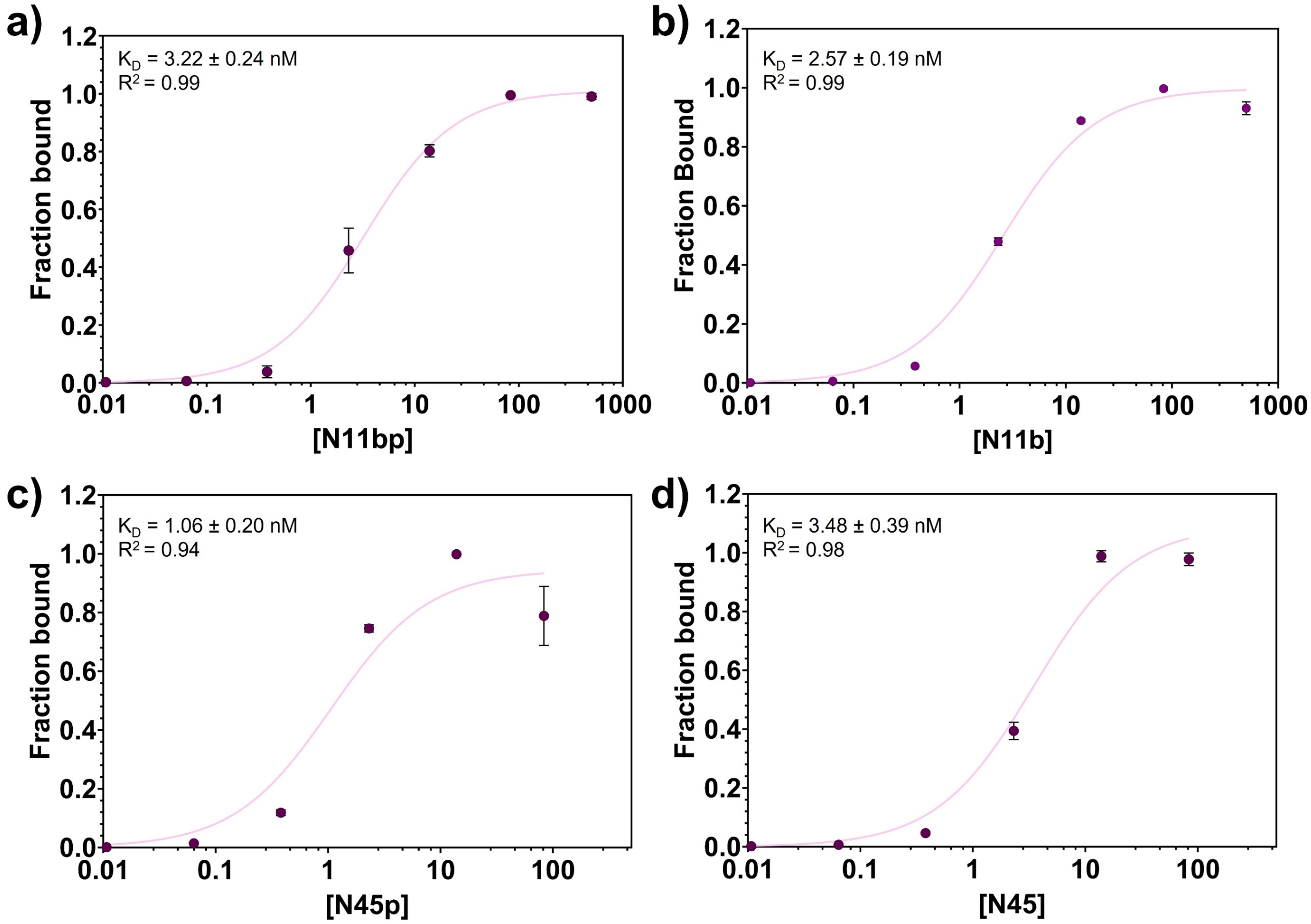

Figure S2. Head-to-head comparison of flow cytometry-based affinity titrations of refolded and periplasmically expressed nanobodies targeting CD11b and CD45 on RAW 264.7 cells. Cells were plated in a 96-well plate (three columns per construct, eight concentrations across each row), incubated with increasing concentrations of nanobodies, washed, and stained with anti-FLAG PE (for FLAG-tagged constructs) or anti-His AF647 (for N11bp, which lacks a FLAG tag), followed by flow cytometric analysis (n = 3, triplicates). Data are reported as the fraction of $CD11b^+$ or $CD45^+$ receptors bound across 10,000 cells. Dissociation constants (K_D) were determined as follows: (a) **N11bp**, 3.22 ± 0.24 nM; (b) **N11b**, 2.57 ± 0.19 nM; (c) **N45p**, 1.06 ± 0.20 nM; and (d) **N45**, 3.48 ± 0.39 nM. Error bars represent standard deviation, s.d.

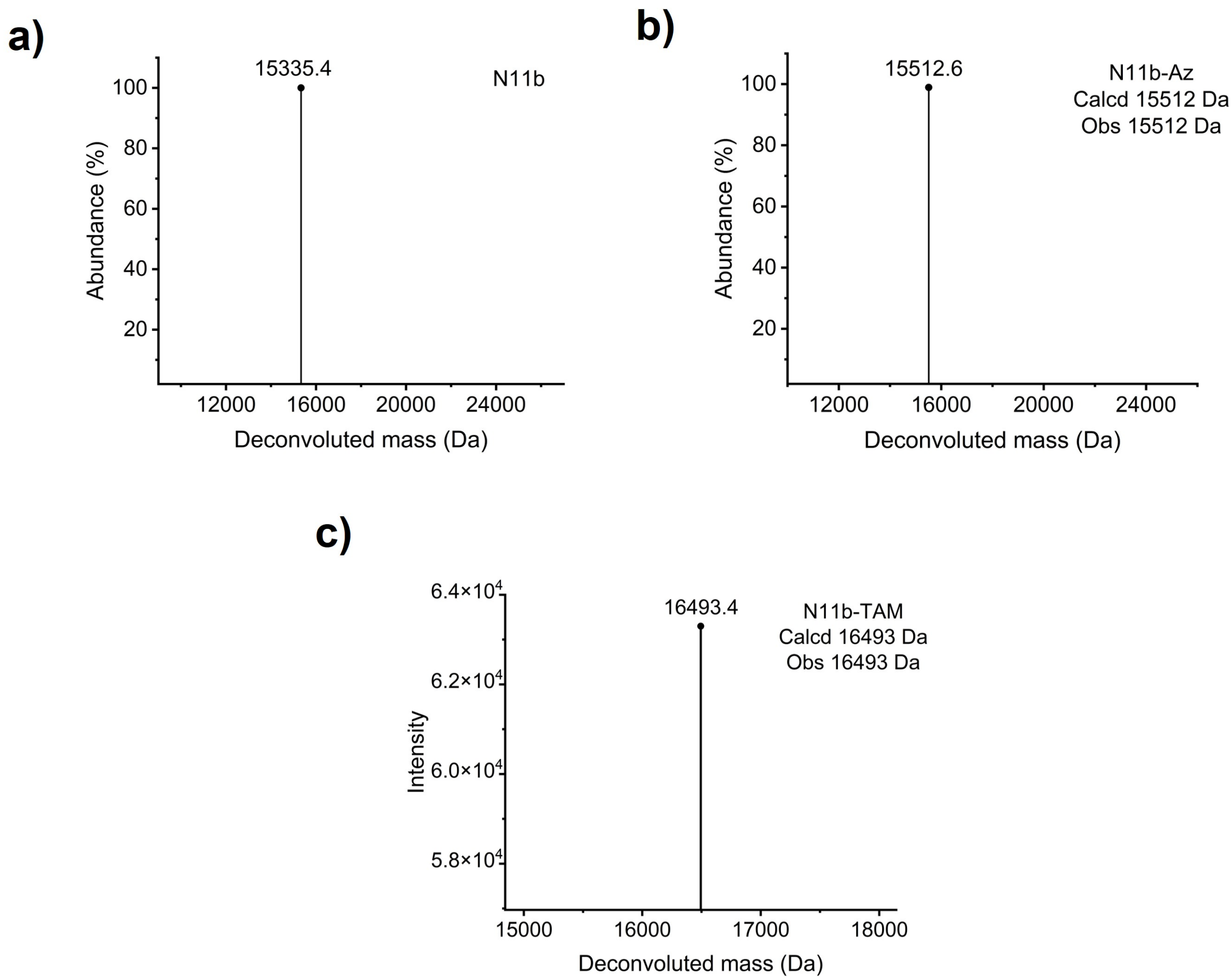

Figure S3: LC-ESI-MS analysis of **N11b** conjugates generated using compound **1**. a) Deconvoluted mass spectrum of the **N11b**. b) Deconvoluted mass spectrum of **N11b-Az** obtained after prenylation with bifunctional FPP analog **1**. Calc mass: 15512, Obs mass: 15512. c) Deconvoluted mass spectrum of **N11b-TAM** obtained after SPAAC reaction between **N11b-Az** and TAMRA-DBCO. Calc: 16493, Obs: 16493.

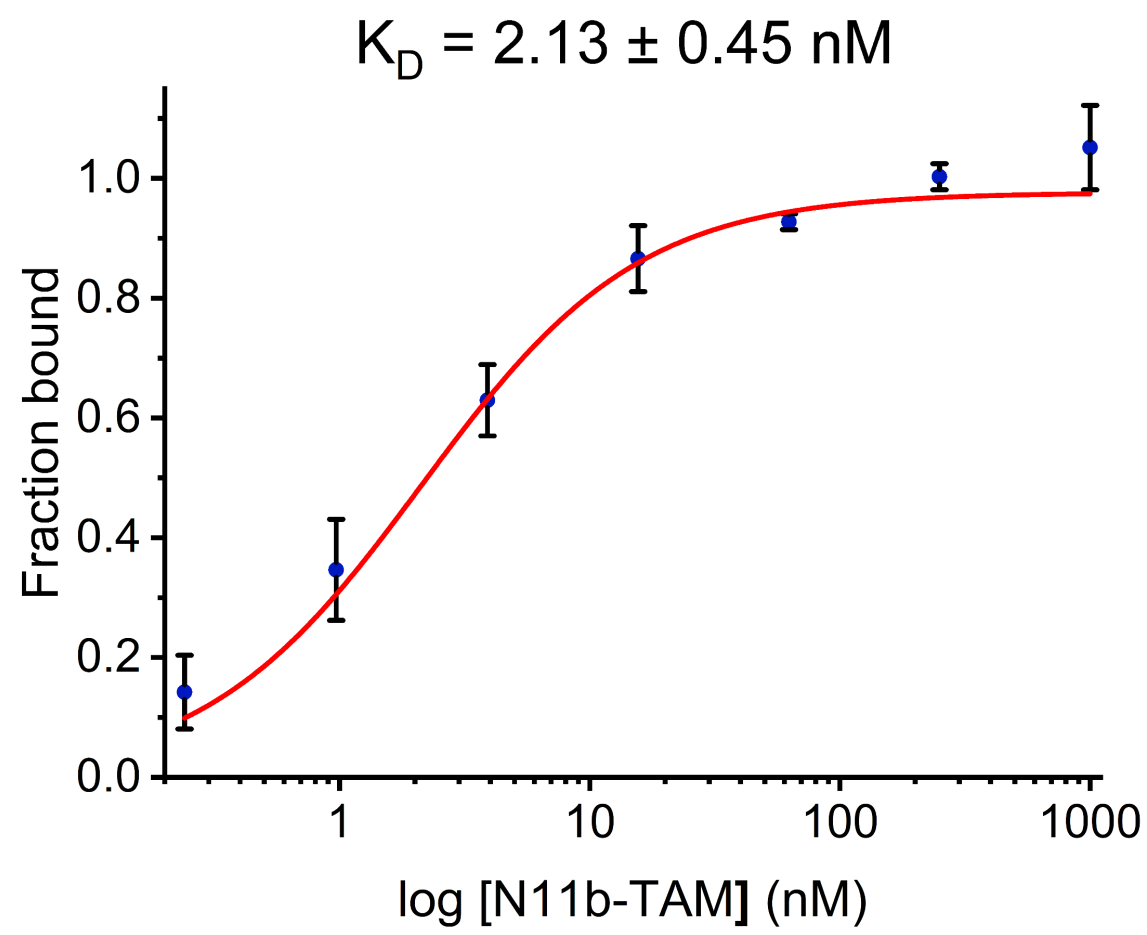

Figure S4: Flow cytometry-based affinity titration of **N11b-TAM** for CD11b. RAW 264.7 cells were treated with various concentrations of **N11b-TAM** followed by washing and flow cytometric analysis (n = 3 independent replicates), with results expressed as the fraction of CD11b receptors bound among 10,000 cells. A K_D value of 2.13 ± 0.45 nM was determined from this analysis. Error bars indicate s.d.

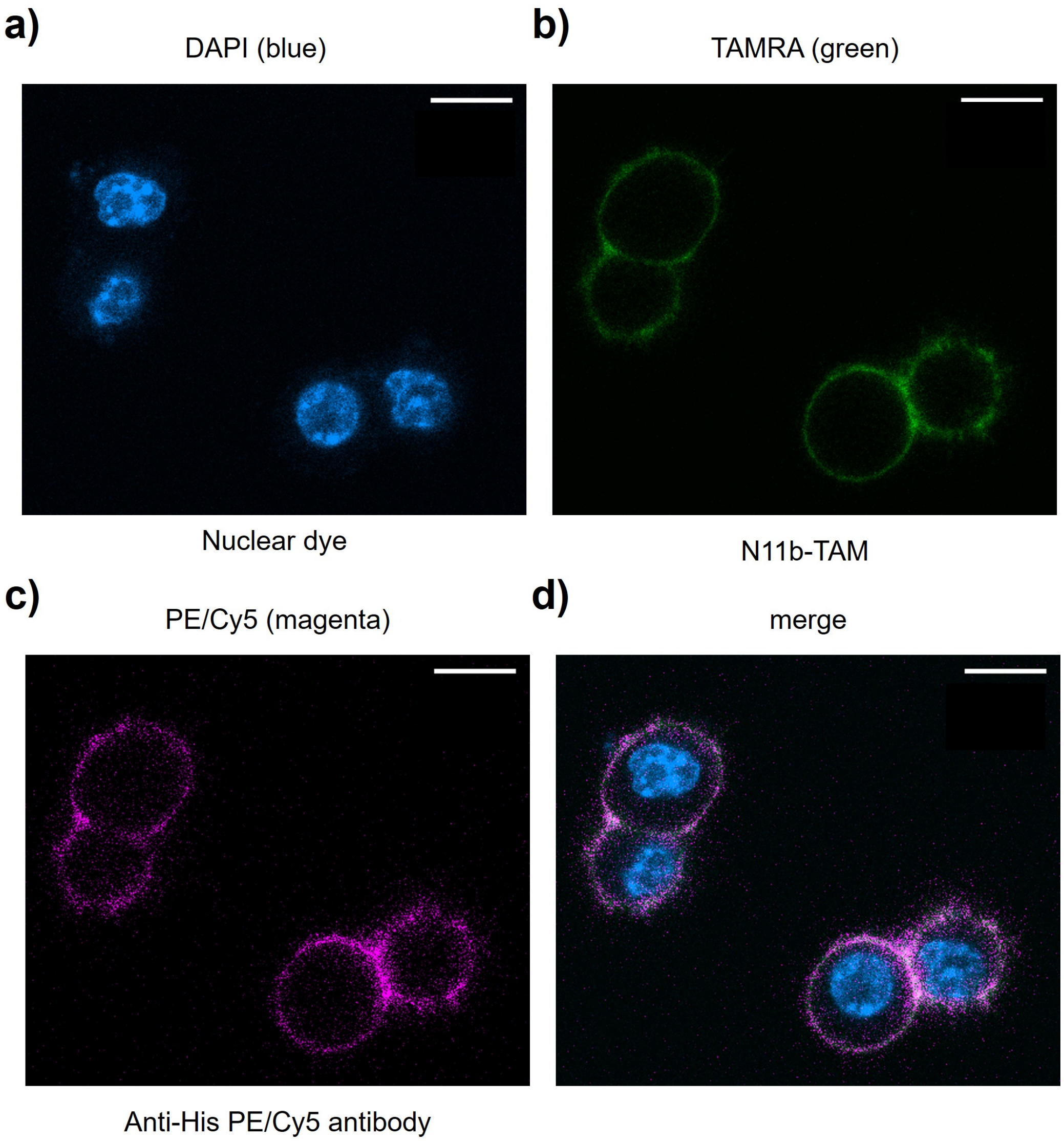

Figure S5. Confocal imaging of **N11b-TAM** binding to RAW 264.7 cells. Cells were incubated with 150 nM **N11b-TAM**, washed and subsequently subjected to immunostaining with an anti-His PE/Cy5 antibody to detect the N-terminal His-tag on **N11b-TAM**, followed by nuclear counterstaining with Hoechst 34580. Shown are a) Nuclei (blue), b) TAMRA fluorescence (green), c) Anti-His-PE/Cy5 fluorescence (magenta), and d) Merged image indicating colocalization at the membrane (light pink). Scale bar = 10 μm.

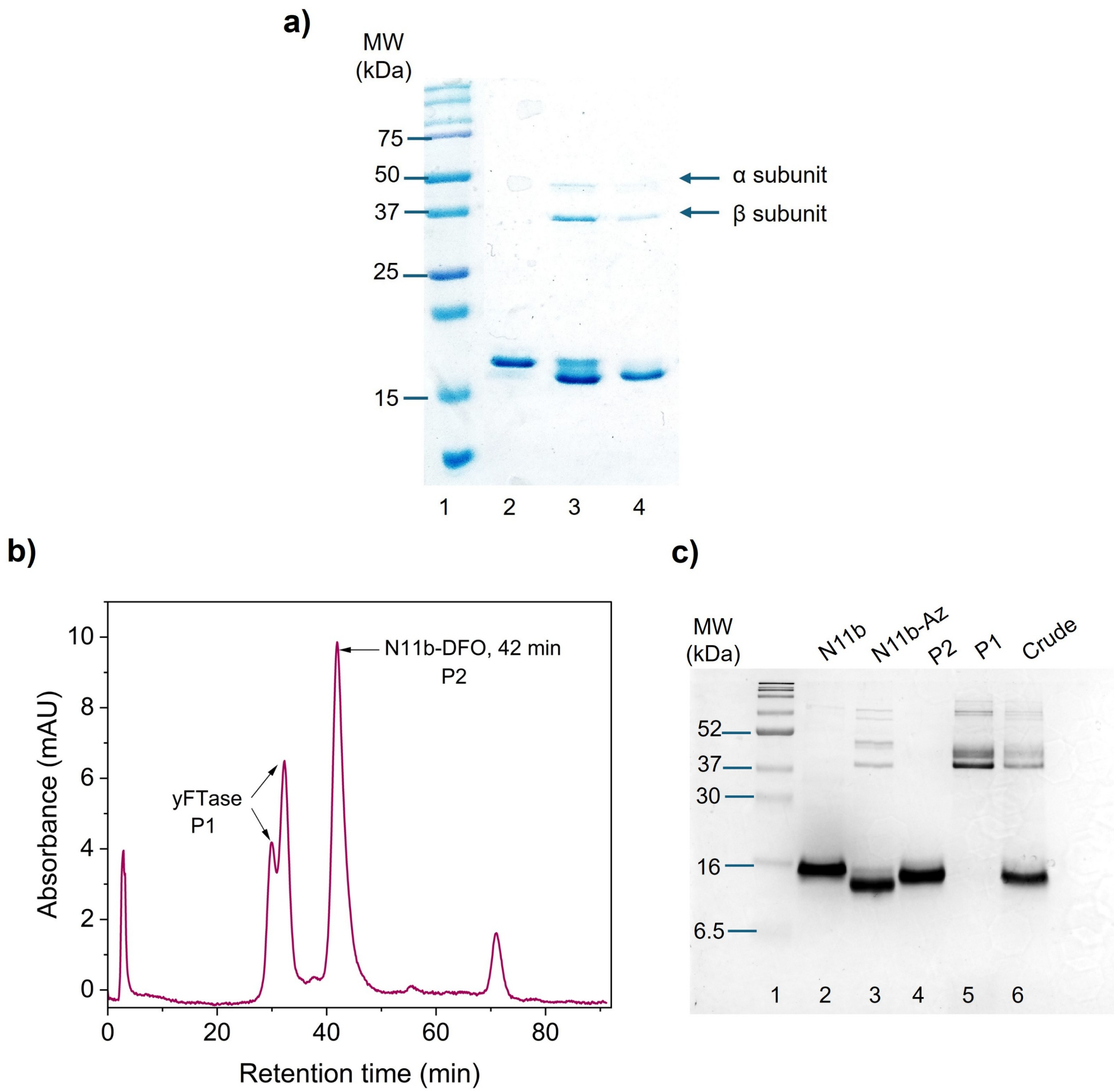

Figure S6: SDS-PAGE analysis and SEC purification of **N11b-DFO**. **N11b-DFO** was generated via a SPAAC reaction between **N11b-Az** and DFO-DBCO. a) SDS PAGE abalysis of **N11b-DFO** used for PET imaging. Lane 1: protein ladder; Lane 2: unmodified **N11b**; Lane 3: **N11b-Az**; Lane 4: **N11b-DFO**. The bands in between 37-50 kDa region are the α and β subunits of yeast FTase used for enzymatic modification. There was an empty lane between lane 1 and lane 2 which was removed and lanes 1 and 2 are spliced together. Uncropped gel provided in uncropped gels section. b) SEC chromatogram (280 nm). Peak P1 (29.9-32 min) corresponds to FTase, and peak P2 (42 min) corresponds to **N11b-DFO**. c) SDS-PAGE analysis of SEC fractions. Lane 1: protein ladder; Lane 2: unmodified **N11b**; Lane 3: **N11b-Az**; Lane 4: P2 fraction containing purified **N11b-DFO**; Lane 5: P1 fraction containing FTase; Lane 6: crude **N11b-DFO** sample prior to SEC purification.

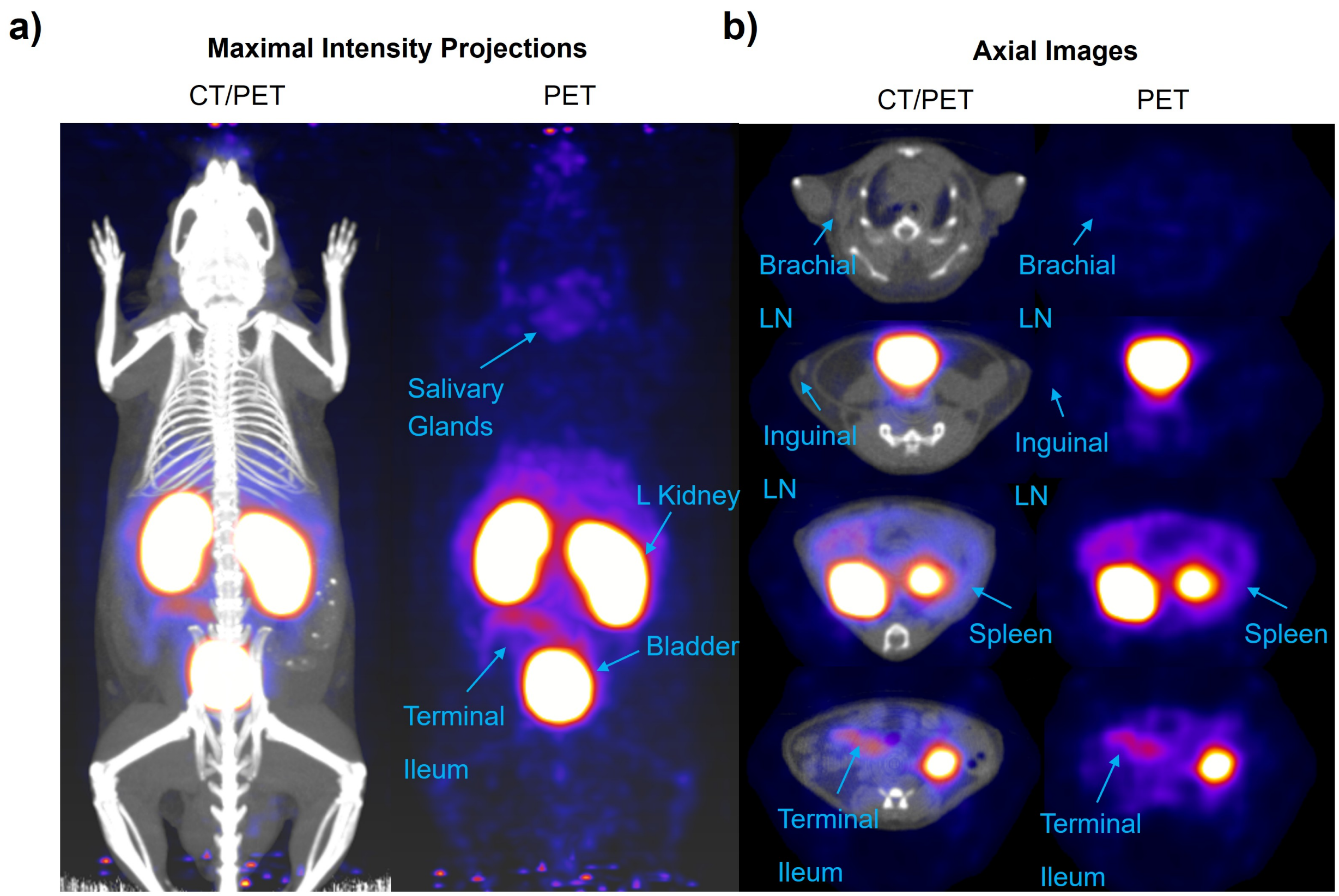

Figure S7: PET imaging of healthy following administration of **^{89}Zr-N11b-DFO**. The healthy C57BL/6 mice (n=4) were intravenously injected with around 50 μCi of the **^{89}Zr-N11b-DFO** probe and imaging was performed 1-2 hours post-injection. a) Representative maximum-intensity projection (MIP) images of CT/PET (left) and PET alone (right) b) Representative axial images of CT/PET (left) and PET alone (right). L stands for left; LN stands for lymph node.

a)

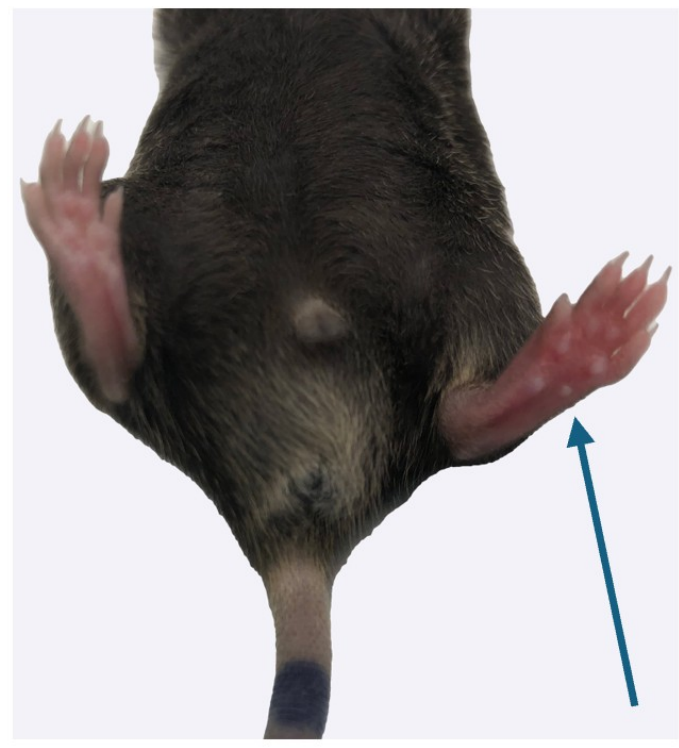

Right Paw (Control) Left Paw (+CFA)

b)

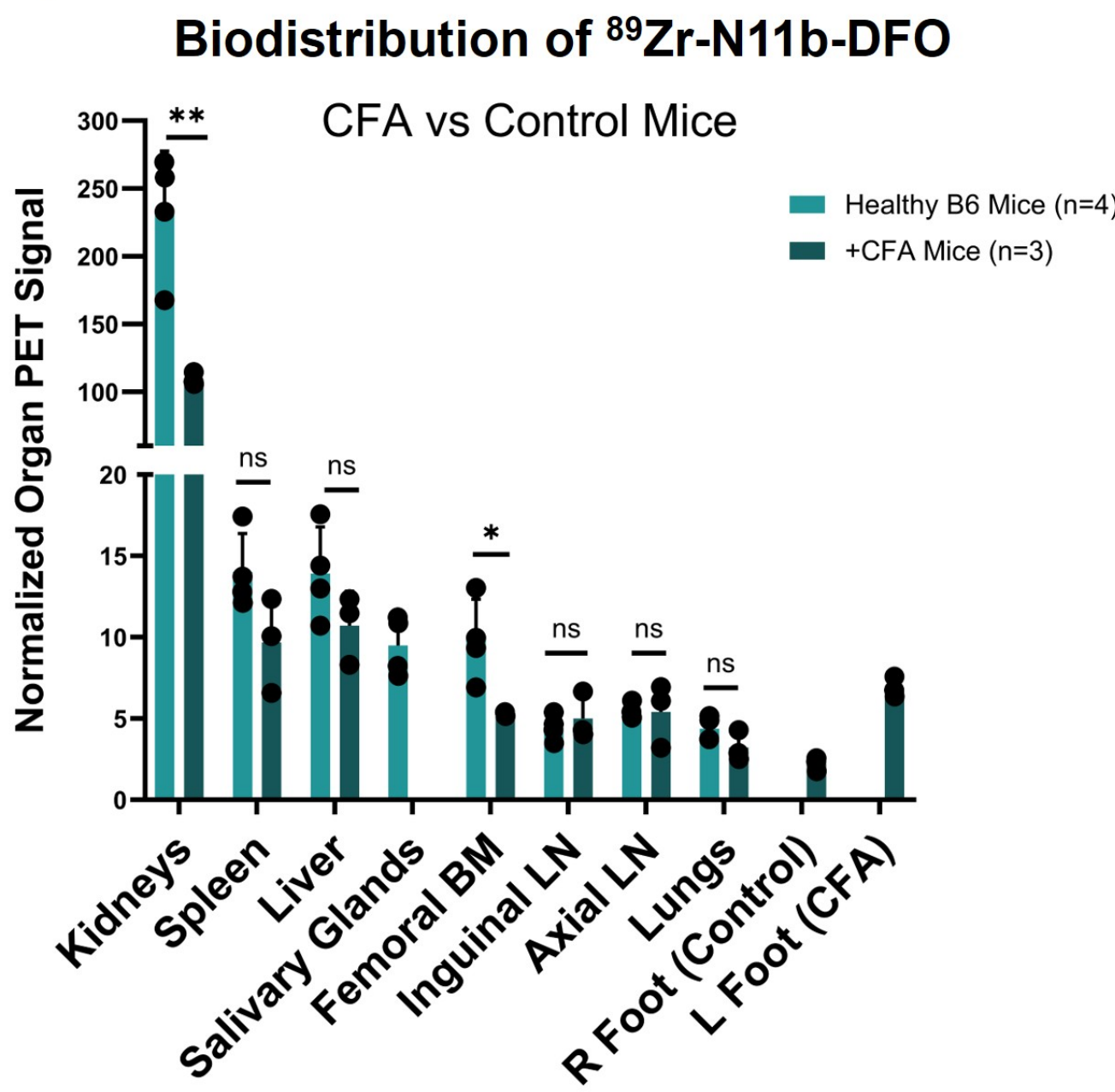

Figure S8: Organ-level biodistribution of **^{89}Zr-N11b-DFO**. a) Regular photograph of a C57BL/6 mouse administered with 50 µL of CFA intraplantarly into the left paw prior to PET imaging. b) Normalized organ PET signal values were used to compare the in vivo distribution of **^{89}Zr-N11b-DFO** between healthy mice (n=4) and those with CFA-induced paw inflammation (n=3). Only organs consistently included within the field of view for the respective scans were analyzed. Significance in the bar graph was tested using a Welch's t test and is indicated as *P< 0.05, **P< 0.01, and ***P<0.001.

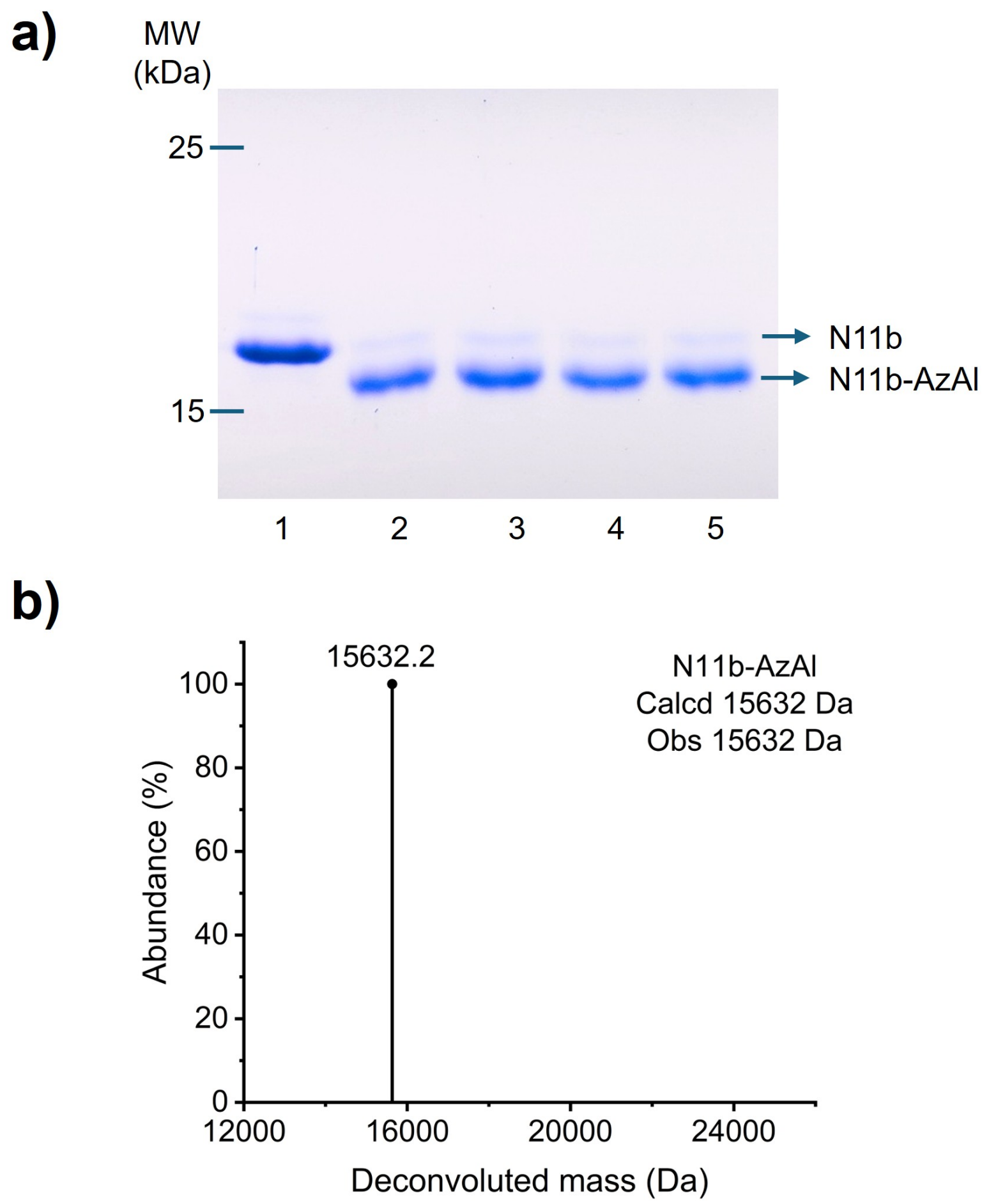

Figure S9: SDS PAGE and LC-ESI-MS analysis of **N11b-AzAl**. FTase-mediated modification of **N11b** using compound **2** yielded **N11b-AzAl**. a) SDS-PAGE analysis of the prenylation reaction using 5 μM **N11b** and 400 nM FTase with varying concentrations of compound **2**. Lane 1: unmodified **N11b**; Lane 2: reaction using 10 μM **2**; Lane 3: reaction using 20 μM **2**; Lane 4: reaction using 30 μM **2**; Lane 5: reaction using 40 μM **2**. Prenylation reached ≥96% conversion at 10 μM, with no further increase observed up to 40 μM. Uncropped gel provided in uncropped gels section. b) Deconvoluted mass spectrum of **N11b-AzAl**. Calcd: 15632 Da, Obs: 15632 Da.

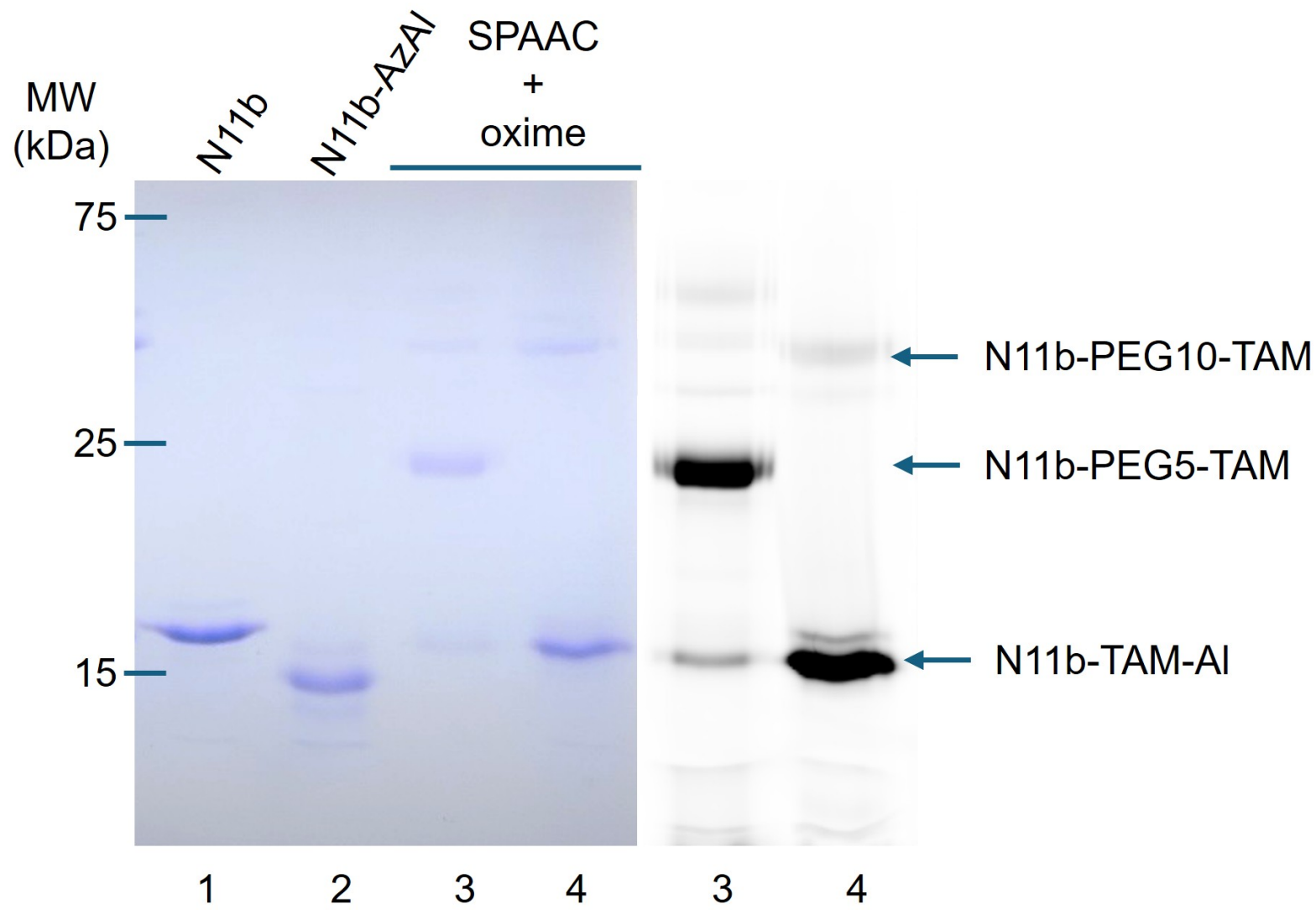

Figure S10. SDS-PAGE analysis of dual labeling at pH 6.0 with pPDA. Simultaneous SPAAC with TAMRA-DBCO and oxime ligation with the specified mPEG on **N11b-AzAl** was carried out at pH 6.0, with oxime formation catalyzed by 100 mM pPDA. The reaction mixture was loaded directly onto the gel to assess product conversion. Left: Coomassie-stained gel. Lane 1: **N11b**; Lane 2: **N11b-AzAl**; Lane 3: dual labeling with TAMRA-DBCO and aminooxy-mPEG (5 kDa), yielding **N11b-PEG5-TAM**; Lane 4: dual labeling with TAMRA-DBCO and aminooxy-mPEG (10 kDa), showing efficient SPAAC but poor oxime ligation, yielding predominantly non-PEGylated **N11b-TAM-Al**. The faint PEGylated product band, arising from successful completion of both reactions, is indicated by the top blue arrow. Right: Corresponding fluorescence (TAMRA) scan of the same gel. Uncropped gel provided in uncropped gels section.

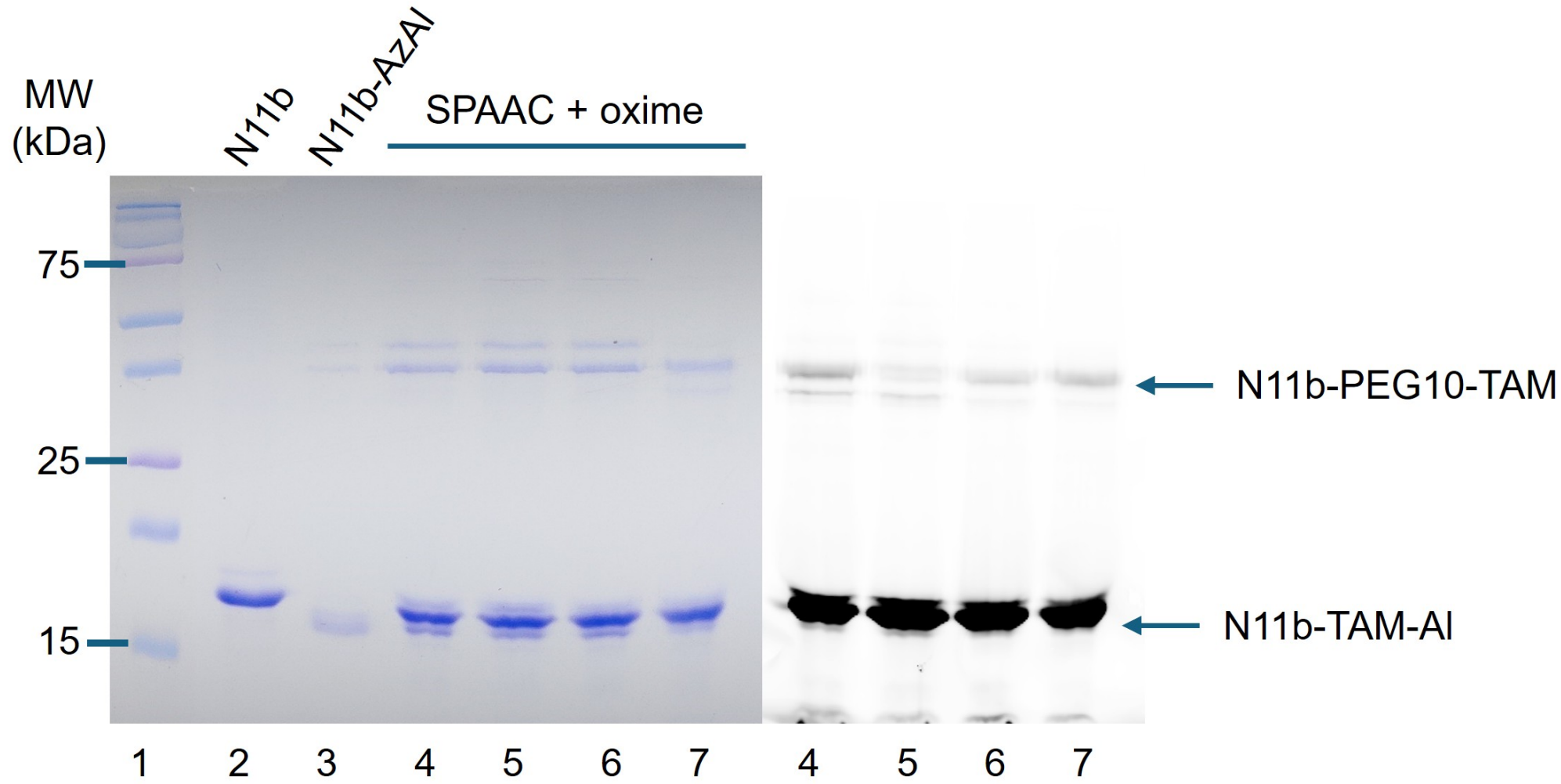

Figure S11. SDS-PAGE analysis of dual labeling using different amine catalysts at pH=6.0. Simultaneous SPAAC with TAMRA-DBCO and oxime ligation with aminooxy-mPEG (10 kDa) was performed on **N11b-AzAl** using the specified amine catalysts, and the reaction mixtures were loaded directly onto the gel to assess product conversion. Left: Coomassie-stained gel. Lane 1: protein ladder; Lane 2: **N11b**; Lane 3: **N11b-AzAl**; Lane 4: reaction containing 10 mM anthranilic acid; Lane 5: reaction containing 10 mM 3,5-diaminobenzoic acid; Lane 6: reaction containing 40 mM mPDA; Lane 7: reaction containing 200 mM pPDA. Right: Corresponding fluorescence (TAMRA) scan of the same gel. There was an empty lane between lane 1 and lane 2 which was removed and the lane 1 and 2 are spliced together. Uncropped gel provided in uncropped gels section.

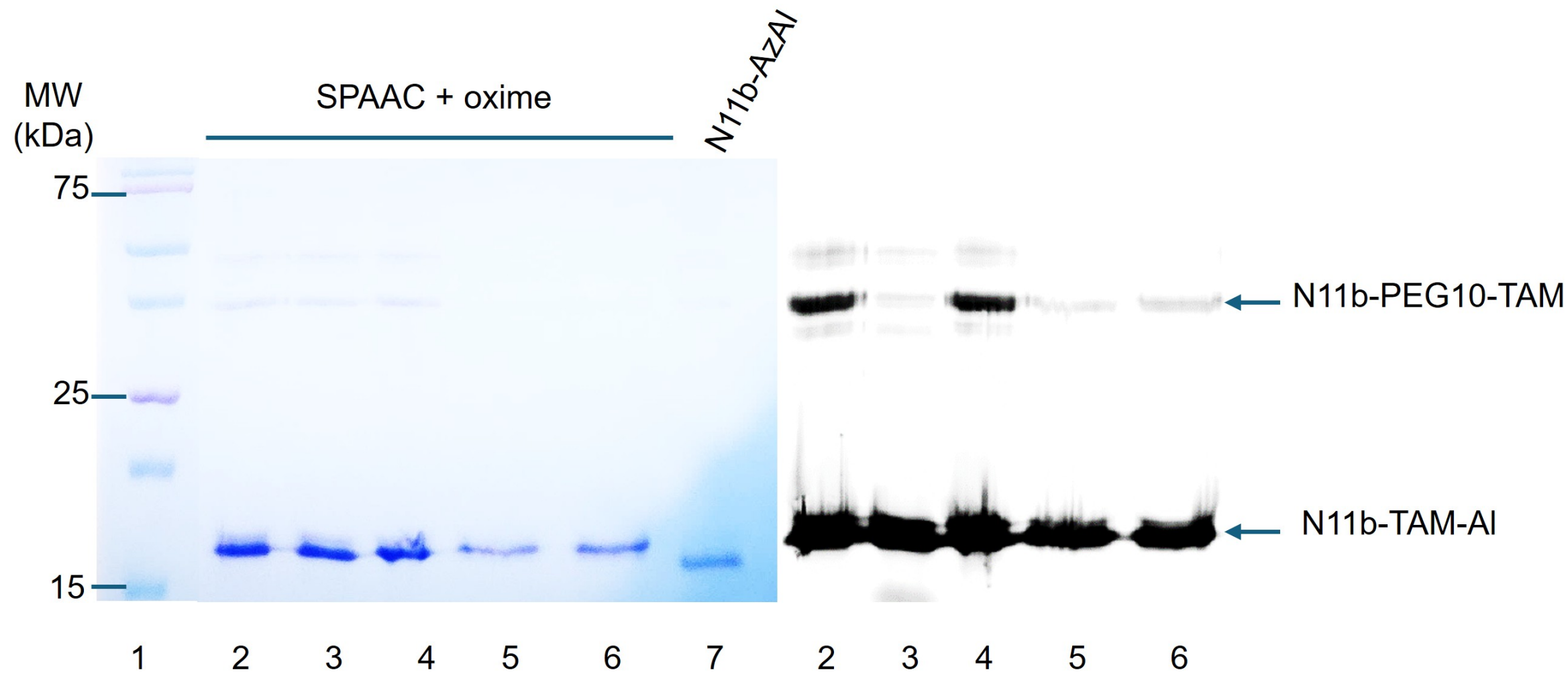

Figure S12. SDS-PAGE analysis of dual labeling at pH 5.5 and 6.0 with various amine catalysts. Simultaneous SPAAC and oxime ligation with aminooxy-mPEG (10 kDa) was carried out on **N11b-AzAl** using the specified amine catalyst and pH, and reaction mixtures were loaded directly onto the gel to assess product conversion. Left: Coomassie-stained gel. Lane 1: protein ladder; Lane 2: reaction containing 100 mM 5-methoxy anthranilic acid (pH 5.5); Lane 3: reaction containing 100 mM 3,5-diaminobenzoic acid (pH 5.5); Lane 4: reaction containing 50 mM 5-methoxy anthranilic acid (pH 5.5); Lane 5: reaction containing 400 mM pPDA (pH 6.0); Lane 6: reaction containing 500 mM pPDA (pH 6.0); Lane 7: **N11b-AzAl**. Right: Corresponding fluorescence (TAMRA) scan of the same gel. There was an empty lane between lane 1 and lane 2 which was removed and the lane 1 and 2 are spliced together. Uncropped gel provided in uncropped gels section.

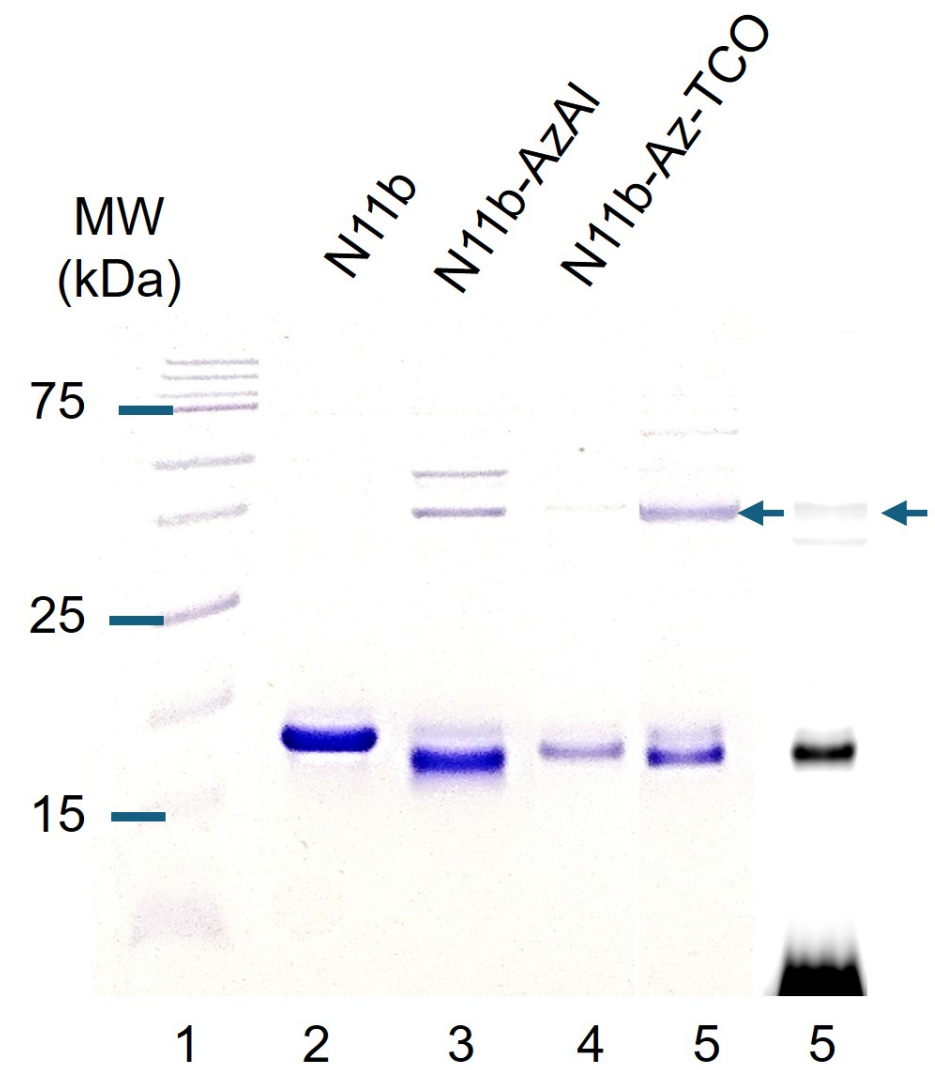

Figure S13: SDS-PAGE analysis of dual labeling on **N11b-Az-TCO**. Left: Coomassie-stained gel. Lane 1: protein ladder; Lane 2: **N11b**; Lane 3: **N11b-AzAl**; Lane 4: **N11b-Az-TCO**; Lane 5: **N11b-TCO** subjected to one-pot SPAAC and tetrazine ligation using TAMRA-DBCO and methyltetrazine-mPEG (10 kDa). The reaction mixture was loaded directly onto the gel to assess product conversion. Right: Corresponding fluorescence (TAMRA) scan of the same gel. Uncropped gel provided in uncropped gels section.

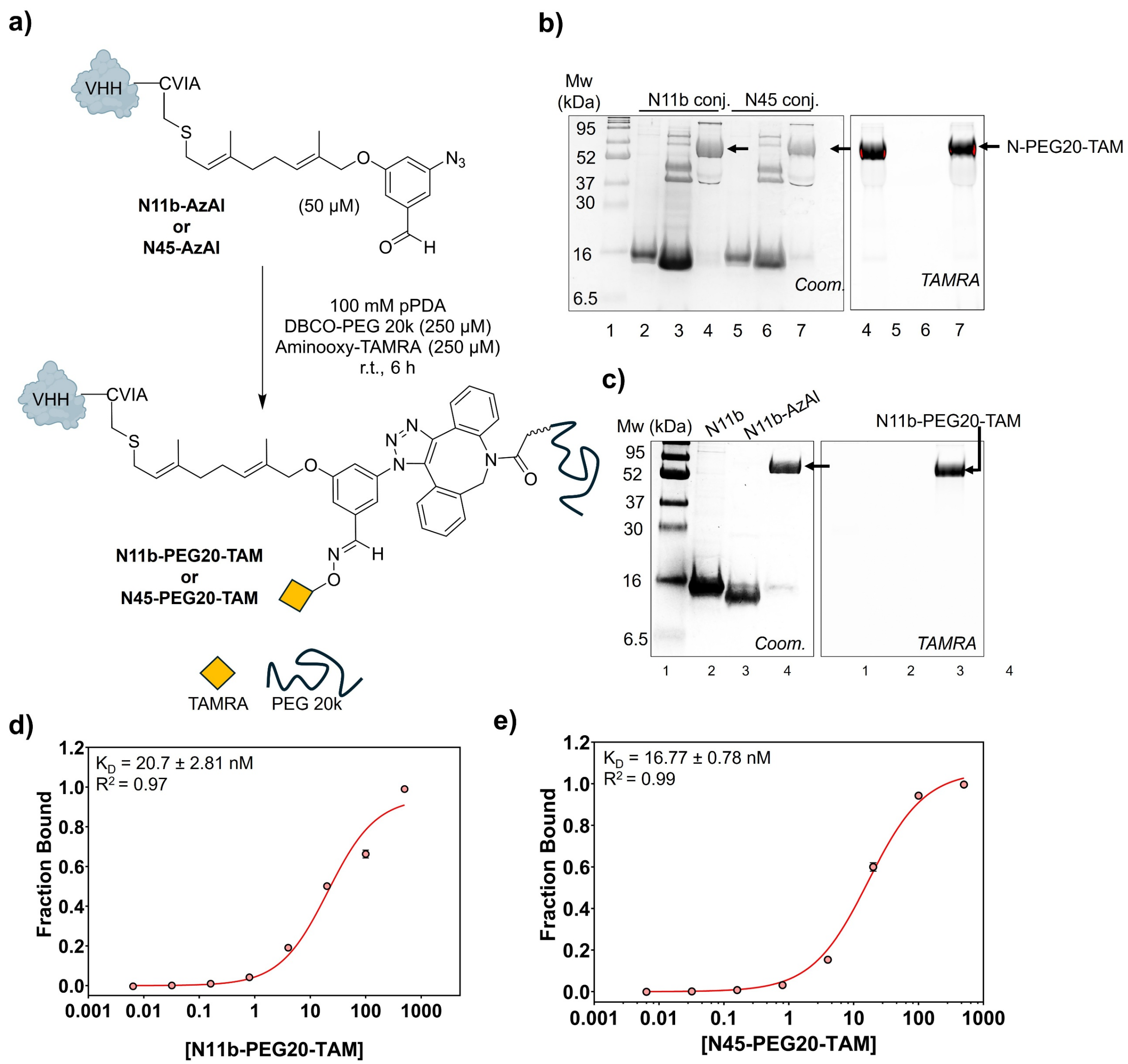

Figure S14: FTase-catalyzed dual labeling of **N11b** and **N45** using trifunctional FPP analog **2** for one-pot installation of higher-molecular-weight PEG (≥10 kDa) and TAMRA. a) Schematic representation of the one-pot conjugation strategy used to install TAMRA and 20 kDa PEG. b) SDS-PAGE analysis of the one-pot labeling reactions showed a higher-molecular-weight fluorescent band at ~60 kDa in lanes 4 and 7, corresponding to **N11b-PEG20-TAM** and **N45-PEG20-TAM**, respectively, indicating near-complete conversion and successful incorporation of both 20 kDa PEG and TAMRA. Lane 1: protein ladder; lane 2: **N11b**; lane 3: crude **N11b-AzAl**; lane 4: desalted one-pot dual-labeling reaction of **N11b-AzAl** with aminooxy-TAMRA and DBCO-PEG20 kDa, yielding **N11b-PEG20-TAM**; lane 5: **N45**; lane 6: crude **N45-AzAl**; lane 7: desalted one-pot dual-labeling reaction of **N45-AzAl** with aminooxy-TAMRA and DBCO-PEG 20 kDa, yielding **N45-PEG20-TAM**. c) SDS-PAGE analysis of purified **N11b-PEG20-TAM**. Lane 1: protein ladder; lane 2: **N11b**; lane 3: purified **N11b-AzAl**; lane 4: purified **N11b-PEG20-TAM**. d,e) Flow cytometry-based affinity analysis of the PEGylated nanobodies gave K_D values of 20.7 ± 2.81 nM for **N11b-PEG20-TAM** and 16.77 ± 0.78 nM for **N45-PEG20-TAM**.

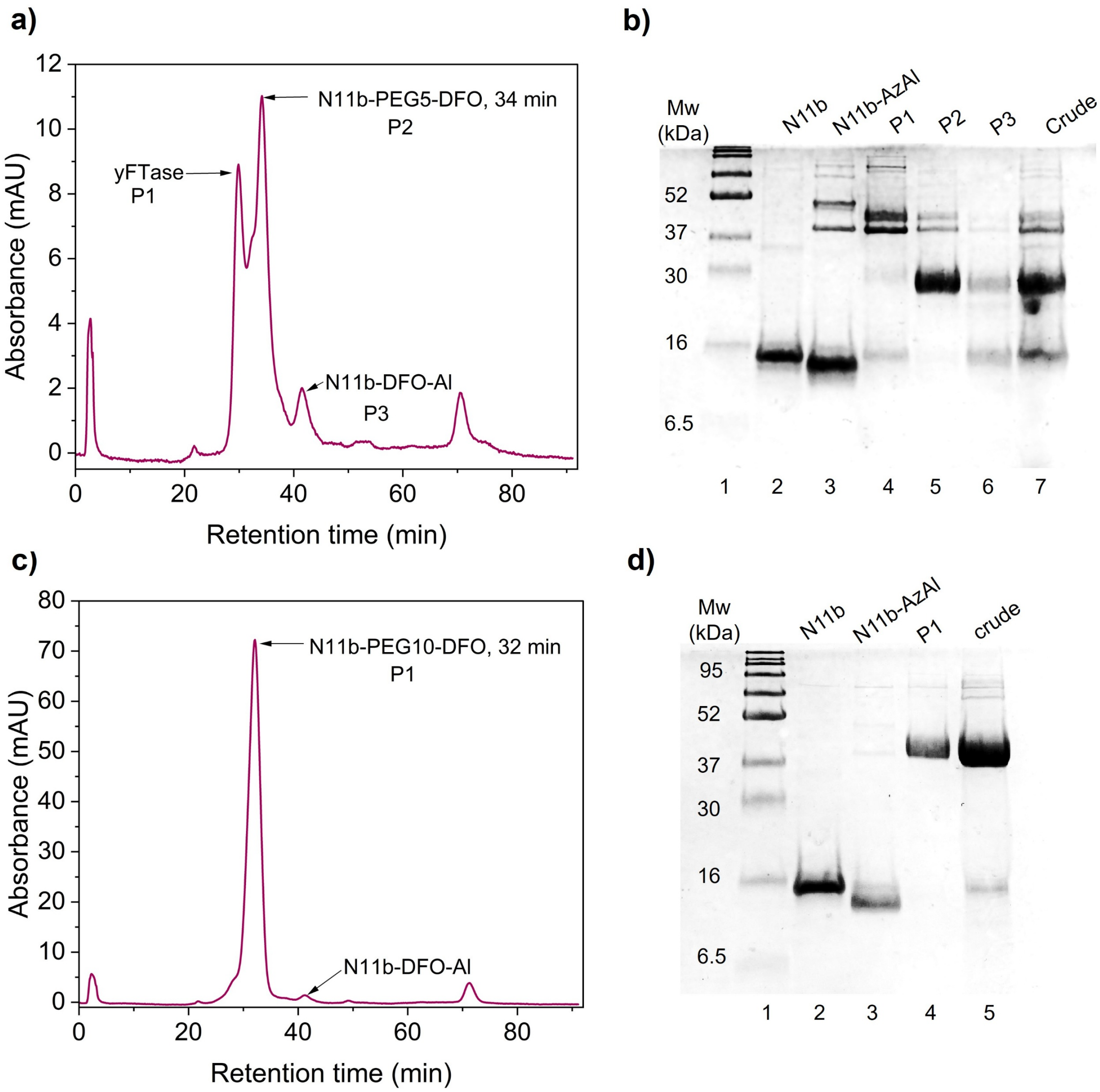

Figure S15: SDS-PAGE analysis and SEC purification of **N11b-PEG5-DFO** and **N11b-PEG10-DFO**. a) SEC chromatogram (280 nm) of **N11b-PEG5-DFO**. Peak P1 (29.9-31 min) corresponds to FTase, P2 (~34 min) to **N11b-PEG5-DFO**, and P3 (~42 min) to **N11b-DFO-Al**. Partial overlap was observed between the FTase and **N11b-PEG5-DFO** peaks. (b) SDS-PAGE analysis of SEC fractions from panel a. Lane 1: protein ladder; lane 2: unmodified N11b; lane 3: crude **N11b-AzAl**; lane 4: P1 fraction (27-31 min), containing predominantly FTase (two bands between 37-52 kDa); lane 5: P2 fraction, containing ~89% **N11b-PEG5-DFO** (~30 kDa) with trace FTase carryover; lane 6: P3 fraction, containing trace amount of **N11b-DFO-Al** (~16 kDa) and trace **N11b-PEG5-DFO** carryover; lane 7: crude **N11b-PEG5-DFO** sample prior to SEC purification, containing 15% FTase, 77% **N11b-PEG5-DFO**, and 8% N11b-DFO-Al. Compositions were determined by densitometric analysis of the SDS-PAGE gel. (c) SEC chromatogram (280 nm) of **N11b-PEG10-DFO**. Peak P1 (~32 min) corresponds to **N11b-PEG10-DFO.** (d) SDS-PAGE analysis of SEC fractions from panel c. Lane 1: protein ladder; lane 2: unmodified **N11b**; lane 3: crude **N11b-AzAl**; lane 4: P1 fraction containing **N11b-PEG10-DFO** (b/w 37-52 kDa); lane 5: crude **N11b-PEG10-DFO** sample prior to SEC purification, containing 96% **N11b-PEG10-DFO** and 4% **N11b-DFO-Al**.

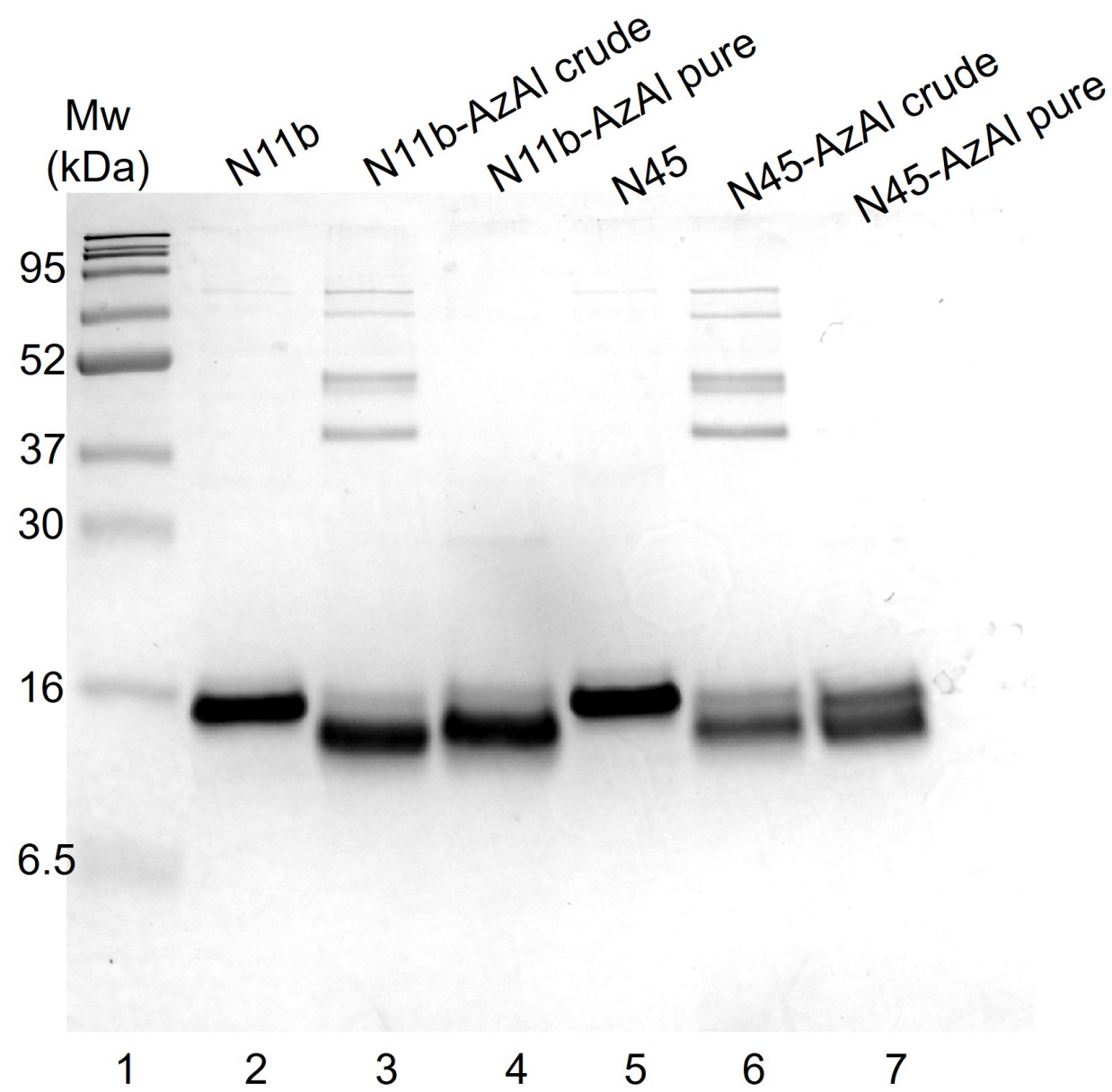

Figure S16: SDS-PAGE analysis of anti-FLAG affinity purification of **N11b-AzAl** and **N45-AzAl** conjugates. Anti-FLAG resin selectively captured FLAG-tagged nanobody species, allowing FTase to be removed in the wash fractions. Bound nanobodies were subsequently eluted using buffer containing 1.75 M NaSCN. Lane 1: protein ladder; lane 2: **N11b**; lane 3: crude **N11b-AzAl**; lane 4: anti-FLAG-purified **N11b-AzAl**; lane 5: **N45**; lane 6: crude **N45-AzAl**; lane 7: anti-FLAG-purified **N45-AzAl**.

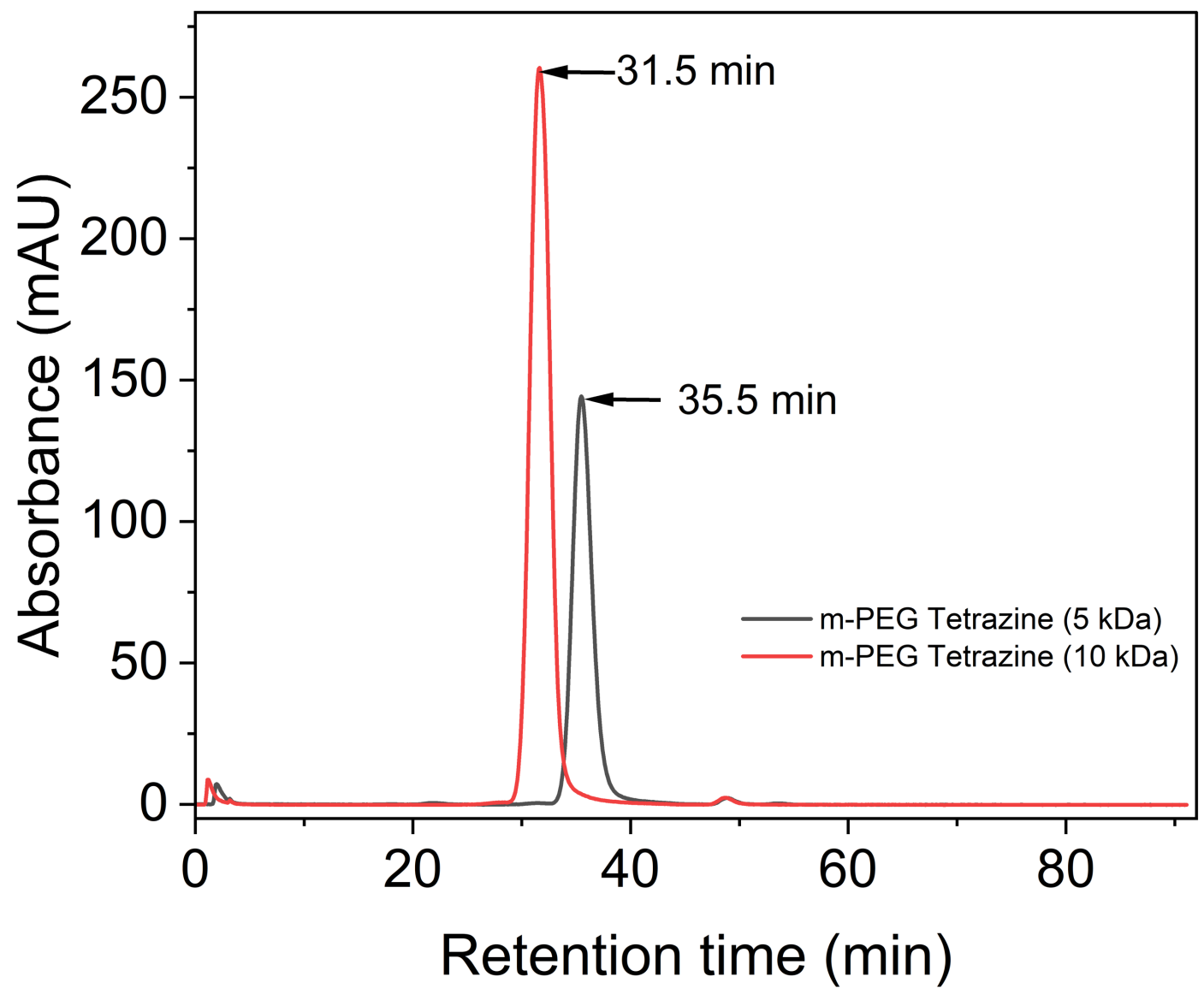

Figure S17: SEC chromatograms (280 nm) of mPEG-tetrazine reagents (5 and 10 kDa). The 5 kDa and 10 kDa mPEG-tetrazine reagents eluted at 35.5 and 31.5 min, respectively. Samples were prepared at 200 μM in PBS (500 μL injection volume).

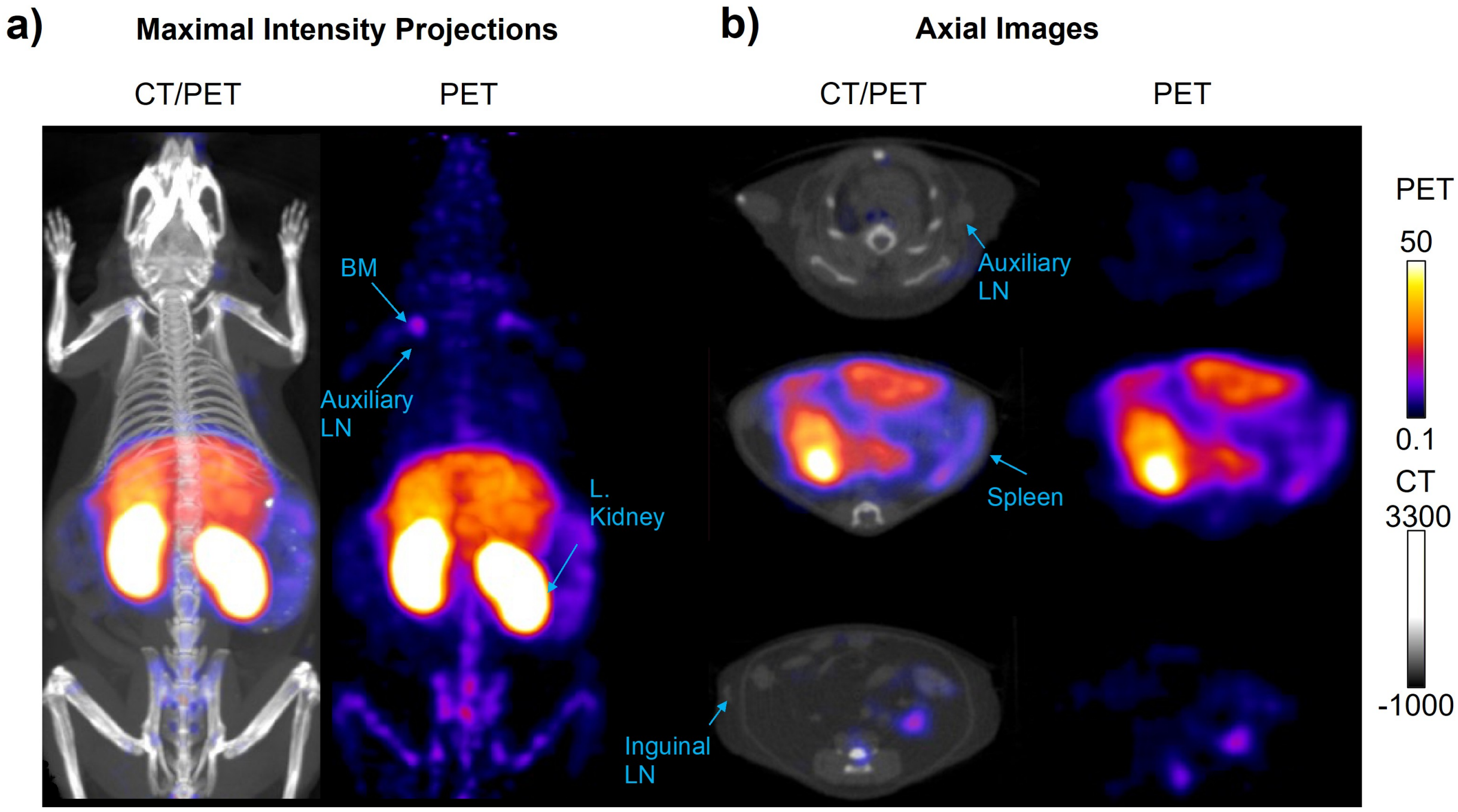

Figure S18: PET imaging of healthy mice following administration of the **^{89}Zr-N11b-PEG10-DFO.** Healthy C57BL/6 mice (n=5) was injected with around 50 μCi of the imaging agent, followed by PET/CT imaging after 12 h. The probe visualizes immune-cell-rich organs of healthy mice. a) Representative maximum-intensity projection (MIP) images of CT/PET (left) and PET alone (right). b) Representative axial images of CT/PET (left) and PET alone (right). The PET and CT scale bars are in μCi mm^{-3} and Hounsfield units, respectively.

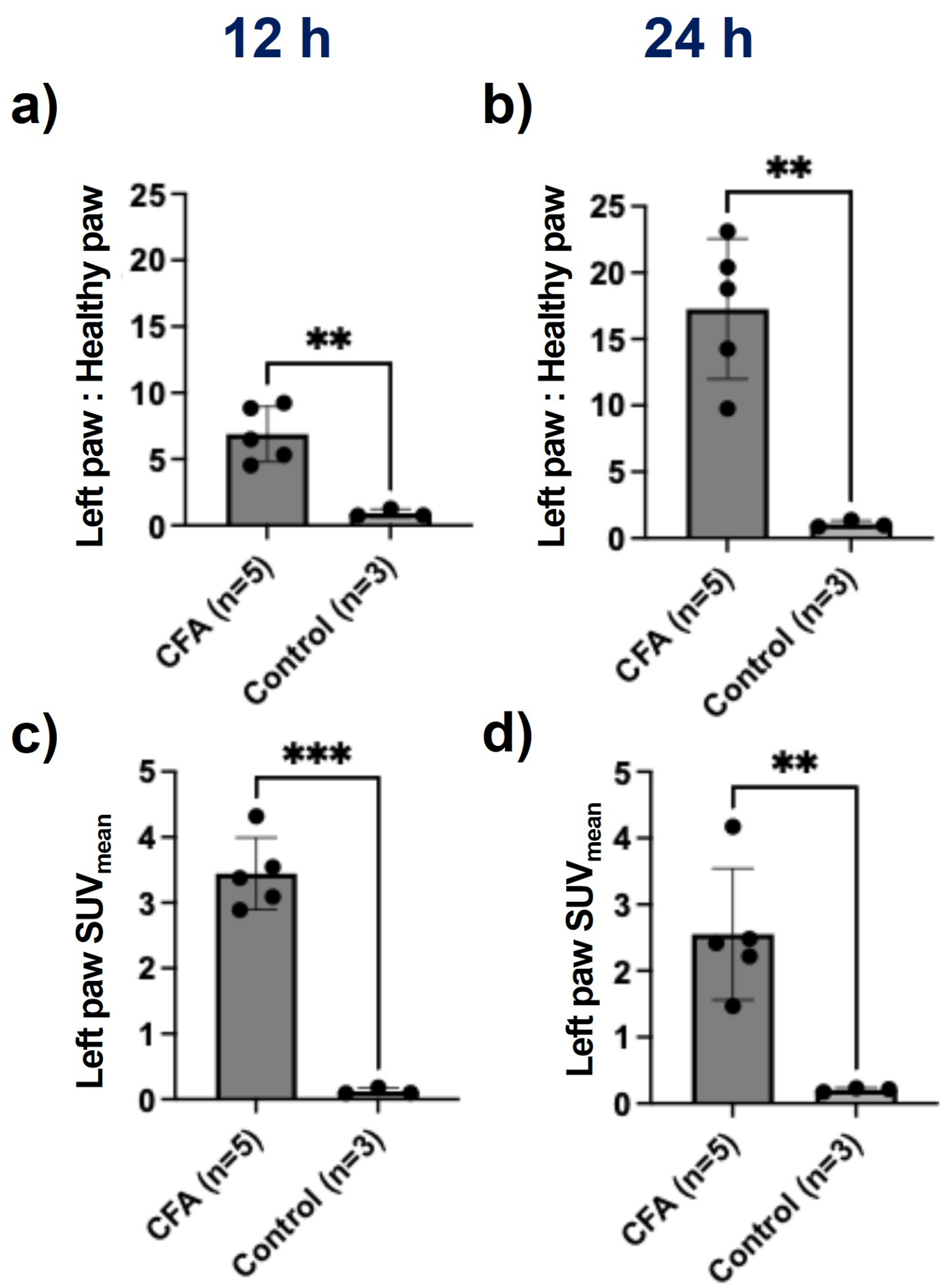

Figure S19: Quantitative comparison of the paw SUV_{mean} in PET imaging using **^{89}Zr-N11b-PEG10-DFO**. Comparison of left-to-right paw SUV_{mean} ratios between control and CFA-induced mice at a) 12 h and b) 24 h. Comparison of SUV_{mean} at the left paw between control and CFA-induced inflammation mice at c) 12 h and d) 24 h. Significance in the bar graph was tested using a Welch's t test and is indicated as *P< 0.05, **P< 0.01, and ***P<0.001.

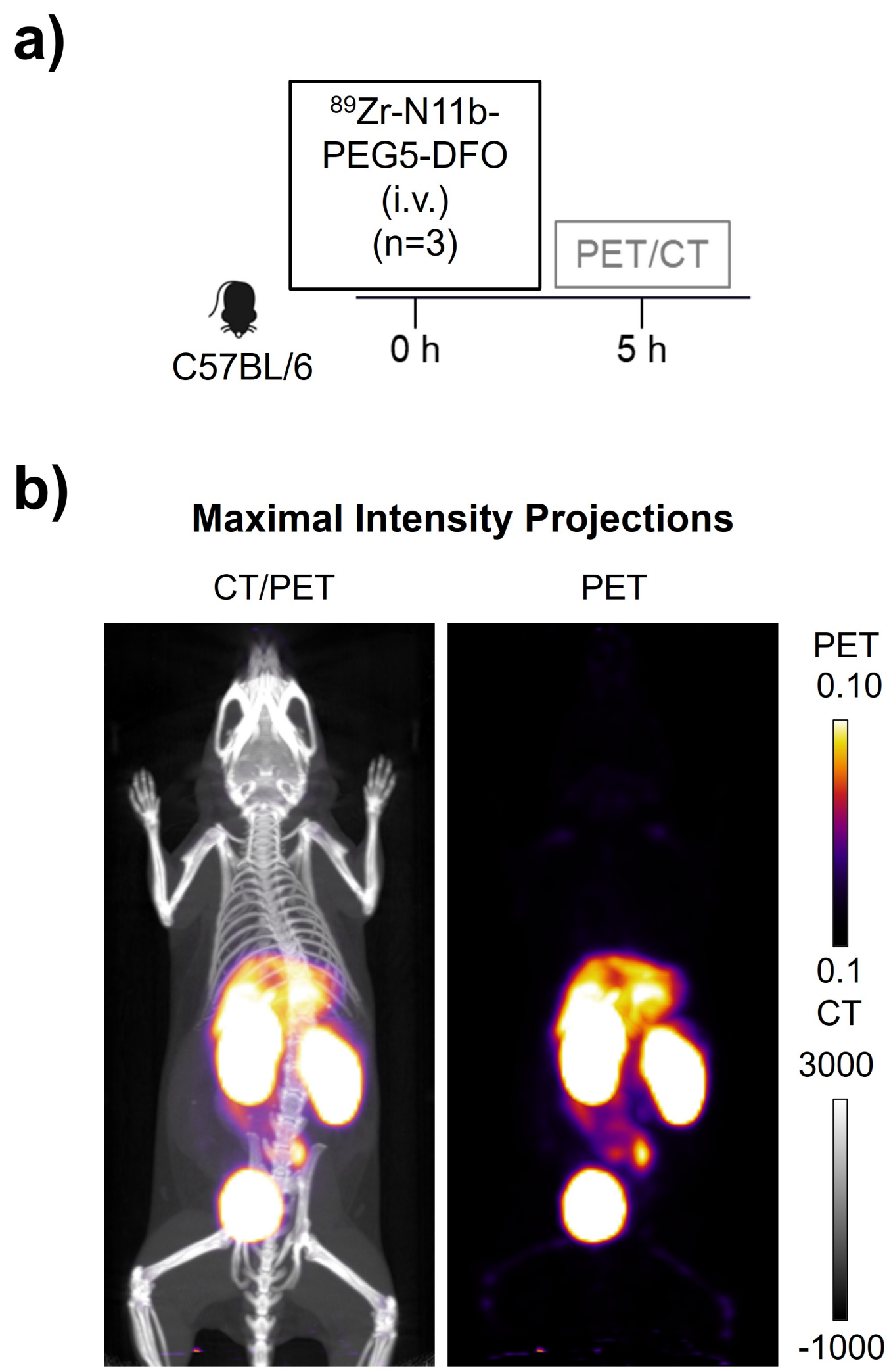

Figure S20: PET imaging of healthy mice with ^{89}**Zr-N11b-PEG5-DFO**. a) PET imaging of healthy C57BL/6 mice (n = 3) was performed after intravenous administration of 50 μCi of ^{89}**Zr-N11b-PEG5-DFO**, with imaging conducted 5 h post-injection. b) Representative maximum-intensity projection (MIP) images of CT/PET (left) and PET alone (right). The PET and CT scale bars are in μCi mm^{-3} and Hounsfield units, respectively.

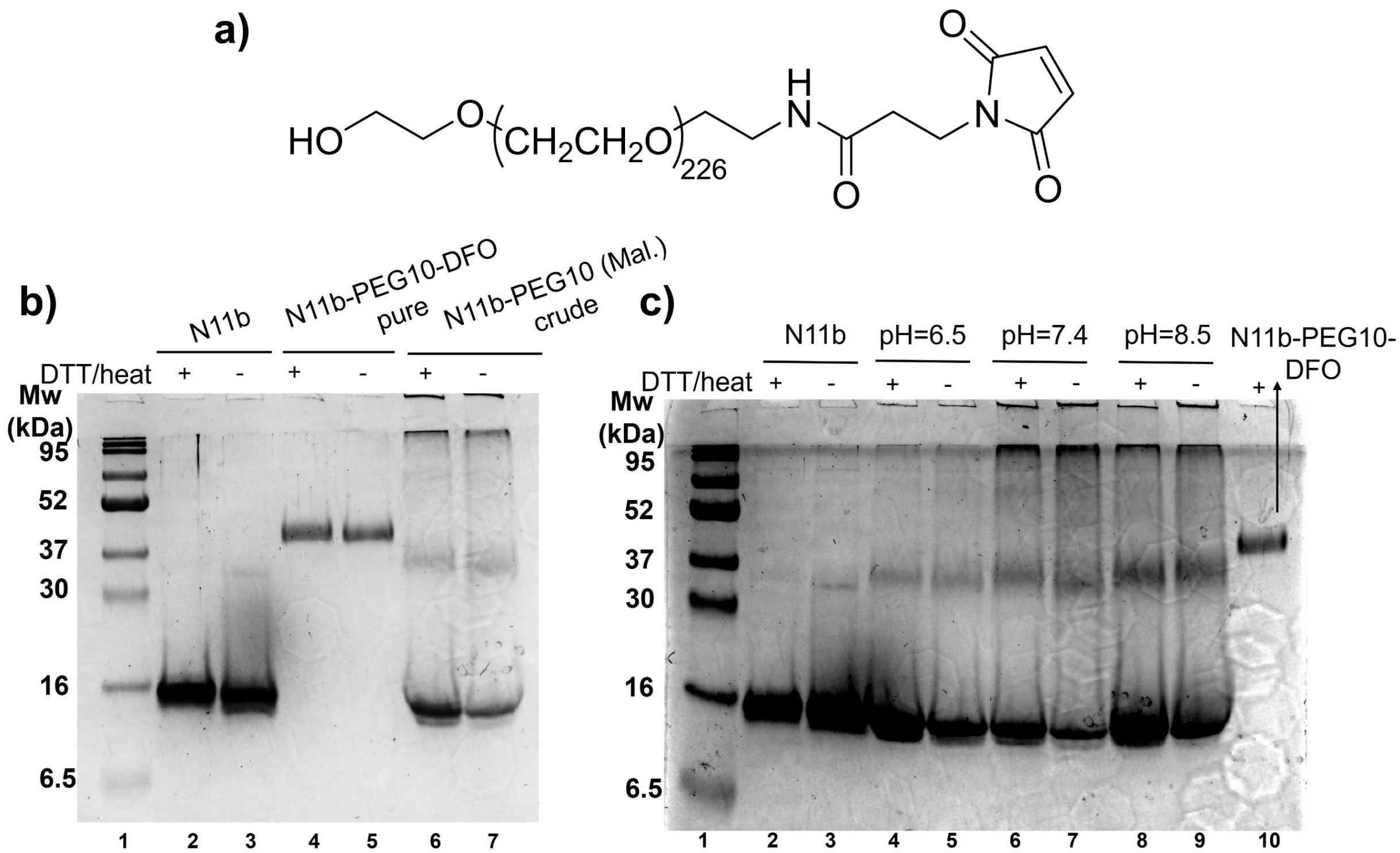

Figure S21: Maleimide-thiol based PEGylation of the C-terminal CaaX cysteine of **N11b**. a) Structure of the maleimide-PEG reagent used for conjugation (MAL-PEG-OH, 10 kDa; Creative PEGWorks, Cat. No. PHB-946). b) Head-to-head comparison of the FTase-based labeling strategy and direct maleimide-thiol conjugation at pH 7.4. SDS-PAGE analysis of the crude maleimide reaction performed overnight in PBS (pH 7.4) using a 1:10 molar ratio of **N11b** to maleimide-PEG10 kDa. Lane 1: protein ladder; lane 2: unmodified **N11b** (+DTT/heat); lane 3: unmodified **N11b** (−DTT/heat); lane 4: purified **N11b-PEG10-DFO** prepared by the FTase strategy (+DTT/heat); lane 5: purified **N11b-PEG10-DFO** prepared by the FTase strategy (−DTT/heat); lane 6: crude N11b-PEG10 from the maleimide reaction (+DTT/heat); lane 7: crude N11b-PEG10 from the maleimide reaction (−DTT/heat). The slower mobility of **N11b-PEG10-DFO** relative to maleimide-derived N11b-PEG10 is consistent with the additional DFO group. The +DTT/heat condition confirms that the band near ~37 kDa is not due to nanobody dimerization, while the −DTT/heat sample of unmodified N11b indicates that the nanobody remained reduced following TCEP treatment prior to maleimide addition. c) SDS-PAGE analysis of maleimide-thiol conjugation performed at different pH values (6.5-8.5) to compare reaction efficiency. Lane 1: protein ladder; lane 2: **N11b** (+DTT/heat); lane 3: **N11b** (−DTT/heat); lanes 4-5: reaction at pH 6.5 (+DTT/heat and −DTT/heat, respectively); lanes 6-7: reaction at pH 7.4 (+DTT/heat and −DTT/heat, respectively); lanes 8-9: reaction at pH 8.5 (+DTT/heat and −DTT/heat, respectively); lane 10: **N11b-PEG10-DFO** prepared via the FTase-based strategy.

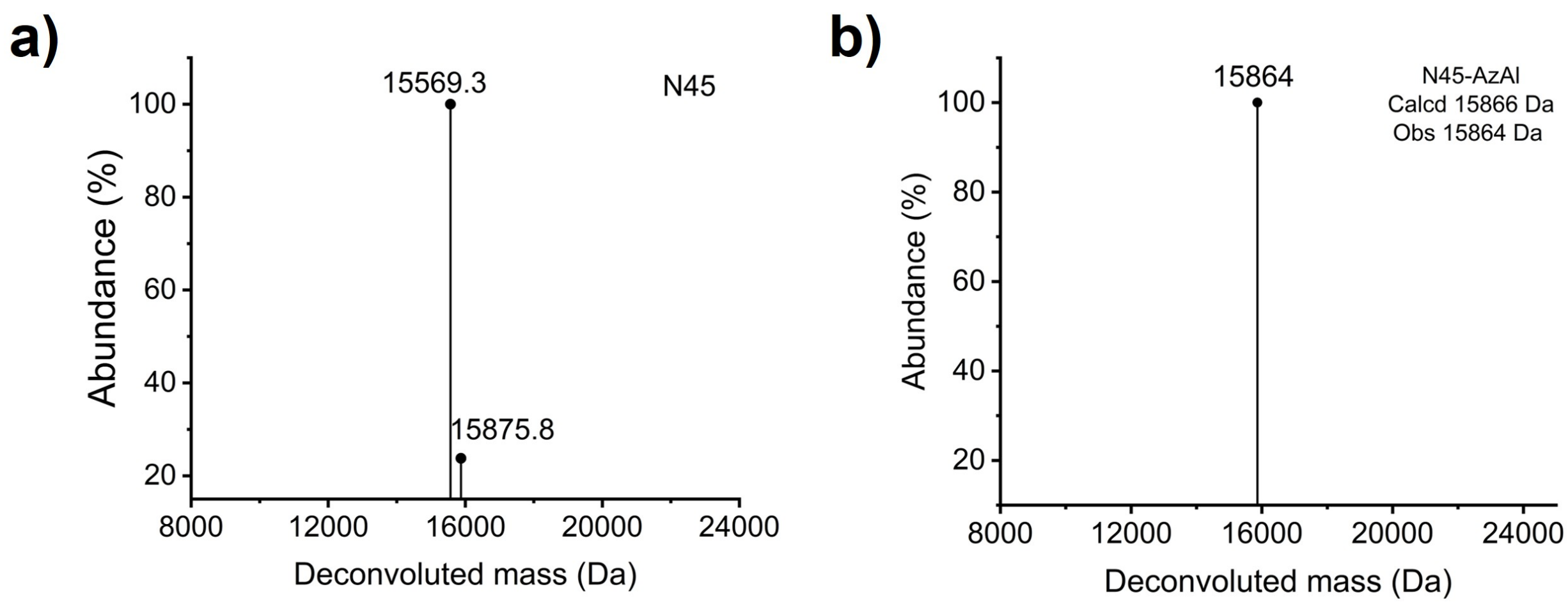

Figure S22: LC-ESI-MS analysis of **N45** and **N45-AzAl**. a) Deconvoluted mass spectrum of the **N45**. The Obs mass at 15,569 Da corresponds to N45, while the 15,875 Da peak represents a glutathione-conjugated form, indicating cysteine oxidation within the CVIA tag during the refolding process. b) Deconvoluted mass spectrum of **N45-AzAl**. Calcd: 15866 Da, Obs: 15864 Da.

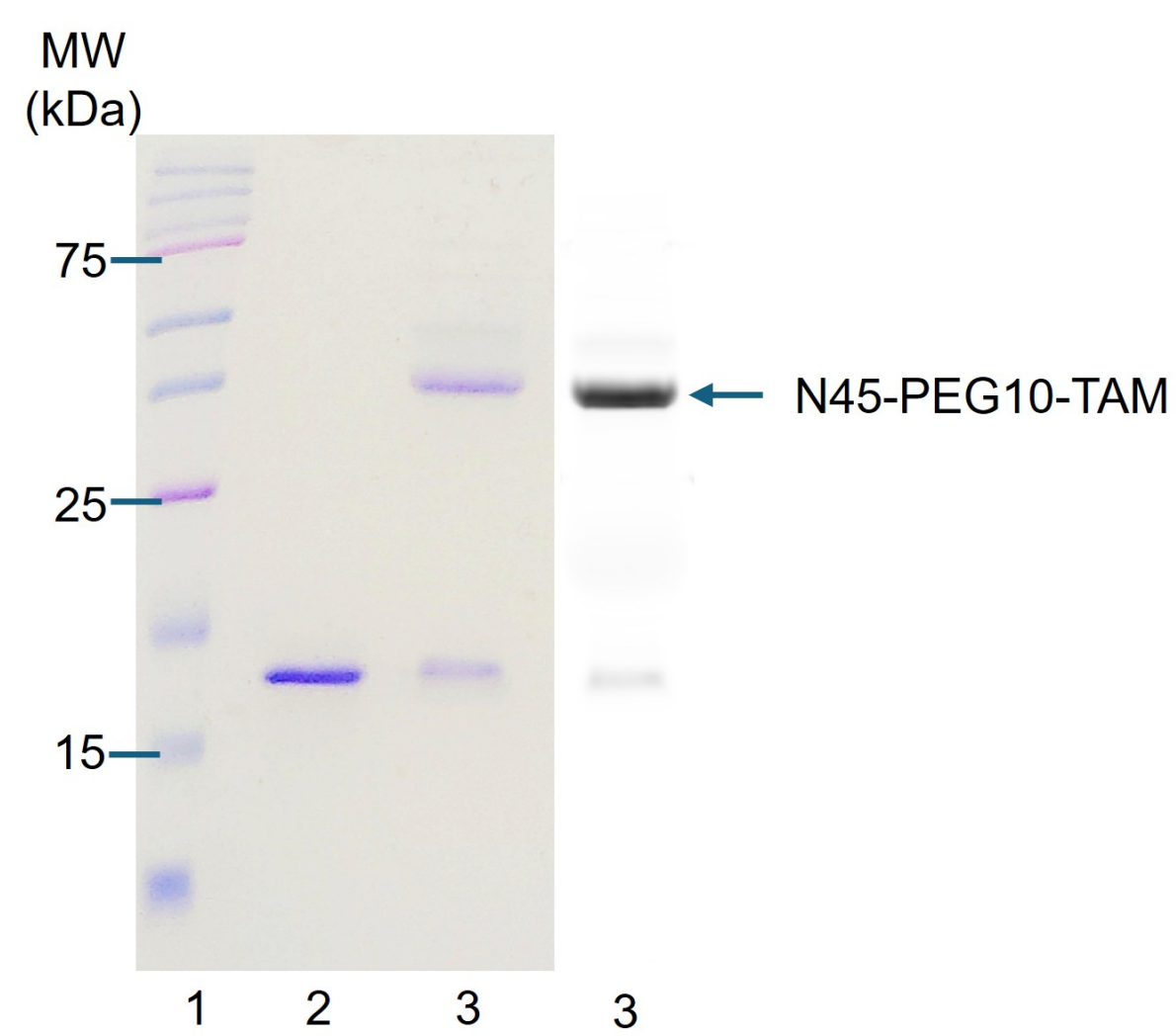

Figure S23: SDS-PAGE analysis of **N45-PEG10-TAM**. Left: Coomassie-stained gel. Lane 1: protein ladder; Lane 2: unmodified **N45**; Lane 3: **N45-PEG10-TAM**. Right: Corresponding fluorescence (TAMRA) scan of the same gel. The fluorescent band at a higher molecular weight indicated by blue arrow in lane 3 confirms successful conjugation of both PEG (10 kDa) and TAMRA. Uncropped gel provided in uncropped gels section.

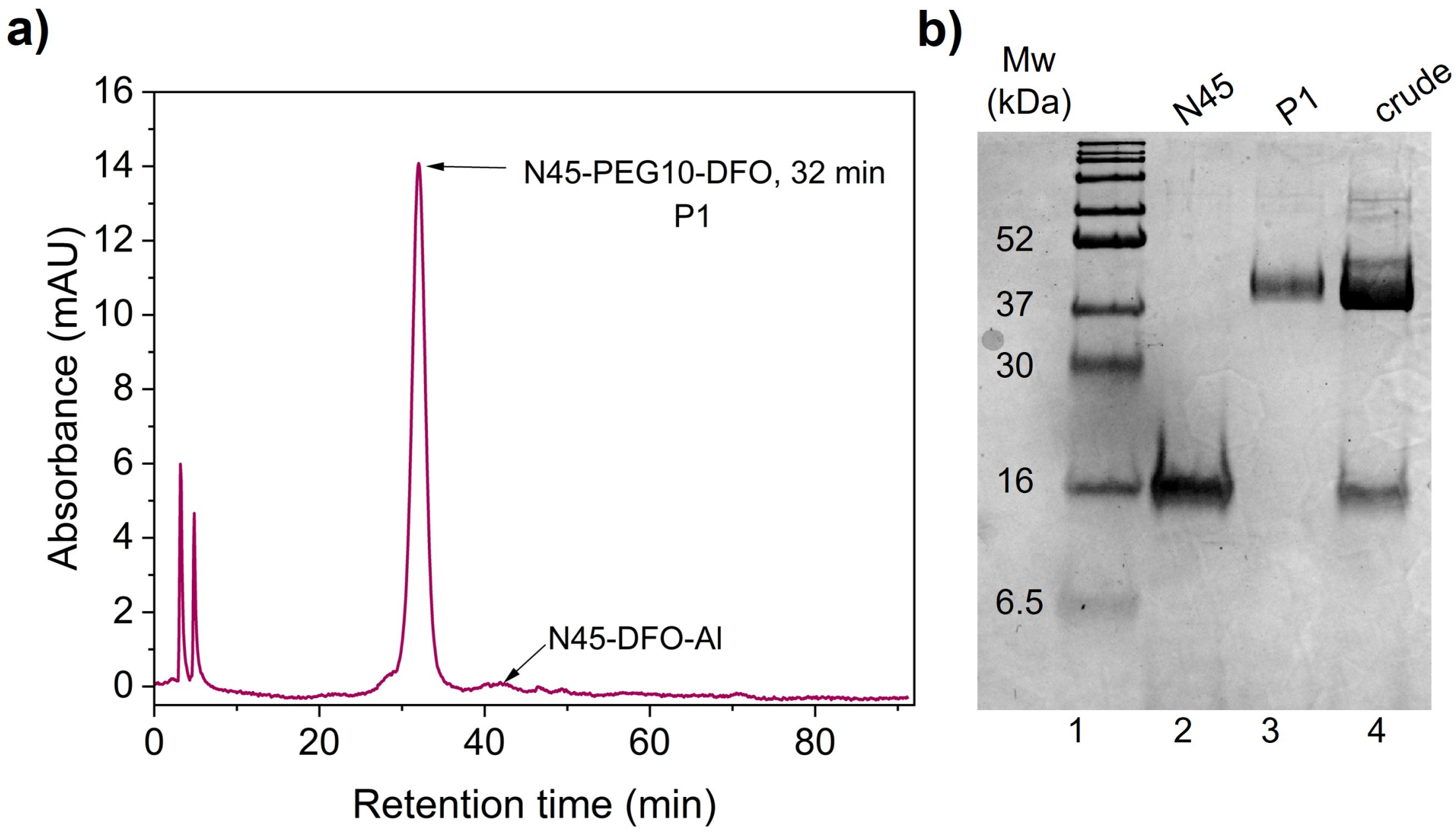

Figure S24: SEC purification of **N45-PEG10-DFO** and SDS PAGE analysis of the corresponding fractions. a) SEC chromatogram (280 nm) of **N45-PEG10-DFO**. Peak P1 (~32 min) corresponds to **N45-PEG10-DFO**. (b) SDS-PAGE analysis of SEC fractions from panel c. Lane 1: protein ladder; lane 2: unmodified **N45**; lane 3: P1 fraction containing **N45-PEG10-DFO**; lane 4: crude **N45-PEG10-DFO** sample prior to SEC purification, containing 90% **N45-PEG10-DFO** (between 37-52 kDa) and 10% **N45-DFO-Al** (~16 kDa).

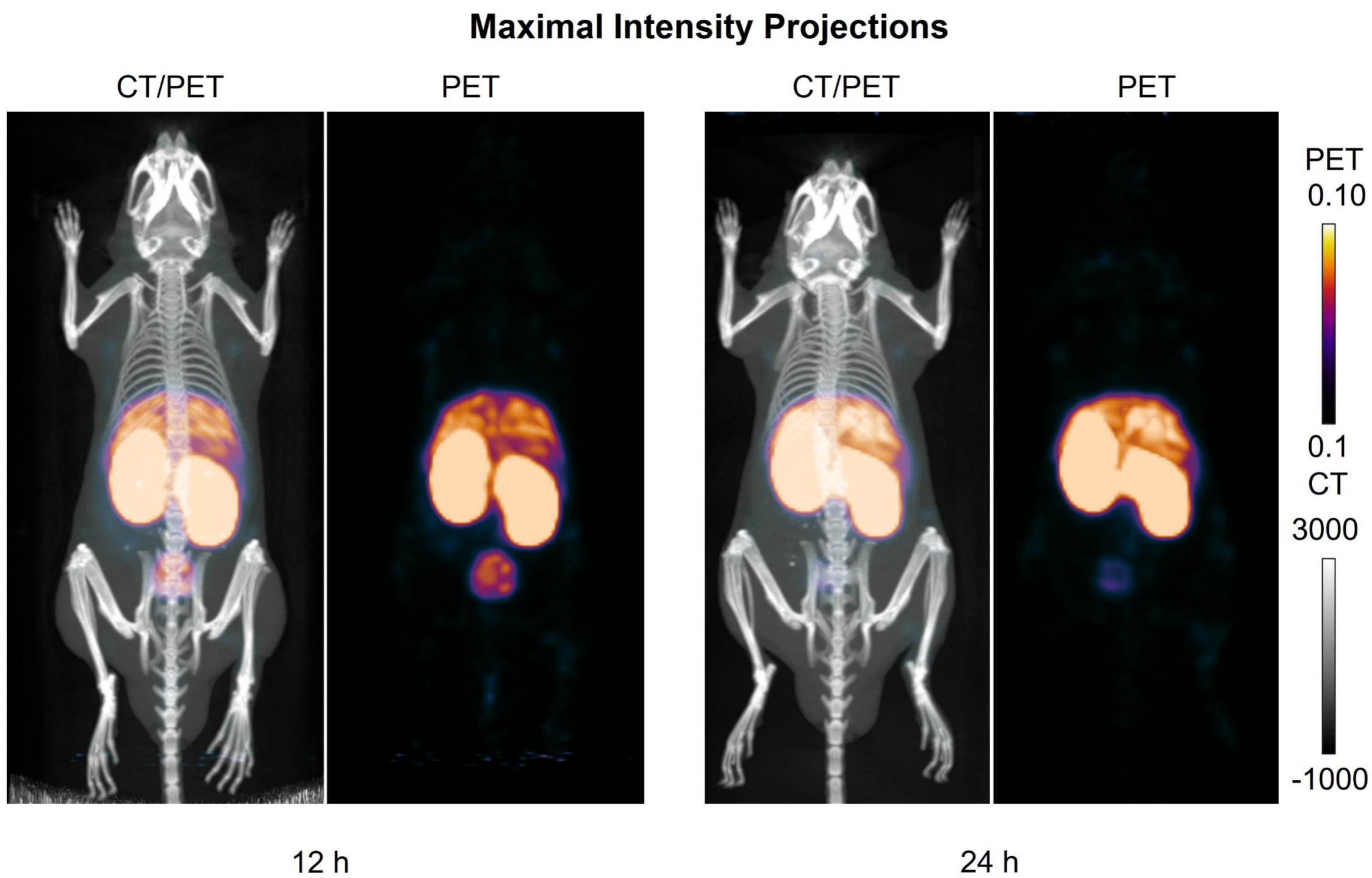

Figure S25: PET imaging of CFA-treated C57BL/6 mice was performed following intravenous administration of 50 μCi of **^{89}Zr-N45-PEG10-DFO** after CD45 pre-blocking with unmodified **N45**, with imaging conducted at 12 h and 24 h post-injection. Shown are representative maximum-intensity projection (MIP) images of CT/PET and PET alone at 12 h (left) and 24 h (right). PET and CT scale bars are in μCi mm^{-3} and Hounsfield units, respectively.

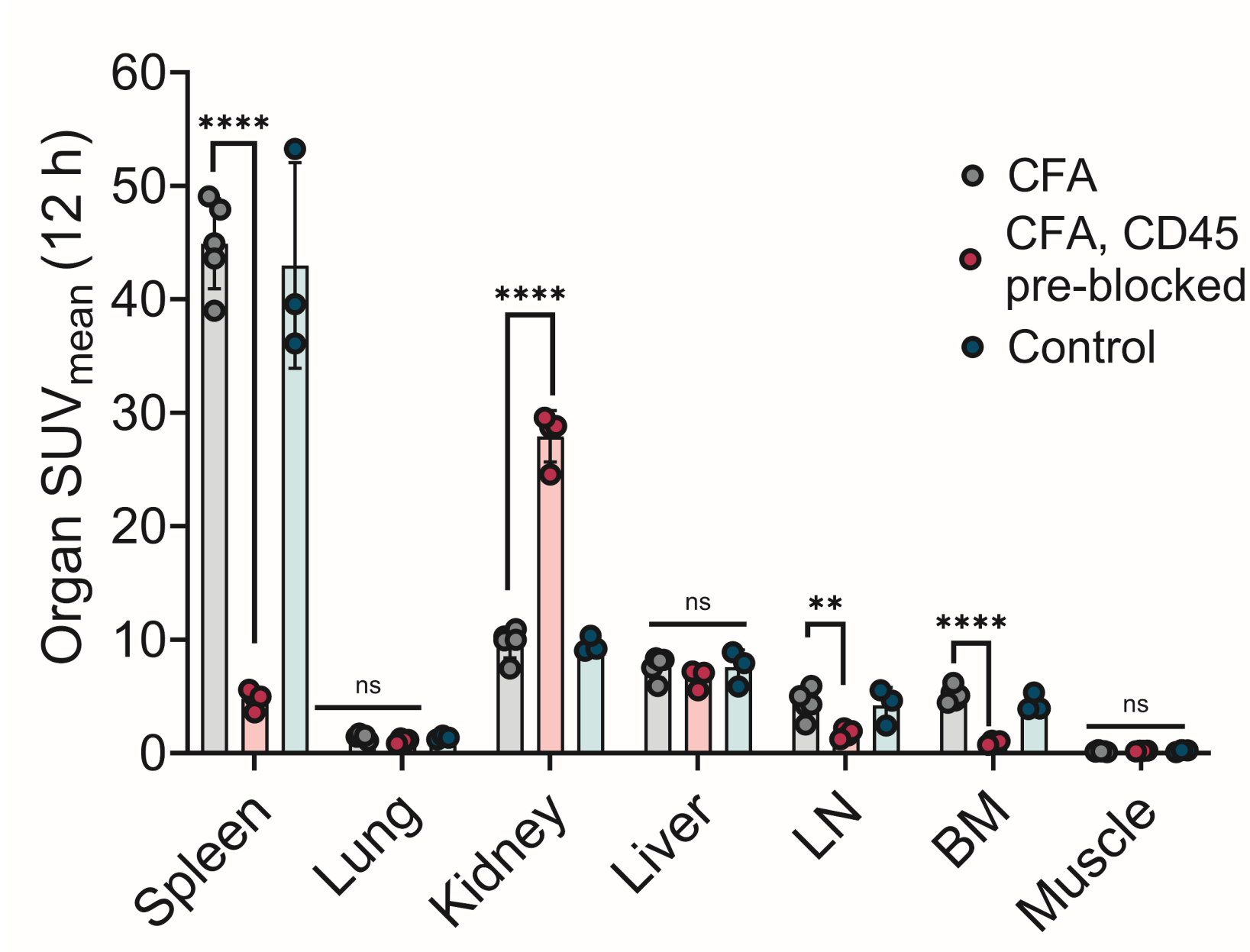

Figure S26: Comparison of organ-wise SUV_{mean} values between CFA-treated, CFA treated with CD45 pre-blocking, and control mice imaged with **^{89}Zr-N45-PEG10-DFO** at 12 h imaging time point. In the bar graphs, the bars show the SUV_{mean} and the error bars indicate the s.d. LN: lymph node; BM: bone marrow. Grey bar: CFA treated (n=5); pink bar: CFA treated with CD45 blocking (n=4); teal bar: healthy mice without CFA treatment serving as controls (n=3). Significance in the bar graph was tested using a Welch's t test and is indicated as *P< 0.05, **P< 0.01, and ***P<0.001.

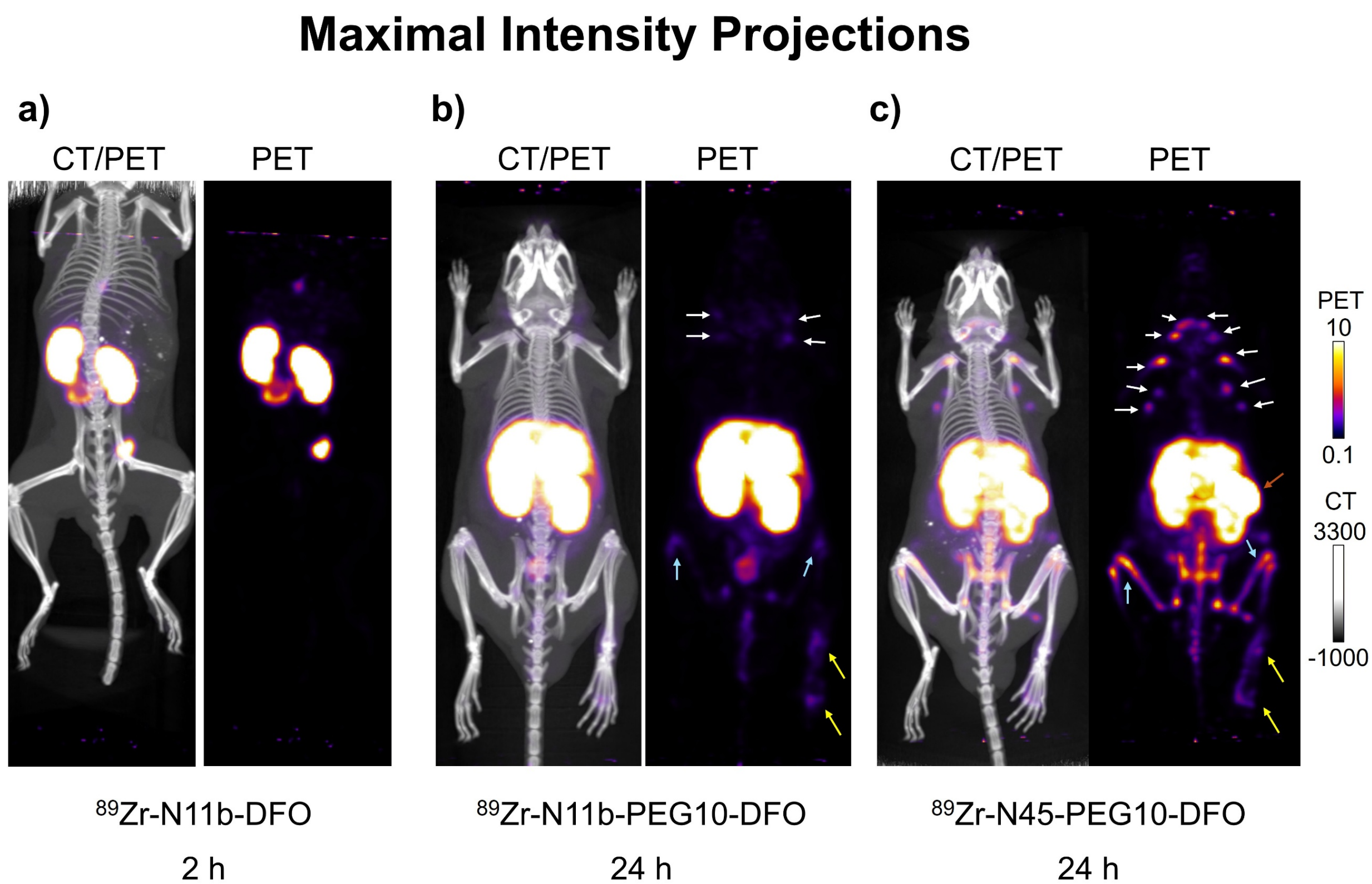

Figure S27: PET image comparison assessing the effect of PEGylation (**^{89}Zr-N11b-DFO** a) vs **^{89}Zr-N11b-PEG10-DFO** b)) and differential target binding between PEGylated nanobodies (**^{89}Zr-N11b-PEG10-DFO** b) vs **^{89}Zr-N45-PEG10-DFO** c)). The white arrows (lymph nodes), blue arrows (bone marrow), brown arrow (spleen), and yellow arrows (left foot pad inflammation). The PET and CT scale bars are in μCi mm^{-3} and Hounsfield units, respectively.

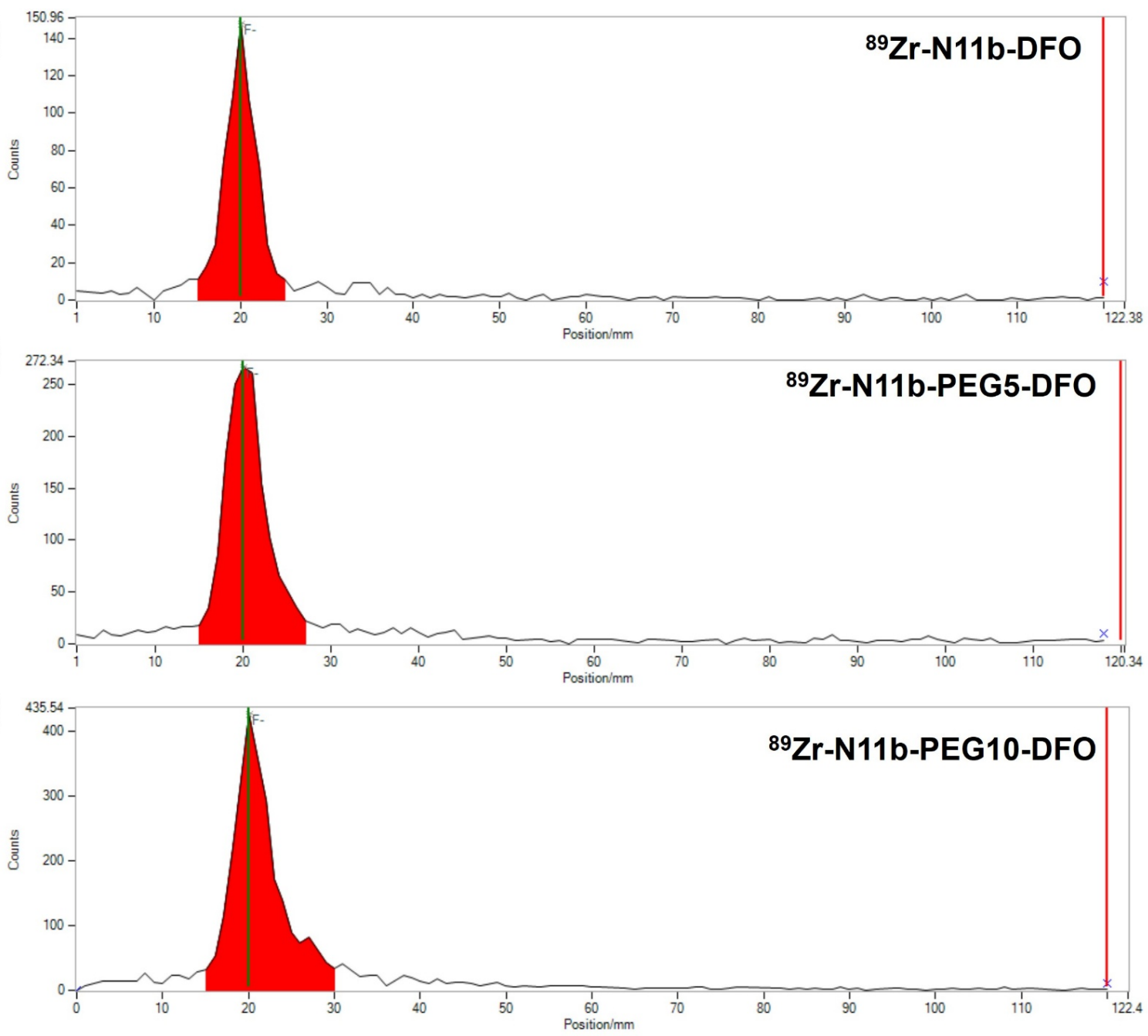

a)

Peak counts, %ROI	613, 100%
Unallocated counts	222
Peak % area	73.41%
R_f	0

b)

Peak counts, %ROI	1458, 100%
Unallocated counts	585
Peak % area	71.37%
R_f	0

c)

Peak counts, %ROI	1737, 100%
Unallocated counts	674
Peak % area	72.04%
R_f	0

Figure S28: Radio-thin-layer chromatogrphy (RadioTLC) of ^{89}Zr labeled N11b conjugates to determine radiochemical yield and purity. a) ^{89}Zr-N11b-DFO; b) ^{89}Zr-N11b-PEG5-DFO; c) ^{89}Zr-N11b-PEG10-DFO. The desired peak at the origin (R_f < 0.1) corresponds to the ^{89}Zr labeled nanobody conjugates with peak ROI 100% which means all the radioactivity in the plate is at that position.

Uncropped gel figures

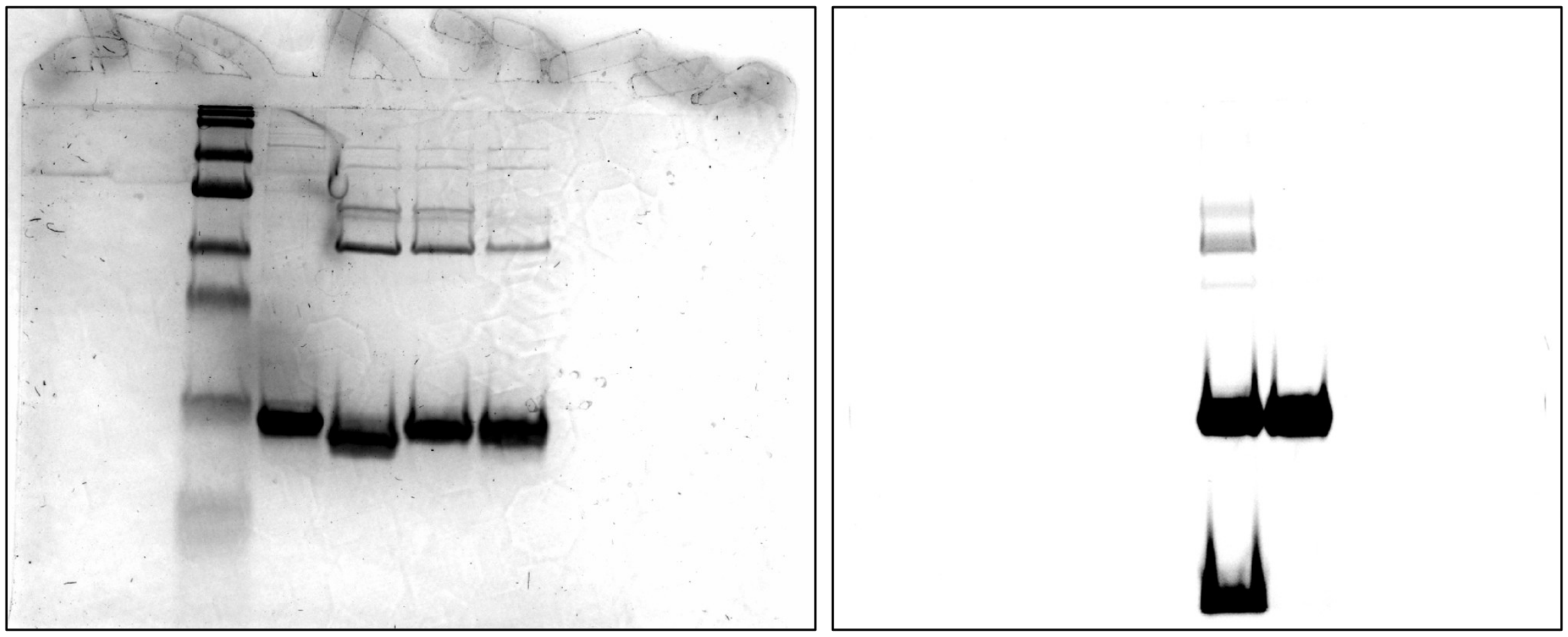

Uncropped gel for figure 2c. Left: Coomassie stained gel. Right: Fluorescence scan of the same gel.

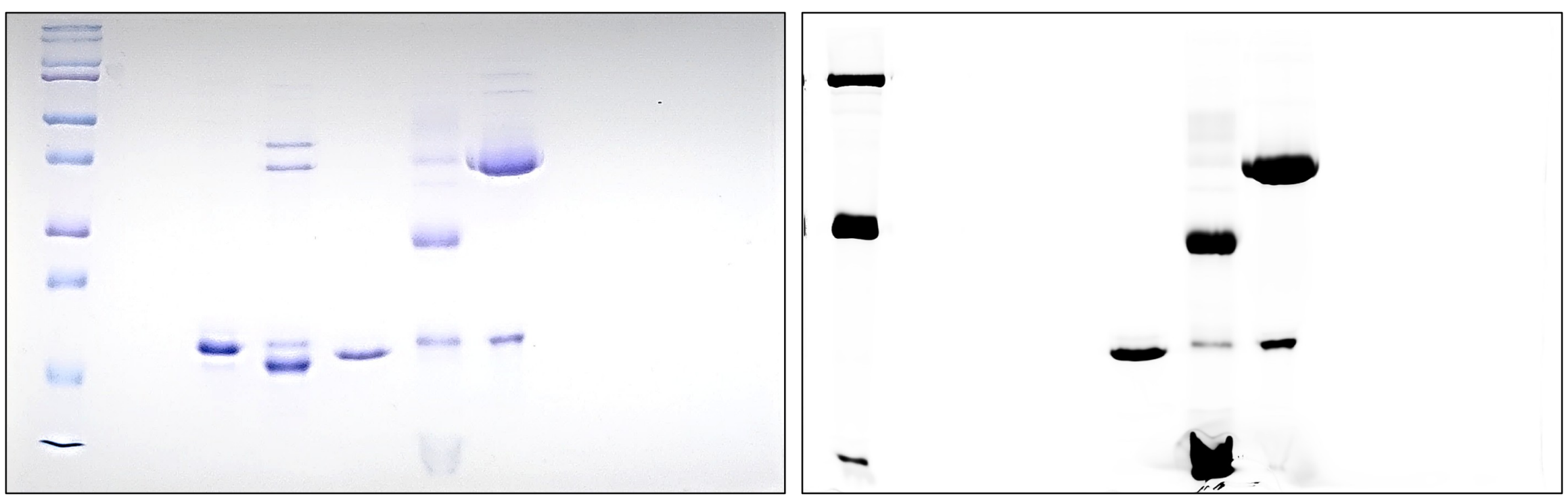

Uncropped gel for figure 3c. Left: Coomassie satined gel. Right: Fluorescence scan of the same gel.

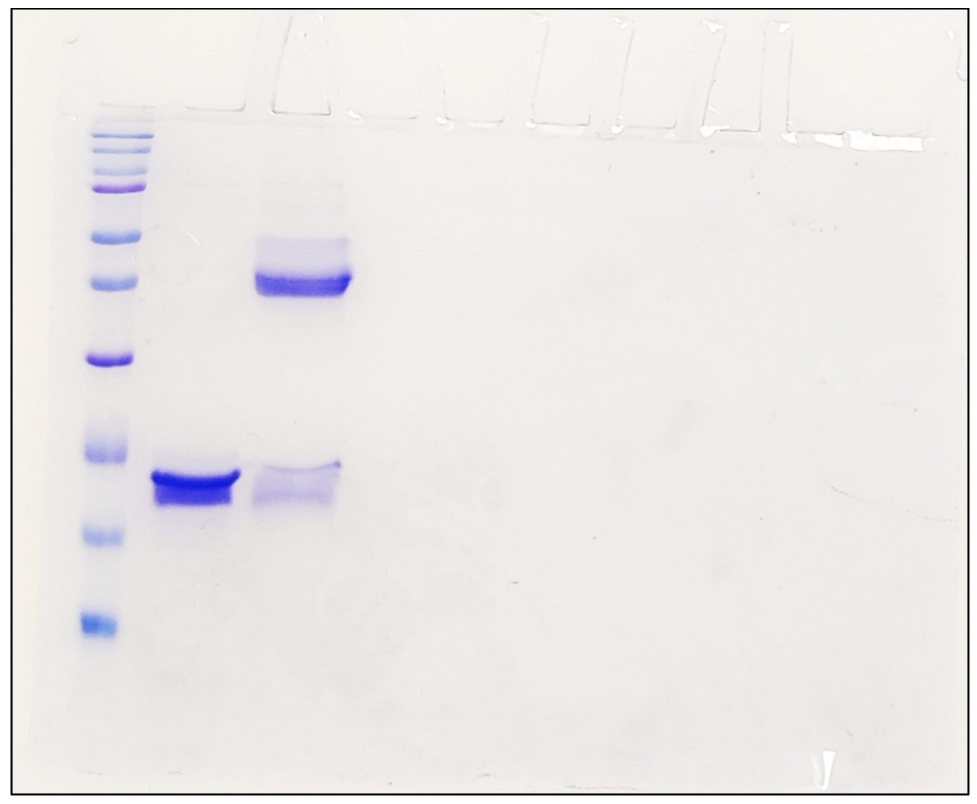

Uncropped gel for figure 5a.

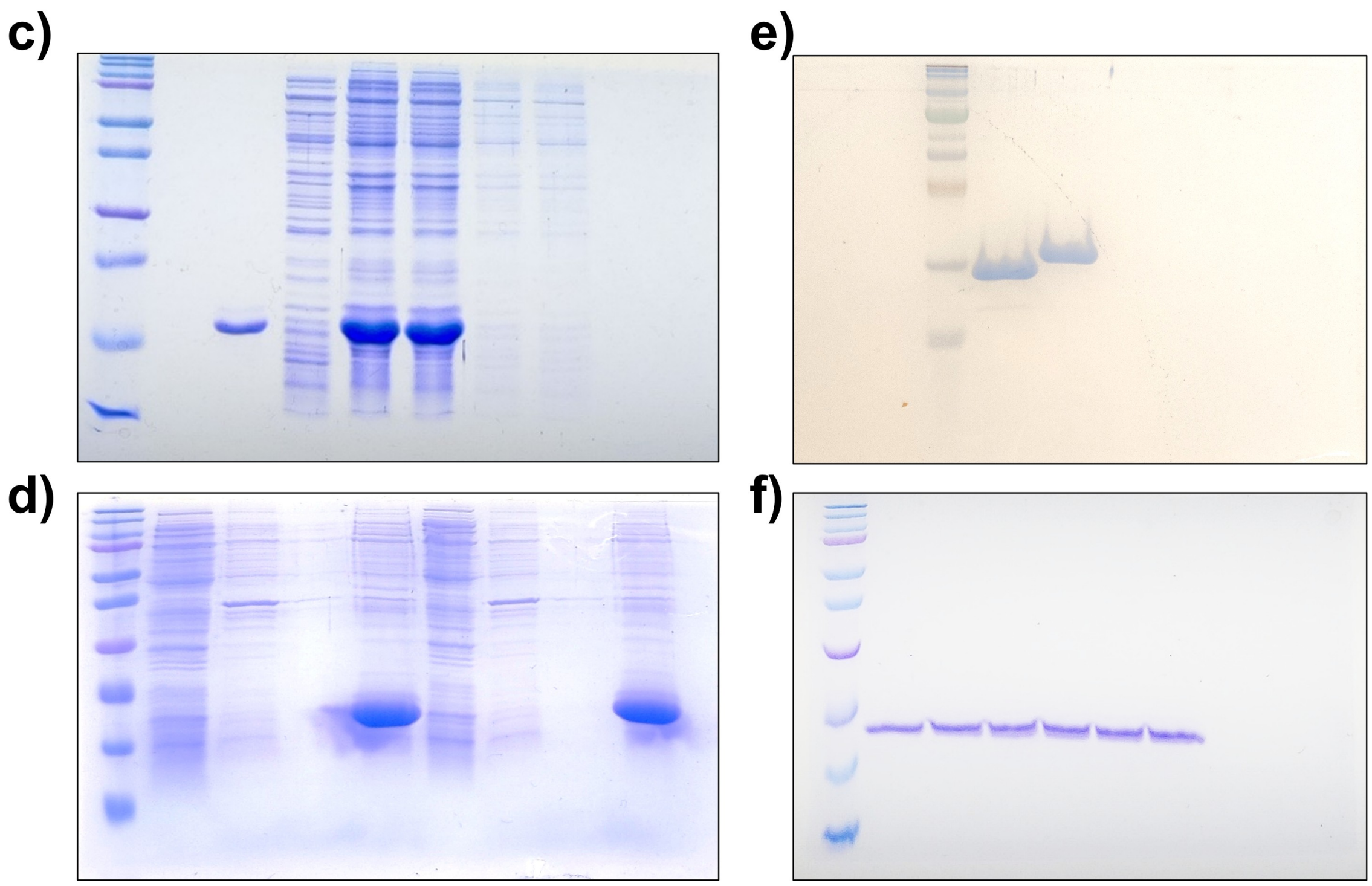

Uncropped gels for figure S1c-f.

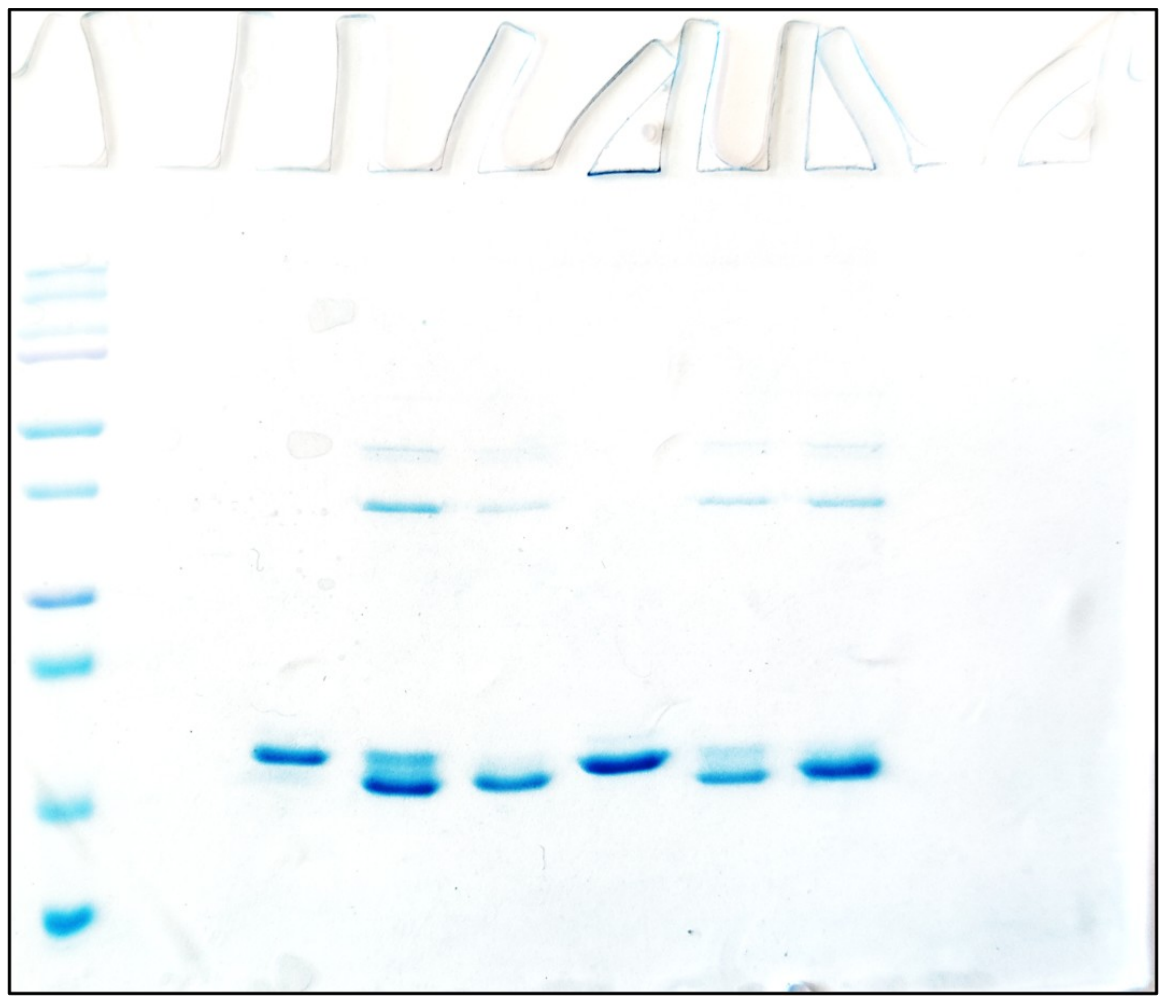

Uncropped gels for figure S6a.

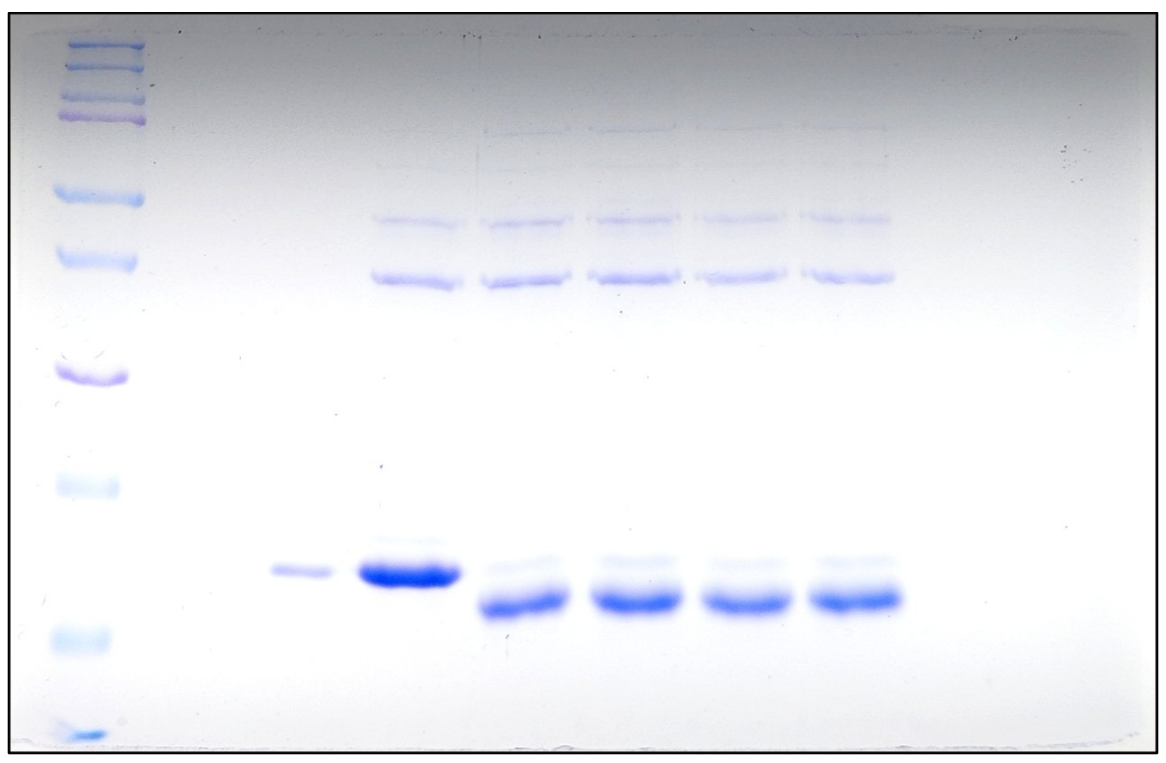

Uncropped gels for figure S9a.

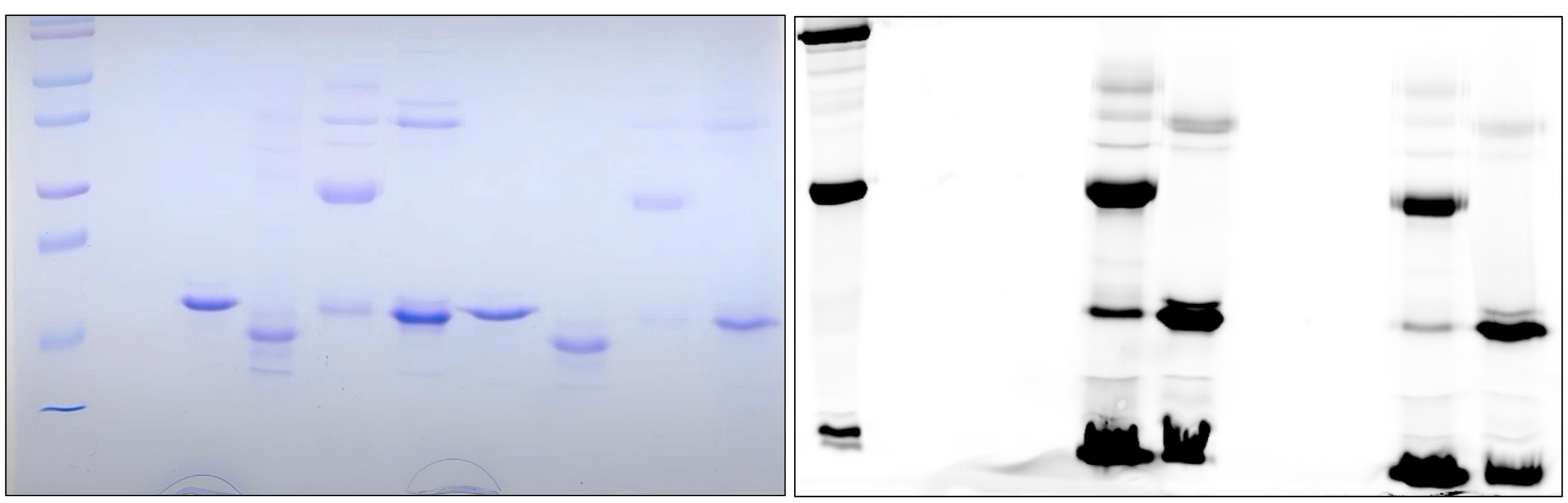

Uncropped gel for figure S10. Left: Coomassie stained gel. Right: Fluorescence scan of the same gel.

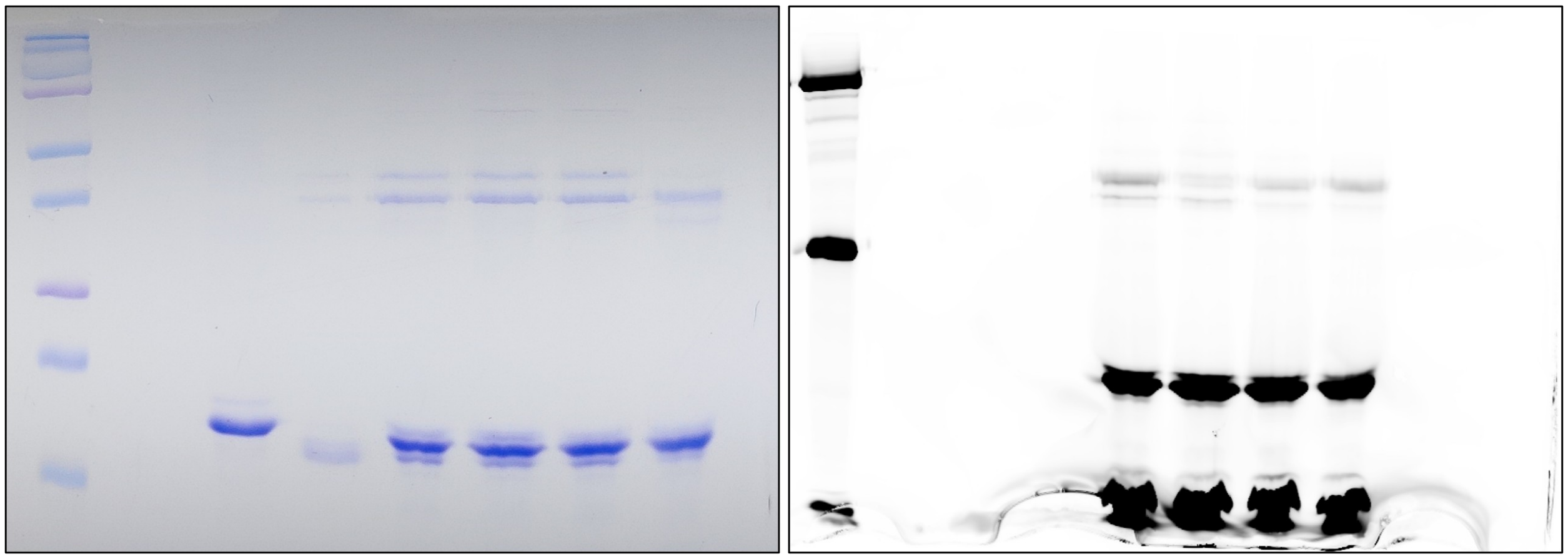

Uncropped gel for figure S11. Left: Coomassie stained gel. Right: Fluorescence scan of the same gel.

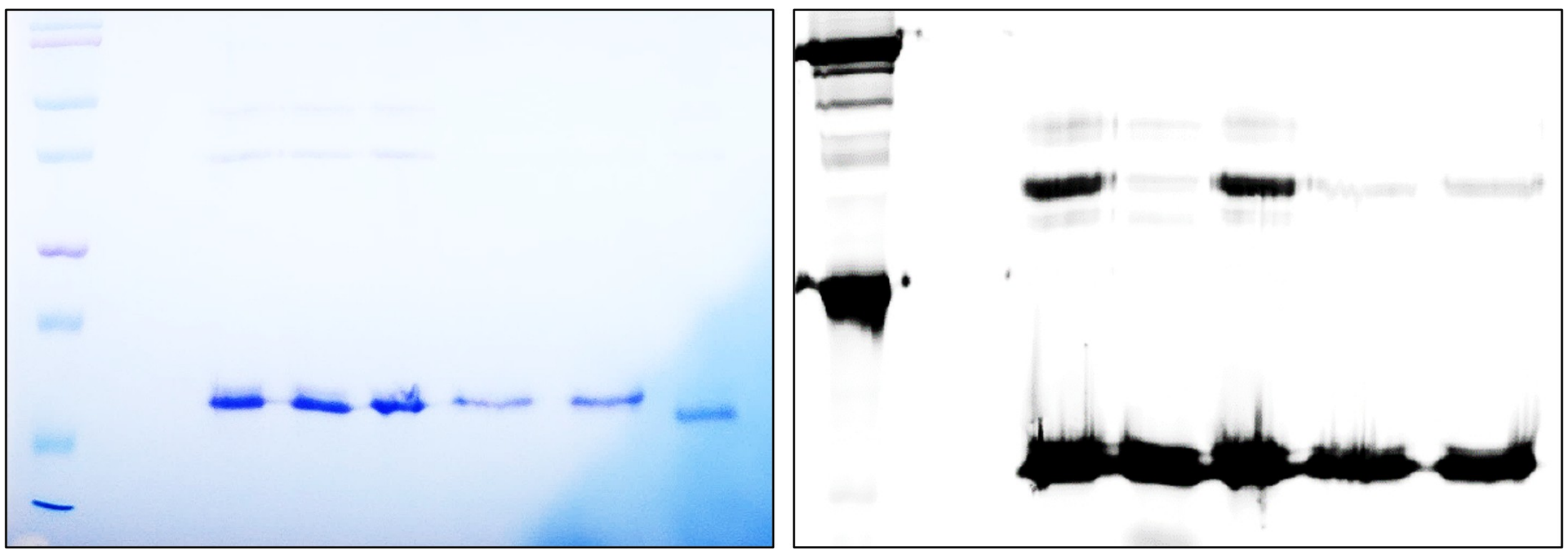

Uncropped gel for figure S12. Left: Coomassie stained gel. Right: Fluorescence scan of the same gel.

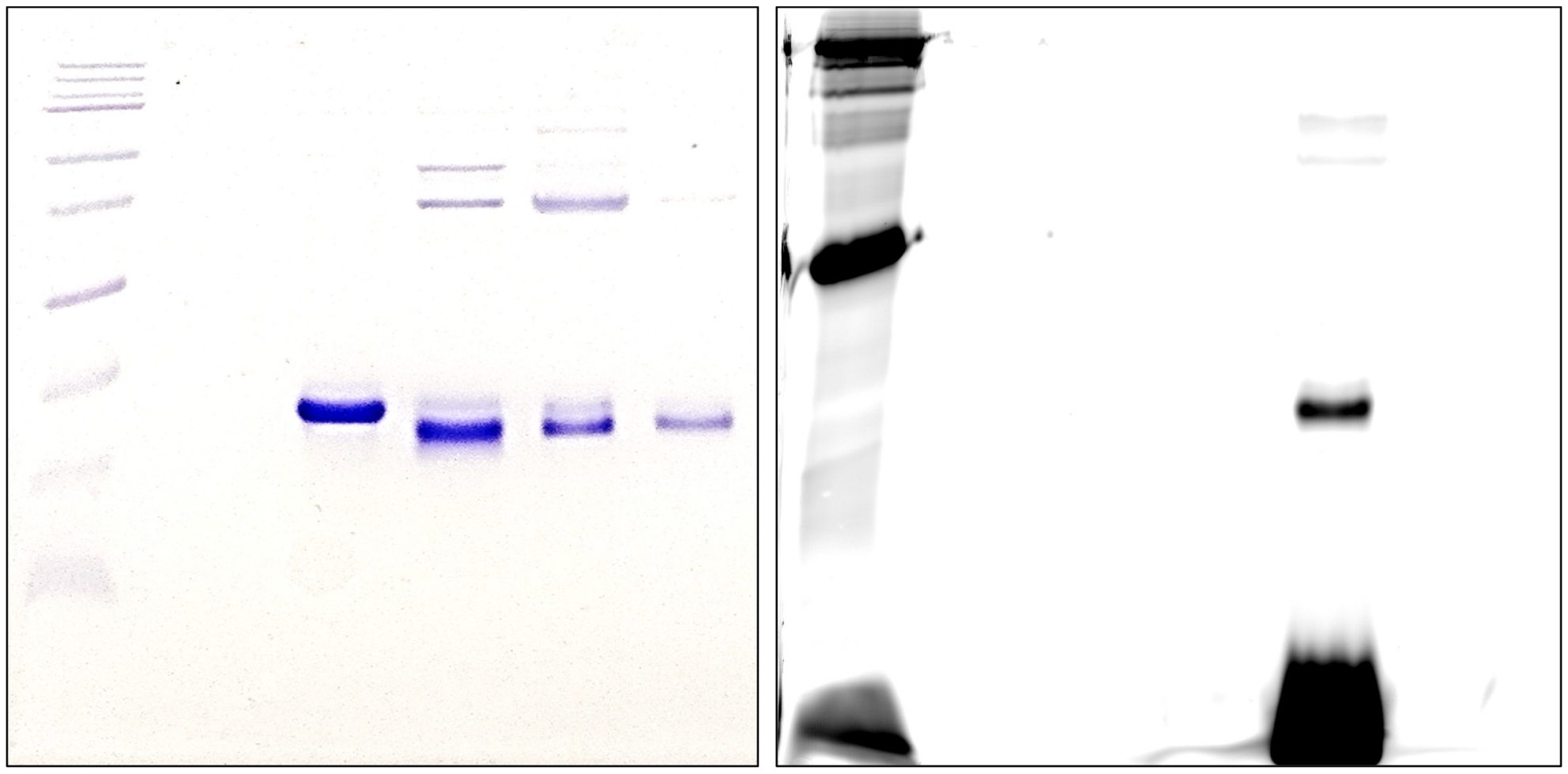

Uncropped gel for figure S13. Left: Coomassie stained gel. Right: Fluorescence scan of the same gel.

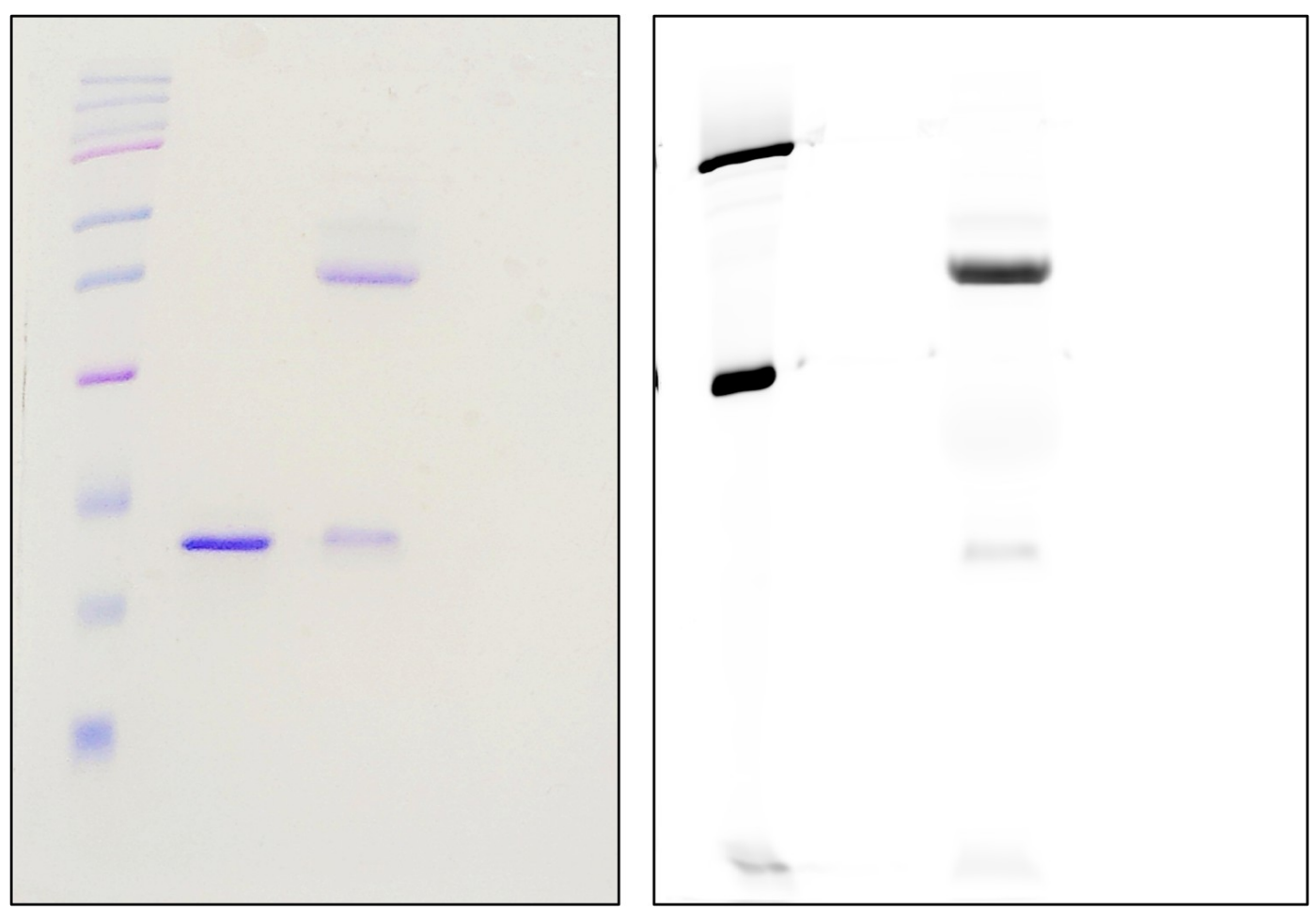

Uncropped gel for figure S23. Left: Coomassie stained gel. Right: Fluorescence scan of the same gel.

Experimental procedures

General: All synthetic reactions were carried out at rt and stirred magnetically unless otherwise noted. All water used in synthetic procedures was in-house deionized water. All reagents and solvents were purchased from commercial sources (Sigma Aldrich, Oakwood chemical, Fisher Scientific) and used directly without purification. Flash column chromatography was performed with silica gel (technical grade, pore size 60 Å, 230-400 mesh particle size, 40-63 μm particle size) purchased from Sigma Aldrich. TLC was performed on precoated (250 mm) silica gel 60 F254 plates purchased from Macherey-Nagel. Plates were visualized by staining with $KMnO_4$ or with a handheld UV lamp. Deuterated NMR solvents were purchased from Cambridge Isotope Laboratories, Inc. ^{1}H NMR spectra were obtained at 400 MHz; ^{13}C NMR spectra were obtained at 100 MHz. ^{31}P NMR was obtained at 162 MHz. Deuterated NMR solvents (CDCl3, D2O and DMSO-d6) were purchased from Cambridge Isotope Laboratories, Inc. Ammonium-d4 deuteroxide solution (25 wt. % in D2O, 99 atom % D) was purchased from Oakwood Chemical. All NMR spectra were acquired using a Bruker Advance III HD instrument with SampleXpress at 25 °C. Chemical shifts are reported in ppm and J values are in Hz. All spectra were referenced to the peak resulting from incomplete solvent deuteration except for ^{13}C NMR of diphosphate compounds which were obtained using D_2O as solvent. All reactions reported in this study were performed at least in duplicate to ensure reproducibility. For previously reported compounds, spectral data obtained were in good agreement with literature reports. Compound **1** was synthesized based on published protocols.[1] Geraniol and 3-Bromo-5-hydroxybenzaldehyde were purchased from commercial sources. HRMS spectra for synthetic reactions were obtained using a Bruker BioTOF II ESI/TOF-MS spectrometer or a Sciex X500 UPLC/QTOF-MS. Measured values are reported to 4 decimal places and are within 5 ppm of the calculated values. All calculated mass values are based on the most abundant isotope. Protein LC/MS analyses were performed using either a Thermo Scientific Elite Orbitrap Hybrid mass spectrometer or a Thermo Scientific LTQ XL™ Linear Ion Trap mass spectrometer, both equipped with a Zorbax 300 SB-C3 rapid-resolution HD column (1.8 μm, 2.1 × 100 mm). Raw data were processed using Thermo Scientific Protein Deconvolution software (version 2.0) or ProMassXcali software to generate deconvoluted protein masses. Preparative HPLC separations were performed using an Agilent 1100 series system (ChemStation Software, G1312A binary pump, G1329A autosampler, G1315B diode array detector, Telodyne Foxy R1 fraction collector), equipped with an Agilent ZORBAX 300SB-C18, 5 μm, 9.4 x 250 mm column. Ultrafiltration of protein samples (3-15 mL) was performed using a Beckman Coulter Avanti JXN-series centrifuge equipped with JA-25.5 rotor. For smaller protein volumes (500 μL-3 mL), ultrafiltration was performed using a Thermo Scientific ventilated benchtop accuSpin Micro 17 (Cat. 13-100-675), with samples briefly placed on ice after each centrifugation cycle to prevent protein degradation. Except for the final protein storage step prior to radiolabeling and PET imaging, where 50 mM HEPES buffer (pH 7.4) was used, all other protein reactions were performed in 100 mM phosphate buffer (prepared from NaH_2PO_4 and Na_2HPO_4 to achieve the desired pH). Protein desalting and buffer exchange were performed using disposable PD-10 desalting columns (Cytiva, Cat. 17085101) packed with Sephadex™ G-25 resin. Each column contains an 8.3 mL bed volume with dimensions of 14.5 cm × 1.5 cm (length × inner diameter). A 2.5 mL protein sample was loaded onto the column, and proteins were eluted

in 3.5 mL of buffer, effectively separating them from low-molecular-weight components including salts and urea. Columns were operated at 4 °C according to the manufacturer's instructions to maintain protein stability. TAMRA-DBCO was purchased from BroadPharm (Cat. BP-22456), and DFO-DBCO was obtained from Macrocyclics (Cat. B-773). All water used for protein buffers was obtained from a Milli-Q purification system (18.2 mΩ·cm). Buffers were pre-treated with Chelex-100 resin (Millipore Sigma, Cat. C7901) overnight at 4 °C to remove metal ion impurities.

Cell culture and maintenance: RAW 264.7 cells were obtained from ATCC and were verified by STR analysis through ATCC. Unless otherwise stated, cells were maintained and cultured for experiments in high glucose DMEM (Gibco 21068028) supplemented with 10% FBS (Gibco A5670701) and 1% penicillin-streptomycin (hereafter referred to as "DMEM"), at 37 °C in a humidified incubator with 5% CO_2 atmosphere. All PBS used is Dulbecco's phosphate buffered saline without $CaCl_2$ or $MgCl_2$ (Gibco 14190144). For confocal imaging, Nunc™ 35 mm tissue culture imaging dishes (Thermo Fisher Scientific, Cat. No. 150318) were used and the cells were maintained in FluoroBrite™ DMEM (Gibco, A1896701) following the final step of the staining workflow.

General considerations for PET imaging: All animal studies were conducted in strict accordance with ethical and safety protocols approved by the Dana-Farber Cancer Institute Committee on Animal Care (protocol 20-006) and the Boston Children's Hospital Committee on Animal Care (protocol BCH 00002392). Healthy female C57BL/6 mice, aged 5 to 6 weeks, were purchased from Jackson Laboratory (#000664). ^{89}Zr was obtained from the Mallinckrodt Institute of Radiology at Washington University in St. Louis as a stock solution in 1.0 M oxalic acid. Additional chemical reagents, including HEPES buffer, sodium carbonate, and phosphate-buffered saline, were purchased from Sigma-Aldrich and Thermo Fisher Scientific, while PD-10 size-exclusion cartridges were obtained from GE Healthcare. In vivo PET/CT imaging was performed using a G8 PET/CT scanner (PerkinElmer). Image analysis and PET quantification were conducted using VivoQuant software and Imalytics software, where regions of interest (ROIs) were manually delineated around the organs of interest based on CT image.

General considerations for gel imaging: Unless otherwise stated, desalted protein conjugates were mixed with 4x Laemmli sample buffer containing 50% 0.5 M DTT and analyzed by SDS-PAGE on 15% gels. Electrophoresis was performed at 120 V for 120 min or until the tracking dyes had completely migrated out of the gel. TAMRA fluorescence was imaged on a Typhoon FLA 9500 (GE Healthcare) using a 520 nm LED and 605 nm emission filter (laser exposure setting 550), followed by Coomassie Brilliant Blue staining to visualize total protein. ImageJ was used to adjust global contrast and brightness of gel images and to quantify band intensities for both fluorescence and total protein staining.

Docking: To model compound **1** and **2** in the active site of FTase (PDB file 1JCR), docking was performed using Glide (version 5.5, Schrodinger). The FTase crystal structure was prepared using the default settings in the protein preparation wizard as part of the Maestro 9.0 package. A receptor grid large enough to encompass the entire binding site for compound **1** and **2** was generated from the prepared FTase enzyme. A standard precision docking parameter was set,

and 100 ligand poses per docking were generated. The 20 top poses with the lowest docking scores were then further subjected to post docking minimization. The conformations with overall lowest energy were chosen for display for each probe. The Glide (version 5.5) and Maestro (version 9.0) software used in docking studies was licensed from Schrodinger, LLC (New York).

Design and expression of the anti-murine CD11b and CD45 nanobodies (N11b and N45): The **N11b** and **N45** nanobody sequences were engineered with an N-terminal His_6 tag for purification and a C-terminal Flag tag (DYKDDDDK) positioned upstream of the FTase recognition motif (CVIA). The constructs were cloned into the pET22b vector (GenScript) and expressed in E. coli BL21(DE3) as insoluble proteins. Functional nanobodies were obtained by refolding, following a modified version of an established protocol described below.

Protein sequence:

1. **N11b**:
HHHHHHGSQVQLQESGGGLVQAGGSHNLSCTASGITFSSLAMGWFRQTPGKEREFVANI
MRSGSSVFYADSVRGRFTISRDNAKNTAHLQMNSLKPEDTAVYFCAATRGAWPAEYWGQ
GTQVTVSSDYKDDDDKSGCVIA

2. **N45**:
HHHHHHGSQVQLVESGGGLVHPGDSLRLSCAASGSVFNSATMGWYRQSPGSQRELVATI
VVGTPTYADSVKGRFTISRDNAKNIVYLQMNSLKPEDTAVYYCNYRATYTSGYSRDYWGQ
GTQVTVSSDYKDDDDKSGCVIA

I) Expression of N11b and N45

LB agar plates were prepared with kanamycin (50 µg/mL, final concentration). A frozen bacterial stock was thawed, and 2 µL of the stock was spread onto the plate using a sterile cell spreader. The plate was incubated overnight at 37 °C. A single colony was then picked with a sterile pipette tip and used to inoculate 100 mL of autoclaved LB media supplemented with kanamycin (0.1 mL of 50 mg/mL stock solution, final concentration of 50 µg/mL) in a 500 mL Erlenmeyer flask. The culture was incubated overnight at 37 °C with shaking at 225 rpm. Next, 1 L of the growth medium was prepared with 20 g BactoTryptone, 15 g BactoYeast extract, 8 g NaCl, 1.3 g Na_2HPO_4 and 1.0 g KH_2PO_4 and autoclaved. Just before inoculation, kanamycin (1 mL of 50 mg/mL stock solution, final concentration of 50 µg/mL), chloramphenicol (1 mL of 25 mg/mL stock solution in ethanol, final concentration of 25 ug/mL, final concentration) and 20 mL/L of 20% (w/v) sterile-filtered glucose solution. Starter culture was added to the growth medium in a 4 L flask to achieve an OD_{600} of 0.1 (about 30 mL of starter culture per liter). The culture was incubated at 37 °C with shaking at 225 rpm until the OD_{600} reached 0.6-0.8, at which point isopropyl-β-d-1-thiogalactopyranoside (IPTG) was added to a final concentration of 0.5 mM to induce expression. The culture was then incubated for an additional 3.5 h.

II) Isolation of N11b and N45 from inclusion bodies

The induced cells were harvested at 6000 x g for 15 min at 4 °C. The pellets were resuspended completely in the resuspension buffer (50 mM Tris•HCl, pH = 8.0, 20 mM $MgCl_2$, 0.02% g/L NaN_3;

10-15 mL for cells from harvested from 1 L of media). Then, PMSF (1 mM final concentration from 100 mM stock in ethanol) and lysozyme (0.1 mg/mL final concentration from 10 mg/mL stock in water) were freshly added to the resuspended cells and incubated on the ice for 30 min. Cells were lysed using a Fisher Scientific 60 Sonic Dismembrator. The resuspended cells were sonicated for 10 min (10 s on, 10 s off cycles, power level 7-8) while kept on ice, and the lysate was clarified by centrifugation at 17,000 x g for 20 min at 4 °C. If the lysate was chunky after sonication, the resulting pellet was again resuspended in 10 mL fresh resuspension buffer and lysis process was repeated. After centrifugation, the supernatant was decanted, and the pellet was first washed two times with 20 mL of Tris-HCl (50 mM, pH = 8) buffer containing 0.5 % Triton X-100 and 1 M urea and then washed three times with 20 mL of Tris-HCl (50 mM, pH = 8.0) buffer. To resuspend the pellet completely in the buffer for each wash, it was sonicated briefly 50 s (10 s on, 10 s off cycles, power level 7-8). After each wash, the inclusion bodies were recovered by centrifugation at 17,000 x g for 20 min at 4°C and the supernatant discarded. The resulting inclusion bodies were solubilized in 20 mL of solubilization buffer (50 mM Tris•HCl, pH 8.0 containing urea (8 M) and 2-mercaptoethanol (2 mM)) and incubated on a rocker at 4 °C overnight followed by filtration through a 0.45 μm filter to remove any insoluble material. The concentration of the solubilized inclusion body proteins was determined by measuring A_{280} using a Nanodrop instrument, calibrated with a series of BSA standards prepared in solubilization buffer. The purified proteins were analyzed by 15% SDS-PAGE.

III) Refolding of purified N11b and N45

Refolding of the nanobodies was carried out using a dilution refolding method. The denatured proteins were first diluted to a concentration of 0.1-0.2 mg/mL using a buffer containing Tris•HCl (50 mM, pH 8.0), urea (8 M) and 2-mercaptoethanol (2 mM). Refolding buffer containing Tris•HCl, (50 mM, pH = 8.0), and L-arginine (0.4 M) was prepared and cooled to 4°C. A mixture of oxidized glutathione (2 mM final concentration) and reduced glutathione (10 mM final concentration) was added to the refolding solution just before adding the denatured protein. A solution of diluted protein (100 mL, precooled to 4 °C) was slowly added to the beaker at a rate of 2.5 mL/min using a Pharmacia Biotech P-1 peristaltic pump (Cat. No. RBN 22687) and incubated at 4 °C for 16 h with continuous stirring. The resulting solution was centrifuged at 15,000 x g for 15 min at 4 °C to remove any insoluble material. The resulting supernatant then was vacuum filtered through a 0.45 μm PES membrane filter and then concentrated down to 100 mL with a Millipore ultrafiltration stirred cell (10 kDa MWCO membrane) in the cold room. Pre-cooled Tris•HCl (50 mM, pH = 8) was added to the solution which was then again concentrated down to 100 mL. One liter of pre-cooled 50 mM Tris-HCl buffer (pH 8.0) was added to the solution in four successive dilutions, followed by reconcentration to 100 mL to remove urea. The resulting protein solution was then purified using Ni-NTA affinity chromatography, followed by treatment with 50 mM DTT (to reduce any disulfide-mediated dimerization and glutathione adducts) and dialysis against metal-free HEPES buffer (50 mM, pH 7.5) containing NaCl (150 mM) (1 L prepared by treatment with 10 g of Chelex 100 resin (Na form) at 4 °C for 16 h), using Thermo Scientific SnakeSkin™ dialysis tubing (10 kDa MWCO, Cat: 88243). The protein concentration was determined using Pierce™ BCA Protein Assay Kits (Thermo Scientific, Cat: 23225). The protein was stored in chelex-treated

buffer containing HEPES (50 mM, pH 7.5), NaCl (150 mM) and glycerol (10% v/v) at -80 °C until further use.

Nanobody refolding optimization and functional validation by flow cytometry: Optimal refolding conditions for the nanobody were identified by systematically varying oxidative refolding parameters, including the ratio of reduced and oxidized glutathione (GSH/GSSG) and the presence or absence of β-mercaptoethanol (2-ME) in the resolubilization buffer. The conditions tested included no 2-ME with 1 mM GSSG and 5 mM GSH; with 2-ME and 1 mM GSSG with 1 mM GSH; with 2-ME and 5 mM GSSG with 5 mM GSH; with 2-ME and 1 mM GSSG with 5 mM GSH; no 2-ME with 5 mM GSSG and 5 mM GSH; and with 2-ME and 2 mM GSSG with 10 mM GSH. The functionality of refolded nanobody preparations obtained under each condition was assessed by flow cytometry using RAW 264.7 macrophage cells. Cells were incubated with refolded nanobody (~100 nM final concentration, used directly from the refolding mixture) for 30 min at 4 °C. Cells were then washed twice with DPBS (600 μL, 300 × g, 5 min) and stained with anti-His APC antibody (1 μg/mL final concentration in 100 μL; BioLegend, Cat. No. 362605) for 30 min at 4 °C. Following two additional washes with DPBS (600 μL, 300 × g, 5 min), samples were analyzed by flow cytometry. As a positive control, cells were stained with 100 nM periplasmic expressed purified anti-CD11b nanobody lacking the CVIA tag, followed by anti-His APC staining. As a negative control, cells were incubated with anti-His APC alone in the absence of nanobody. Data was acquired on a Cytek Aurora (SpectroFlo), with 1×10^4 events collected per sample. APC fluorescence was excited using a 650 nm laser and detected with a 660/20 nm bandpass filter.

Design and periplasmic expression of the anti-mouse CD11b and CD45 nanobodies (N11bp and N45p)

The N45p nanobody sequence was engineered with a C-terminal Flag tag (DYKDDDDK) and a His_6 tag, whereas N11bp contains only a C-terminal His_6 tag to enable protein purification. It was then cloned into the pHEN6 vector (GenScript). The nanobody was expressed in the WK6 Escherichia coli strain using periplasmic expression, following established protocols. In brief, the starter culture was diluted in 2 L of terrific broth medium with 2 ml ampicillin (100 μg ml^{-1}), and the optical density at 600 nm was adjusted to 0.1. On reaching an optical density at 600 nm of 0.5-0.8 after incubation at 37 °C, IPTG (1 mM) was added to induce nanobody expression and the culture was maintained overnight at 20 °C. After expression, cells were collected by centrifugation at 12,000g for 5 min. The resulting pellet was resuspended in 30 ml TES (0.2 M Tris, 600 μM EDTA and 0.5 M sucrose, pH 8) buffer and incubated at 4 °C for 2 h. Subsequently, Tris buffer (50 mM Tris, pH 8, fourfold dilution) was added to lyse the cells using osmotic shock. Centrifugation at 14,000 x g for 15 min was performed to separate the supernatant. The nanobody was then purified from the supernatant using Ni-NTA beads.

Head-to-head comparison of binding affinities of purified periplasmically expressed nanobodies (N11bp and N45p) and purified refolded nanobodies (N11b and N45). RAW 264.7 macrophages were cultured in a 20 cm tissue culture dish to full confluence, harvested by scraping, and transferred to a 96-well U-bottom plate (CELLTREAT, Cat. No. 229190) at 1×10^5 cells per well. Cells were washed twice with PBSA (PBS containing 1% BSA) by centrifugation followed by removal of the supernatant. Purified nanobodies were prepared as 500 nM stock

solutions in PBSA and serially diluted sixfold to generate a concentration range of 83.3 nM to 0 nM. Each concentration was tested in triplicate for each nanobody construct. Cells were incubated with 100 μL of nanobody solution for 45 min on ice, followed by two washes with PBSA. Bound nanobodies were detected using anti-FLAG-PE (BioLegend, Cat. No. 637310) for N11b, N45, and N45p, and anti-His-AF647 (BioLegend, Cat. No. 362611) for N11bp, which lacks a FLAG tag. Detection antibodies were diluted 1:500 in PBSA, and 30 μL was added to each well for 30 min on ice. Cells were then washed twice with PBSA and the plate was analyzed on a BD Canto flow cytometer, with 10,000 cells recorded per well. Unstained cells were used as the negative control. Background fluorescence from the detection antibodies alone was assessed by incubating cells with the antibodies in the absence of prior nanobody incubation and was found to be negligible.

In vitro farnesyltrasferase-catalyzed nanobody labeling: Prior to prenylation, the protein was pre-incubated with 50 mM DTT on ice for 30 min to ensure complete thiol reduction. Prenylation reactions were generally performed in the presence of DTT; however, for substrate **2**, DTT was removed beforehand, as it reduces the aryl azide moiety to an aryl amine. DTT was removed by three rounds of buffer exchange with 100 mM phosphate buffer (pH 7.0) using an Amicon ultrafiltration centrifugal device (10 kDa MWCO, Cat: UFC9010), concentrating from 15 mL to 500 μL per cycle. Prenylation was carried out in phosphate buffer (100 mM, pH 7.0) containing KCl (20 mM), $MgCl_2$ (10 mM), $ZnCl_2$ (10 μM), Tween-20 (0.1% w/v), and glycerol (10%, v/v). To the reduced nanobody (5 μM final concentration in prenylation buffer), compound **1** or **2** (dissolved in 25 mM NH_4HCO_3) was added to a final concentration of 10 μM (**2**) or 30 μM (**1**), followed by yeast FTase (400 nM final concentration). Reactions were incubated in a water bath at 32 °C for 3 h. Excess isoprenoid analog was removed by three rounds of buffer exchange with phosphate buffer (100 mM, pH 6.0) using Amicon ultrafiltration centrifugal devices (10 kDa MWCO). For each round, 14.5 mL of phosphate buffer (100 mM, pH 6.0) was added and concentrated to 0.5 mL by centrifugation at 5000 x g for 30 min.

Metal-free click reaction between azide-functionalized nanobody and TAMRA-DBCO or DFO-DBCO: TAMRA-DBCO or DFO-DBCO (1 μL of a 50 mM stock solution in DMSO) was added to 99 μL of prenylated nanobody (100 μM) in phosphate buffer (PB, 100 mM, pH 7.0) and the mixture was incubated at rt for 1 h. SDS-PAGE and LC-MS analyses demonstrated were used to measure the conversion of **N11b-Az** to the clicked products **N11b-TAM** and **N11b-DFO**, with conversion efficiency quantified by densitometric analysis using ImageJ. An efficiency of ≥99% was obtained for both). LC/MS on the full-length protein was performed using a 4 min gradient from 20-100% solvent B (CH_3CN, 0.1% HCO_2H) in solvent A (H_2O, 0.1% HCO_2H) to analyze the product.

One-pot simultaneous coupling of nanobodies with TAMRA-DBCO or DFO-DBCO and aminooxy-PEG (5, 10, or 20 kDa): TAMRA-DBCO or DFO-DBCO (1 μL of a 50 mM stock in DMSO, 5 equiv) and aminooxy-mPEG (5, 10, or 20 kDa; NANOCS; 4 μL of a 12.5 mM stock in DMSO, 5 equiv) were added to 75 μL of prenylated nanobody (**N11b-AzAl** or **N45-AzAl**, 100 μM) in phosphate buffer (100 mM, pH 6.0). p-Phenylenediamine (pPDA; 20 μL of a 0.5 M stock in 100 mM phosphate buffer, pH 6.0) was added to a final catalyst concentration of 100 mM, and the reaction mixture was incubated for 15 h at rt. Reaction volumes were scaled up as needed for in vivo imaging studies. Products were desalted using PD-10 columns (8.3 mL bed volume; 14.5 cm

x 1.5 cm). A 2.5 mL reaction mixture was loaded onto each column, and proteins were eluted in 3.5 mL buffer to remove pPDA and exchange the samples into HEPES (50 mM, pH 7.5) containing 150 mM NaCl. SDS-PAGE (15% gel) was used to assess the efficiency of the click and oxime ligation reactions.

Sequential labeling of nanobodies with TAMRA-DBCO or DFO-DBCO followed by aminooxy-TCO: TAMRA-DBCO or DFO-DBCO (1 µL of a 50 mM stock in DMSO, 5 equiv) was added to 75 µL of prenylated nanobody (**N11b-AzAl** or **N45-AzAl**, 100 µM) in phosphate buffer (100 mM, pH 6.0) and incubated for 1 h at rt. Following this initial reaction, aminooxy-TCO (Conju-Probe, CP-6078; 5 µL of a 10 mM stock in DMSO, 5 equiv) and p-phenylenediamine (pPDA; 20 µL of a 0.5 M stock in 100 mM phosphate buffer, pH 6.0) were added to a final catalyst concentration of 100 mM, and the reaction was incubated for an additional 6 h at rt. Reaction scales were increased as needed for in vivo imaging studies. The final products were desalted using PD-10 columns by loading 2.5 mL of reaction mixture and eluting with 3.5 mL buffer to remove pPDA and exchange the protein into HEPES (50 mM, pH 7.5) containing 150 mM NaCl. Reaction efficiency was assessed by SDS-PAGE (15% gel).

Tetrazine ligation of TCO-modified intermediates with methyltetrazine-mPEG (10kDa): To perform the tetrazine ligation, methyltetrazine-mPEG (10 kDa; 4 µL of a 5 mM aqueous stock; Vector Labs, CCT-1091) was added to the TCO-modified nanobody (**N11b-TAM-TCO, N45-TAM-TCO, N11b-DFO-TCO,** or **N45-DFO-TCO**; 96 µL of a 100 µM stock in 50 mM HEPES, pH 7.5, containing 150 mM NaCl) at 2 equiv relative to the protein. The reaction was incubated at rt for 30 min followed by desalting using a PD-10 prepacked column and exchange the protein into HEPES (50 mM, pH 7.5) containing 150 mM NaCl. The reaction was scaled up as needed for in vivo imaging studies. SDS-PAGE (15% gel) was used to assess the efficiency of the tetrazine ligation.

One-pot simultaneous coupling of nanobodies with aminooxy-TAMRA and DBCO-mPEG (20 kDa): TAMRA-aminooxy (1 µL of a 50 mM stock in DMSO, 5 equiv) and DBCO-mPEG (20 kDa; 5 µL of a 10 mM stock in DMSO, 5 equiv) were added to 74 µL of prenylated nanobody (N11b-AzAl or N45-AzAl, 100 µM) in phosphate buffer (100 mM, pH 6.0). p-Phenylenediamine (pPDA; 20 µL of a 0.5 M stock in 100 mM phosphate buffer, pH 6.0) was added to a final catalyst concentration of 100 mM, and the reaction mixture was incubated for 6 h at rt. Reaction volumes were scaled up as needed. Products were desalted using NAP-5 columns (Cytiva illustra, 5 mL bed volume). A 100 µL reaction mixture was loaded onto each column, and proteins were eluted in 2 mL buffer to remove pPDA and aminooxy-TAMRA exchange the samples into PBS (pH 7.4). SDS-PAGE (15% gel) was used to assess the efficiency of the click and oxime ligation reactions.

Flow cytometry-based affinity titration of TAMRA-labeled nanobodies. Affinity titrations were performed to determine the binding of TAMRA-labeled nanobody conjugates (**N11b-TAM**, **N11b-PEG5-TAM, N11b-PEG10-TAM, N11b-PEG20-TAM, N45-PEG10-TAM,** and **N45-PEG20-TAM**) to CD11b and CD45 epitopes on RAW 264.7 macrophages. For **N11b-PEG20-TAM** and **N45-PEG20-TAM**, the assay was performed in a 96-well plate format, whereas all other constructs were analyzed in tube format. In both formats, $1{\times}10^5$ cells were incubated with TAMRA-labeled

nanobodies in PBSA (PBS containing 1 mg/mL BSA) for 45 min at 4 °C in the dark. Nanobodies were tested over a concentration range of 0-500 nM. After incubation, cells were washed twice with PBSA (tube format: 600 μL, 300 × g, 5 min; plate format: 200 μL, 600 × g, 3 min). No secondary staining was required. For tube-based assays, samples were analyzed on a BD LSRFortessa, and TAMRA fluorescence was detected using the 561 nm laser with a 581/15 nm bandpass filter; $1×10^4$ cells were collected per sample. For plate-based assays, data were acquired on a Cytek Aurora using the 561 nm yellow-green laser and YG1 channel (577/20 nm), with $1×10^4$ cells recorded per sample.

Affinity titration data analysis: Data analysis was performed with FlowJo v10.10 software. The K_D values were calculated using Origin 2023 by fitting the data to a one site binding equation, $Y = F_{max}•X/(K_D + X)$, where Y is the mean fluorescence intensity value measured at a specific nanobody concentration, F_{max} is the maximum mean fluorescence intensity, X is the nanobody concentration and K_D is dissociation constant. All K_D values derived include the standard error reported for these curve fits. Experiments were conducted in triplicate and data are presented as the mean ± standard deviation of three independent trials. For TAMRA-labeled constructs, the MFI of the negative control (nanobody without TAMRA) was subtracted from each sample MFI prior to analysis. For PE, APC and AF647 antibody-labeled experiments, the MFI values were corrected by subtracting both the negative control signal and the background fluorescence arising from non-specific antibody binding. MFI data were converted to "Fraction Bound" values by dividing individual MFI values by the F_{max} value determined for the specific data set. Plots of "Fraction Bound" versus [Nanobody] were created for the **N11b** and **N45** conjugates using these processed data.

Anti-FLAG affinity purification of nanobodies: Anti-DYKDDDDK G1 affinity resin (GenScript, Cat. No. L00432S; binding capacity: 1.5 mg protein per mL settled resin) was used for purification according to the manufacturer's binding capacity. Nanobody samples were diluted in five resin-bed volumes of buffer relative to the settled resin volume that is being used, and the resin was then added directly to the protein solution. Binding was allowed to proceed for 2 h at room temperature with gentle mixing of the tube. The resin was collected by centrifugation (6000 × g, 45 s) and washed with PBS. Bound nanobody was eluted using 1.75 M NaSCN, and the eluate was subsequently buffer-exchanged into the desired buffer using Amicon ultrafiltration units.

Size-exclusion chromatographic purification of labeled nanobodies: Size-exclusion chromatography (SEC) was performed on an ÄKTA pure FPLC system equipped with a Superdex 75 Increase 10/300 GL column (Cytiva). Samples were loaded using a 1 mL sample loop, with injection volumes of 100-200 μL containing approximately 200 μg of protein per run. PBS was used as the mobile phase, and separations were carried out at a flow rate of 0.4 mL/min over 1.5 column volumes. Under these conditions, protein recovery was typically 60-75%.

Confocal imaging of N11b-TAM binding to CD11b-expressing RAW 264.7 cells: RAW 264.7 cells (1×10^5) were seeded onto Nunc™ 35 mm tissue culture dishes and incubated for 24 h at 37 °C. Cells were then incubated with 150 nM **N11b-TAM** (300 μL) at 4 °C for 45 min, washed three times with 1 mL of staining buffer (0.5% BSA in DPBS containing 0.1% NaN_3) for 1 min

each, and stained with anti-His PE/Cy5 antibody (1 µg /mL, 300 µL, Biolegend, Cat. 362635), recognizing the N-terminal His-tag of **N11b-TAM**, for 30 min at 4 °C. After washing with DPBS (3 x 1 mL, 1 min each), nuclei were stained with Hoechst 34580 (1 µg/ mL, 300 µL, 10 min at rt), followed by three additional PBS washes (1 mL, 1 min each). Finally, cells were maintained in FluoroBrite™ DMEM for imaging. Confocal images were acquired on a Nikon AX R inverted confocal microscope using the 405 nm excitation laser for Hoechst (420-551 nm emission), the 561 nm excitation laser for TAMRA (571-625 nm emission) and PE/Cy5 (655-850 nm emission), at 2048 x 2048 pixel resolution with a 60x oil-immersion objective in Galvano scan mode. Nuclei are shown in blue, TAMRA fluorescence in green, and PE/Cy5 in magenta; membrane colocalization appears as light pink. Scale bar = 10 µm. Images were processed and recolored using Fiji/ImageJ.

Confocal imaging of RAW 264.7 cells showing differential binding of N11b-PEG10-TAM and N45-PEG10-TAM: RAW 264.7 cells (0.3 x 10^6) were seeded onto Nunc™ 35 mm tissue culture imaging dishes and incubated for 24 h at 37 °C. Cells were then treated with 150 nM **N11b-PEG10-TAM**, **N45-PEG10-TAM**, or staining buffer (control; 0.5% BSA in DPBS containing 0.1% NaN_3) for 45 min at 4 °C, followed by three washes with 1 mL of DPBS for 1 min each. Nuclei were stained with Hoechst 34580 (1 µg/mL, 300 µL, 10 min at rt), followed by three additional PBS washes (1 mL, 1 min each). Finally, cells were maintained in FluoroBrite™ DMEM for imaging. Confocal images were acquired on a Nikon AX R inverted confocal microscope using 405 nm and 561 nm excitation lasers for the Hoechst and TAMRA channels, respectively, with Hoechst detected at 420-551 nm and TAMRA at 571-625 nm emission. Images were collected at 2048 x 2048 pixel resolution using a 60x oil-immersion objective in Galvano scan mode. The laser power and detector gain for the TAMRA channel were kept constant across all samples to allow direct visual and quantitative comparison of **N11b** and **N45** TAMRA fluorescence (561 nm excitation, laser intensity = 39.1, detector gain = 68.8, instrument units). Nuclei are shown in blue and TAMRA fluorescence in red. Scale bar = 50 µm.

Maleimide-thiol Michael addition reaction: N11b nanobody (1 mg/mL) was incubated with 1 mM TCEP for 30 min on ice to reduce the C-terminal cysteine. Excess TCEP was removed by buffer exchange using Amicon Ultra 0.5 mL centrifugal filter units, and the protein was exchanged into 0.1 M phosphate buffer at the desired pH (6.5, 7.5, or 8.5). The final N11b concentration was adjusted to 120 µM (1.8 mg/mL), and maleimide-PEG10 kDa (MAL–PEG–OH, Creative PEGWorks, Cat. No. PHB-946) was added to a final concentration of 1.2 mM, corresponding to a 1:10 molar ratio of reduced N11b to PEG reagent. Reactions were performed in a total volume of 20 µL and incubated overnight at room temperature. Product formation and conversion were evaluated by SDS-PAGE analysis.

PET/CT imaging experiments: For CFA-induced paw inflammation model, intraplantar injection of 50 µL of CFA was performed 24 hr prior to PET probe injection. For each animal study, 50-150 µg of nanobody was used. A ^{89}Zr solution (in 1.0 M oxalic acid) was neutralized by adjusting the pH to 6.8-7.5 using 2.0 M Na_2CO_3. Then, HEPES (50 mM, pH=7.4) was added. The pH-adjusted radionuclide mixture was combined with the nanobody solution and incubated for 2 h at rt on an agitating block (350 rpm). The radiolabeled nanobody was purified using a PD-10 size-exclusion

cartridge and eluted with PBS. Fractions showing clear radiolabeling were combined and adjusted to a final concentration of 100 µCi/100 µL with PBS. For in vivo studies, 100 µL (100 µCi) of the radiolabeled nanobody was administered intravenously via the lateral tail vein. Mice were anesthetized with 1.5% isoflurane in O_2 at ~1 L/min. The radiolabelling yield typically exceeded 70% (around 1 mCi) of the ^{89}Zr-N11b/^{89}Zr-N45-based probes. The specific activity of the nanobody PET probes were in the range of 10-20 µCi µg^{-1}. The weight, radioactivity, and injection time were recorded to ensure successful administration and for use in post-imaging analysis. At 24 h post-injection, mice were again anesthetized with 1.5% isoflurane in O_2 at ~1 L/min. Weight, radioactivity, and imaging time were recorded. PET imaging was performed using a G8 PET-CT small-animal scanner (PerkinElmer) with 10 min PET acquisitions followed by 2 min CT scans. In all studies, mice were euthanized by CO_2 inhalation after the final imaging session.

Image quantification and organ PET mean Standardized Uptake Value (SUV_{mean}) analysis: For the image quantification and biodistribution analysis, raw data were first processed using the manufacturer's automatic image reconstruction software, followed by detailed volumetric analysis using VivoQuant software (version 2022, Invicro) and Imalytics (Gremse-IT). The PET signal intensity was measured as activity concentration in µCi mm−3 and co-registered with CT scans to provide anatomical context. These overlaid images guided the manual generation of three-dimensional regions of interest (ROIs) for specific organs, allowing for precise organ-wise analysis. To standardize the uptake data across the cohort, the SUV_{mean} was calculated for each ROI by dividing the radioactivity concentration within the ROI volume by the radioactivity of the mouse and multiplying the mouse body weight at the time of scan acquisition: SUV_{mean} = concentration in ROI/(injected dose/mouse body weight).

RadioTLC: Nanobody PET probes (15 µg) were radiolabeled with ~150–200 µCi of ^{89}Zr for 1 h at room temperature. A 1 µL aliquot of the crude reaction mixture was diluted into 10 µL of 0.1 M phosphate buffer (pH 7.0) containing 10 mM EDTA, and 1 µL of this mixture was spotted onto a 120 mm iTLC-SG strip, with the sample applied 10 mm from the bottom and the solvent front developed to 10 mm from the top. The strip was dried and developed with 0.1 M phosphate buffer containing 10 mM EDTA. Radiochemical yield was calculated as (counts in product peak/total counts on strip)×100.

Procedures for the synthesis of compound 2

Synthetic scheme to produce compound 2:

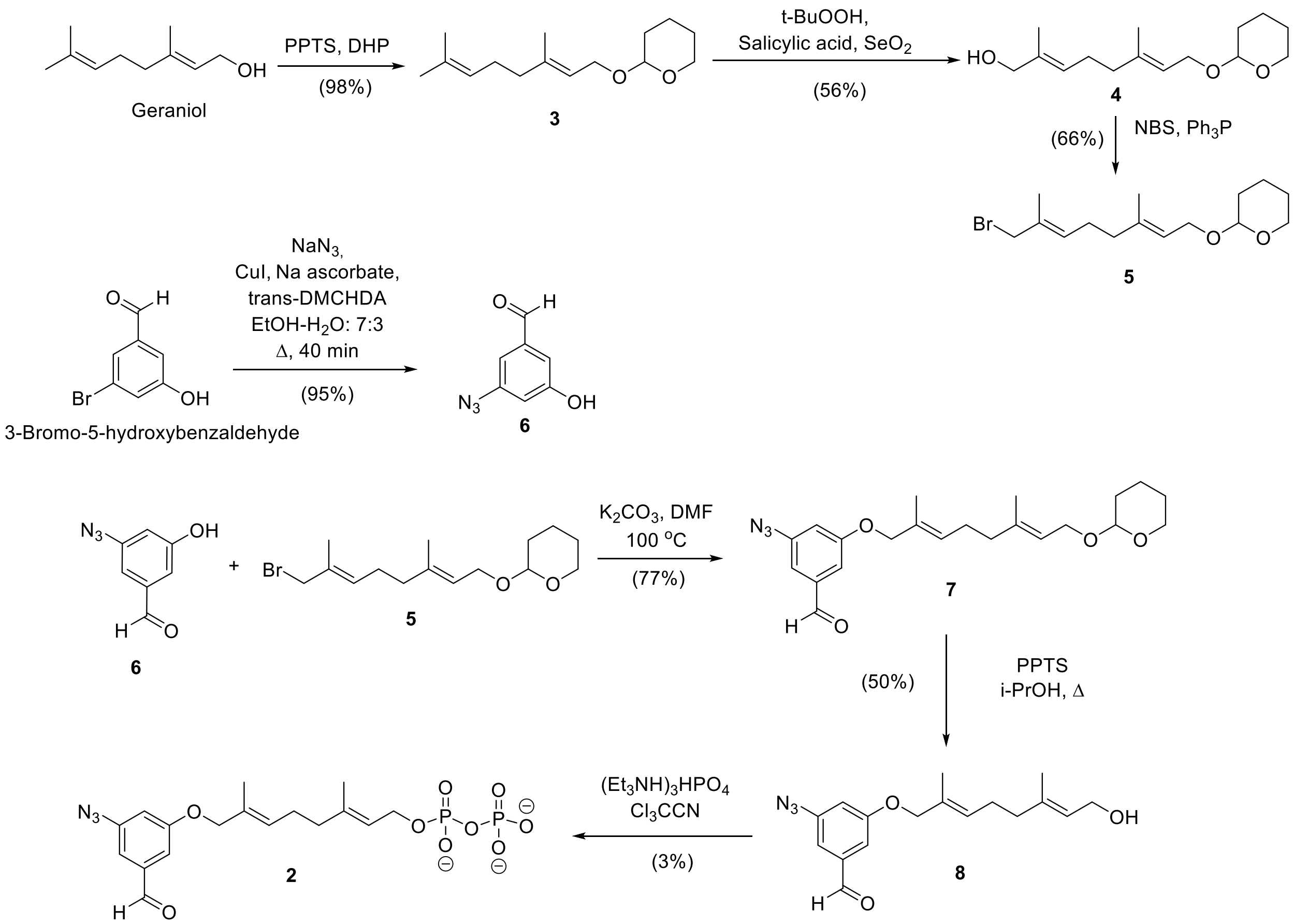

Scheme S1. Synthesis of Compound **2**.

Briefly, tetrahydropyranyl acetal (THP)-protected geraniol **3** was oxidized at C-8 to terminal alcohol **4**, then brominated using NBS and PPh_3 to yield bromide **5**. An azide-aldehyde-functionalized phenol **6** was synthesized in one step via Ullmann-type coupling of an aryl bromide using CuI/diamine catalysis with high yield (~99% conversion, ~95% yield). This phenol was then alkylated with the geranyl bromide derivative **5** using K_2CO_3. After THP deprotection, alcohol **8** was converted to the diphosphate **2** via direct phosphorylation using $(HNEt_3)_2HPO_4$ and CCl_3CN. Compound **2** was purified by reverse-phase HPLC and fully characterized by ^1H, ^{13}C and ^{31}P NMR and HR-ESI-MS and was obtained in 3% yield. While the diphosphorylation step is currently low yielding, related aromatic isoprenoid analogs of similar structure have been reported in much higher yield (upto 40%), suggesting that this step is amenable to further optimization.[2]

Compounds **3**, **4** and **5** were synthesized based on established protocols[3–5].

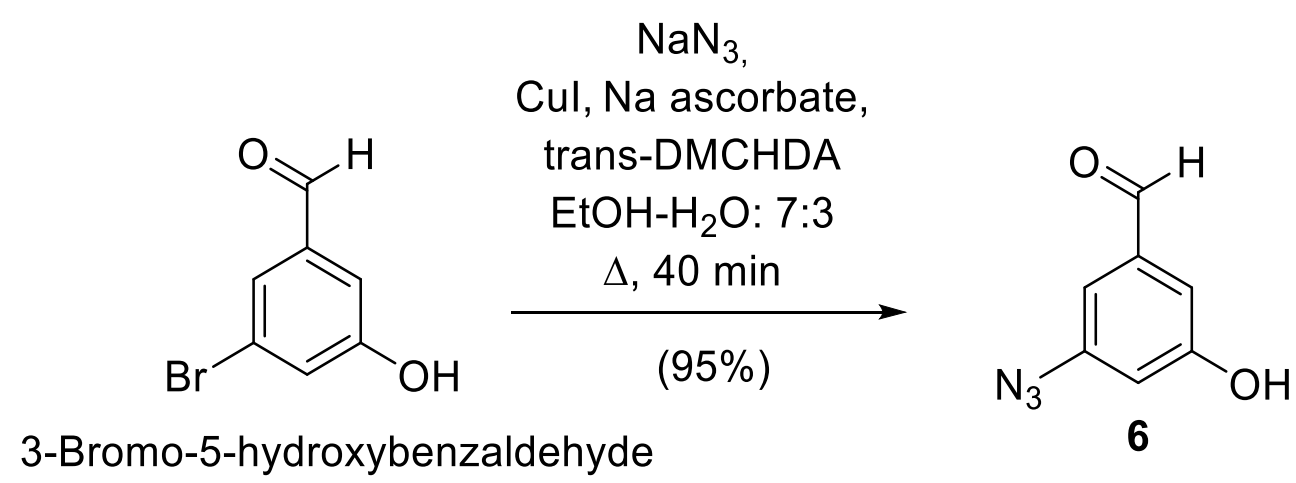

3-Azido-5-hydroxybenzaldehyde (6): This protocol was adopted from a previously published article[6] and patent[7]. The compound 3-bromo-5-hydroxybenzaldehyde (1 g, 4.97 mmol), NaN_3 (0.65 g, 9.95 mmol, 2 equiv), sodium ascorbate (0.05 g, 0.25 mmol, 0.05 equiv), CuI (0.09 g, 0.50 mmol, 0.10 equiv), trans-N, N'-dimethyl-1,2-cyclohexanediamine ligand (trans-DMCHDA) (0.11 g, 0.75 mmol, 0.15 equiv), and 25 mL EtOH/H_2O (7:3, v/v) were introduced into a two-necked round-bottom flask equipped with a magnetic stirring bar and a reflux condenser. After it was purged with Ar (g), the reaction mixture was heated to reflux with stirring and the reaction progress was monitored by TLC. When the aryl bromide was completely consumed, or when the reaction ceased to progress, the mixture was allowed to cool down to rt, and the EtOH was removed under vacuum via rotary evaporation. The crude product was extracted with 60 mL of EtOAc, and the organic layer washed with brine (3 x 60 mL), dried with $MgSO_4$ and concentrated in vacuo to yield a brown solid. The crude product was purified by flash chromatography (SiO_2, 2:5, EtOAc:hexanes, v/v), giving 0.75 g (95% yield) of the desired aryl azide **6** as a yellow powder. ^1H NMR (400 MHz, DMSO-d6) δ 10.32 (s, 1H), 9.89 (s, 1H), 7.12 - 7.03 (m, 2H), 6.76 (t, J = 2.2 Hz, 1H). ^{13}C NMR (100 MHz, DMSO-d6): 192.47, 159.27, 141.64, 138.69, 112.39, 111.57, 110.84. HRMS (ESI), m/z for $[M-H]^-$: Calcd for $[C_7H_4N_3O_2]$; 162.0303, Obs 162.0312.

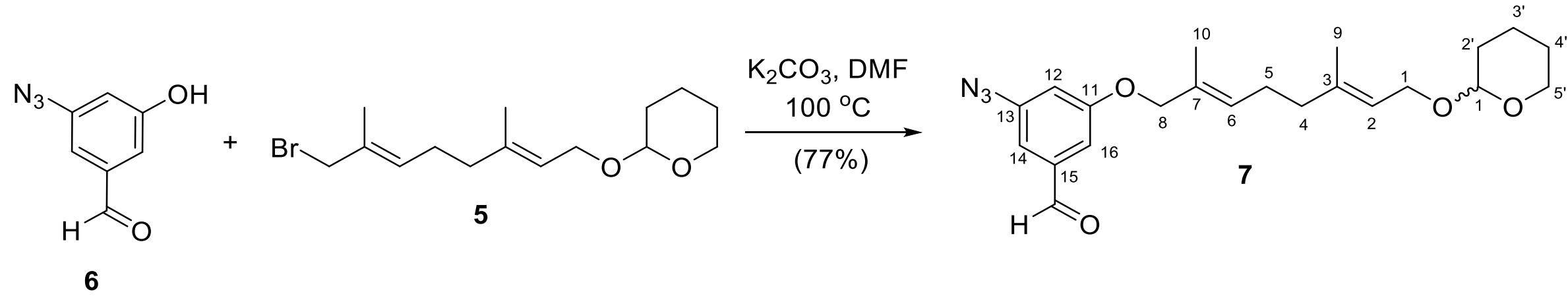

3-Azido-5-((2E,6E)-2,6-dimethyl-8-((tetrahydro-2H-pyran-2-yl)oxy)octa-2,6-dien-1 yl)oxy)benzaldehyde (7): A solution of bromide **5** (0.36 g, 1.14 mmol) and compound **6** (0.29 g, 2.00 mmol, 1.75 equiv) in anhydrous DMF (15 mL) was prepared. Solid K_2CO_3 (0.50 g, 3.62 mmol, 3.20 equiv) was added, and the reaction mixture was stirred at 100 °C for 3 h. Reaction progress was monitored by TLC (hexanes/EtOAc, 2:1 v/v). Upon completion, the mixture was cooled to rt and extracted with EtOAc (60 mL). The organic phase was washed with H_2O (3 x 150 mL), followed with brine (3 x 60 mL) and dried over anhydrous $MgSO_4$. The dried organic phase was concentrated under reduced pressure to afford a crude yellow oil. The crude product was purified by flash chromatography (SiO_2; gradient elution from 1:0 to 5:1 hexanes/Et_2O, v/v) yielding the desired ether product **7** as a yellow oil (0.32 g, 77% yield, mixture of C6=C7 E/Z isomers, ~2.4 : 1 ratio).

Compound **7** exists as a mixture of E and Z isomers due to isomerization at the C6=C7 double bond. Few proton NMR signals that are uniquely attributable to the Z isomer are listed separately.

^{1}H NMR (400 MHz, $CDCl_3$) for E-C6=C7 isomer: δ 9.91 (s, 1H), 7.21 - 7.17 (m, 1H), 7.16 - 7.13 (m, 1H), 6.84 - 6.80 (m, 1H), 5.60 - 5.53 (m, 1H), 5.42 - 5.35 (m, 1H), 4.65 - 4.62 (m, 1H), 4.47 - 4.42 (m, 2H), 4.29 - 4.22 (m, 2H), 4.08 - 3.99 (m, 1H), 3.94 - 3.87 (m, 1H), 3.56 - 3.50 (m, 1H), 2.29 - 2.17 (m, 2H), 2.15 - 2.08 (m, 2H), 1.75 (s, 3H), 1.70 (s, 3H), 1.66 - 1.46 (m, 5H).

^{1}H NMR (400 MHz, CDCl3) unique peaks for Z-C6=C7 isomer: δ 9.90 (s, 1H), 5.51 - 5.45 (m, 1H).

^{13}C NMR (100 MHz, $CDCl_3$) δ 191.18, 160.66, 142.55, 139.56, 138.76, 132.26, 130.43, 129.49, 127.57, 121.22, 112.00, 98.03, 74.64, 72.99, 63.75, 62.27, 39.31, 30.82, 25.61, 19.73, 16.52, 14.16. HRMS (ESI), m/z for $[M+Na]^+$: Calcd for $[C_{22}H_{29}N_3O_4Na]$; 422.2056, Obs 422.2044.

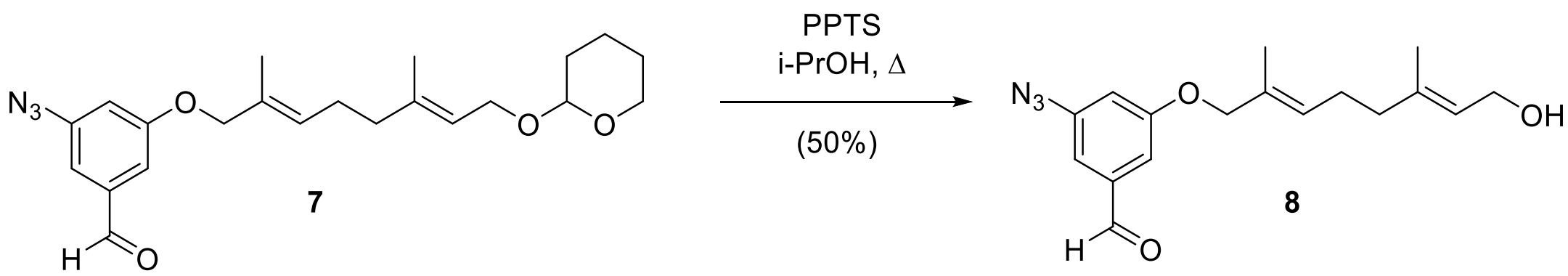

3-Azido-5-(((2E,6E)-8-hydroxy-2,6-dimethylocta-2,6-dien-1-yl)oxy)benzaldehyde (8): Compound **7** (1.30 g, 4.05 mmol) was dissolved in 20 mL of isopropyl alcohol in a 50-mL round-bottom flask. PPTS (30 mg, 0.26 mmol, 0.06 equiv) was added as a catalyst. The reaction mixture was refluxed for 4 h at 85 °C (hot plate set to 130 °C) in an oil bath until TLC analysis (hexanes/EtOAc, 2:1 v/v) indicated almost complete conversion to the product. Saturated $NaHCO_3$ (5 mL) was then added, followed by 50 mL of EtOAc. The organic layer was separated, washed with 10 mL of brine, and dried over Na_2SO_4. After filtration and evaporation of the solvent under reduced pressure, the crude product was purified by flash chromatography (SiO_2; gradient elution 1:0 to 3:2 hexanes/Et_2O, v/v) to afford 0.5 g of compound **12** (50% yield) as a pale-yellow oil. ^{1}H NMR (400 MHz, $CDCl_3$) δ 9.91 (s, 1H), 7.18 (dd, J = 2.4, 1.2 Hz, 1H), 7.13 (dd, J = 2.1, 1.2 Hz, 1H), 6.81 (t, J = 2.2 Hz, 1H), 5.57-5.48 (m, 1H), 5.43-5.37 (m, 1H), 4.45 (s, 2H), 4.15 (d, J = 6.8 Hz, 2H), 2.26 - 2.16 (m, 3H), 2.11-2.05 (m, 2H), 1.73 (s, 3H), 1.68 (s, 3H). ^{13}C NMR (101 MHz, $CDCl_3$) δ 191.25, 160.73, 142.60, 139.06, 138.80, 130.60, 129.40, 124.05, 112.89, 112.11, 111.52, 74.66, 59.52, 38.95, 26.05, 16.39, 14.01. HRMS (ESI), m/z for $[M+Na]^+$: Calcd for $[C_{17}H_{21}N_3O_3Na]$; 338.1480, Obs 338.1470.

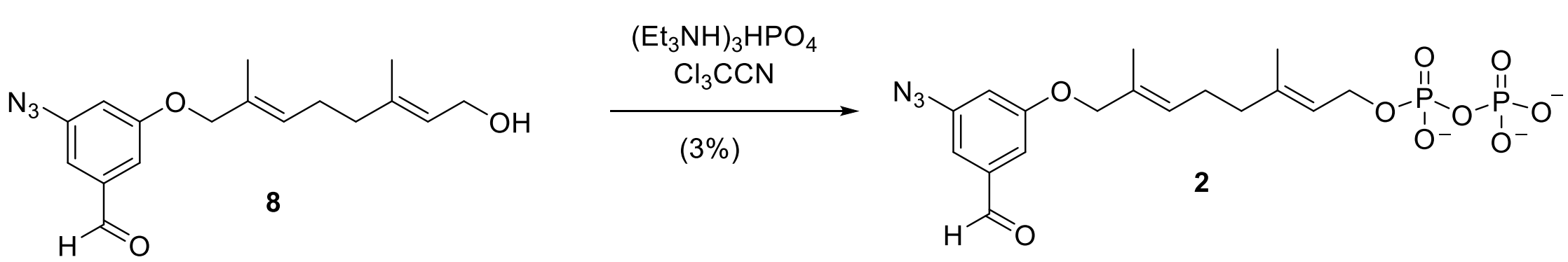

(2E,6E)-8-(3-Azido-5-formylphenoxy)-3,7-dimethylocta-2,6-dien-1-yl diphosphate: Compound **8** (680 mg, 2.16 mmol) was dissolved in CCl_3CN (1.3 mL, 12.94 mmol, 6 equiv) in a 100-mL round-bottom flask. In a separate 50-mL flask, $(Et_3NH)_2HPO_4$ (1.94 g, 6.47 mmol, 3 equiv) was dissolved in 25 mL of CH_3CN by placing the flask in a 30 °C water bath. Using an addition funnel, the resulting solution was added dropwise over 3 h to the mixture of Compound **8** and CCl_3CN while stirring at rt. Slow addition was critical for obtaining the highest possible yield. (Note: To prepare the phosphorylating agent, 32.2 mL of 1% v/v H_3PO_4 was mixed with 1.8 mL of triethylamine and lyophilized to yield 1.94 g of $(Et_3NH)_2HPO_4$.) Following the addition, the mixture

was stirred for an additional 50 min at rt, and the solvent was removed under reduced pressure using a rotary evaporator. The crude material was subjected to purification on an Amberchrome ion-exchange column (Millipore Sigma, Cat: 428604, 200-400 mesh, 10 g resin manually packed in 16 mm ID glass column). The resin was first treated with 30% aqueous NH_4OH (200 mL), followed by equilibration with 25 mM aqueous NH_4HCO_3 containing 2% isopropanol (v/v) (solvent A, 250 mL). The crude product was dissolved in a minimal volume of solvent A, loaded onto the column, and eluted as a single fraction using solvent A (200 mL). Formation of the desired diphosphate compound was confirmed by negative-mode ESI-MS. The eluent was concentrated under reduced pressure and lyophilized to afford a crude dark brown solid. The crude product was redissolved in 10 mL of 25 mM aqueous NH_4HCO_3, filtered through a 0.45-μm syringe filter, and purified by semi-preparative reversed-phase HPLC on an Agilent 1100 system using a ZORBAX 300SB-C18 column (5 μm, 9.4 × 250 mm). Separation was achieved using a binary gradient of solvent A (25 mM aqueous NH_4HCO_3) and solvent B (CH_3CN) at 5.0 mL/min, with detection at 214 and 280 nm. The gradient increased from 0% to 35% solvent B over 30 min, then to 100% solvent B over 5 min. The reaction produced a mixture of mono-, di-, and triphosphate species: the triphosphate eluted first, followed by the diphosphate (Compound **2**) between 20-25% solvent B, and then the monophosphate. Fractions were collected every 2 min once peaks appeared in this range. Some fractions contained mixtures of adjacent species, necessitating a second purification step for complete separation. Collected fractions were analyzed by negative-mode ESI-MS, which confirmed the desired mass: calcd for $C_{17}H_{22}N_3O_9P_2^-$ $[M-H]^-$ 474.0831, found 474.0831. All product-containing fractions were combined, transferred to a lyophilization jar, frozen in liquid nitrogen, and lyophilized for 24 h to yield ~30 mg (3% yield) of compound **2** as a white powder.

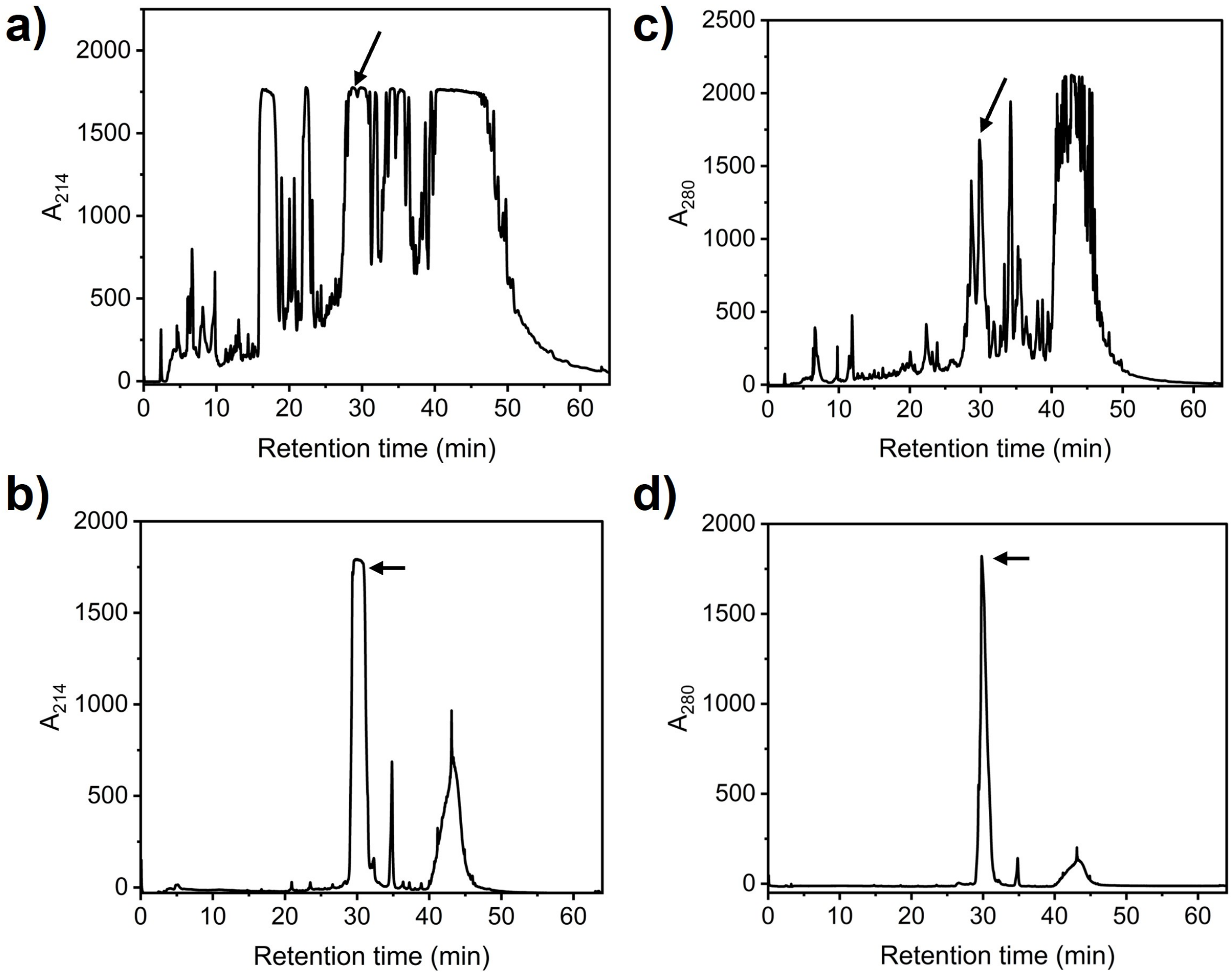

HPLC chromatograms for purification of compound **2**. a) Crude sample (214 nm); b) Pooled fractions containing the desired product after second purification (214 nm); c) Crude sample (280 nm); d) Pooled fractions containing the desired product after second purification (280 nm). The black arrow marks the peak corresponding to compound **2**.

The purified diphosphate compound was dissolved in aqueous 25 mM NH_4HCO_3 for NMR analysis and any subsequent experiments. The concentration of the diphosphate compound solutions was measured using ^{31}P NMR with an internal standard made with Na_2HPO_4 in D_2O. The ^{31}P NMR sample was prepared by mixing 100 µL of diphosphate solution with 100 µL of Na_2HPO_4 standard solution, 50 µL of ammonium-d*4* deuteroxide solution, and 500 µL of D_2O .

^{1}H NMR (400 MHz, D_2O) δ 9.72 (s, 1H), 7.15 - 7.13 (m, 1H), 7.12 - 7.10 (m, 1H), 6.81 (t, J = 2.2 Hz, 1H), 5.56 - 5.49 (m, 1H), 5.38 - 5.31 (m, 1H), 4.44 (s, 2H), 4.36 (t, J = 6.6 Hz, 2H), 2.2 - 2.09 (m, 2H), 2.06 - 1.97 (m, 2H), 1.62 (s, 6H). ^{13}C NMR (101 MHz, D_2O) δ 194.85, 160.35, 159.55, 142.25, 137.96, 130.51, 130.00, 120.10, 120.02, 113.03, 112.51, 74.56, 62.34, 38.23, 25.45, 15.61, 13.00. ^{31}P NMR (162 MHz, D_2O) δ -6.82 (d, J = 22.9 Hz), -10.53 (d, J = 22.4 Hz). ^{31}P NMR (162 MHz, D2O) δ -6.82 (d, J = 22.9 Hz), -10.53 (d, J = 22.4 Hz). HRMS (ESI), m/z for $[M-H]^-$: Calcd for $[C_{17}H_{22}N_3O_9P_2]$; 474.0831, Obs 474.0831.

Spectral data for synthetic compounds

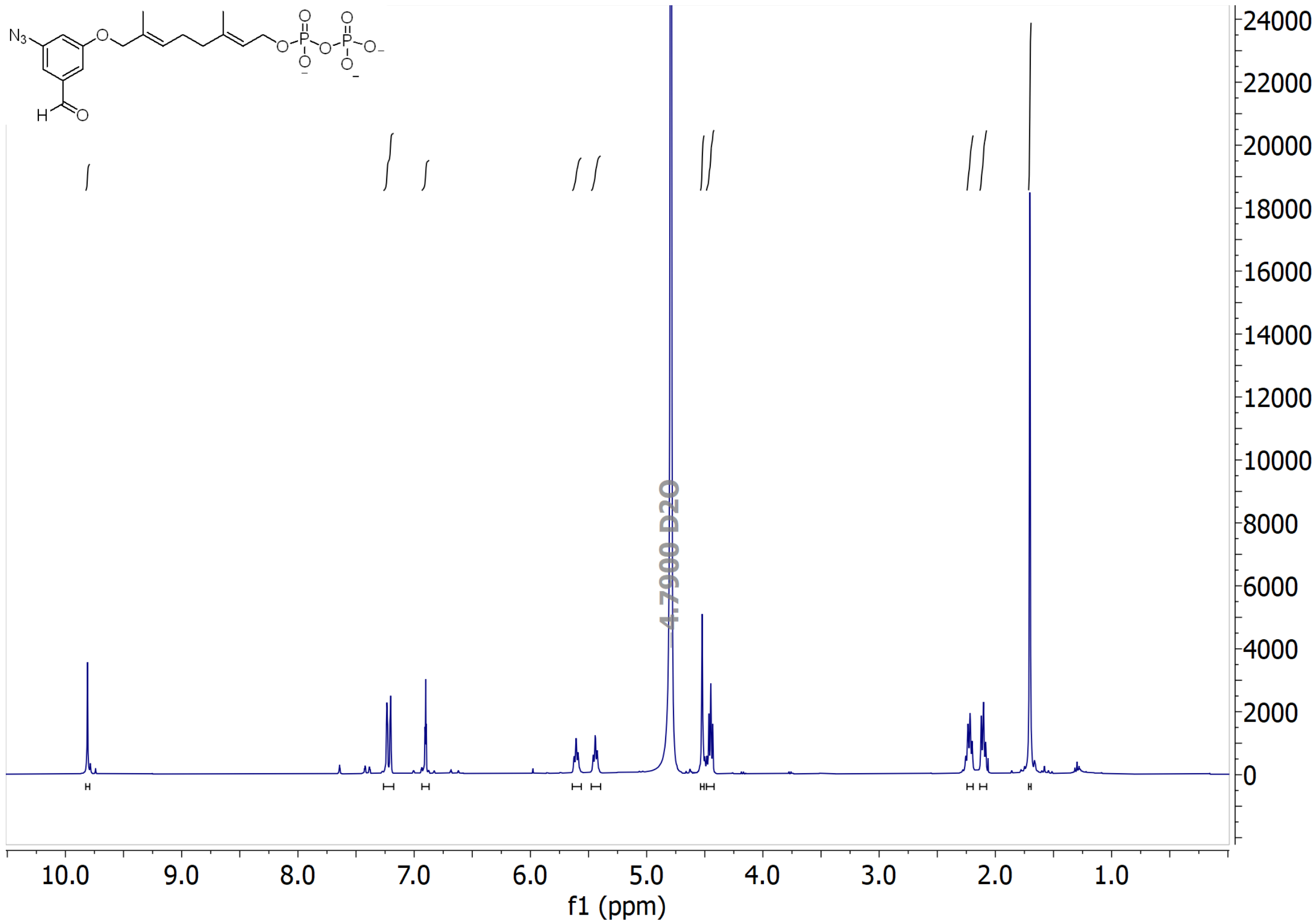

Compound **2** ^{1}H NMR

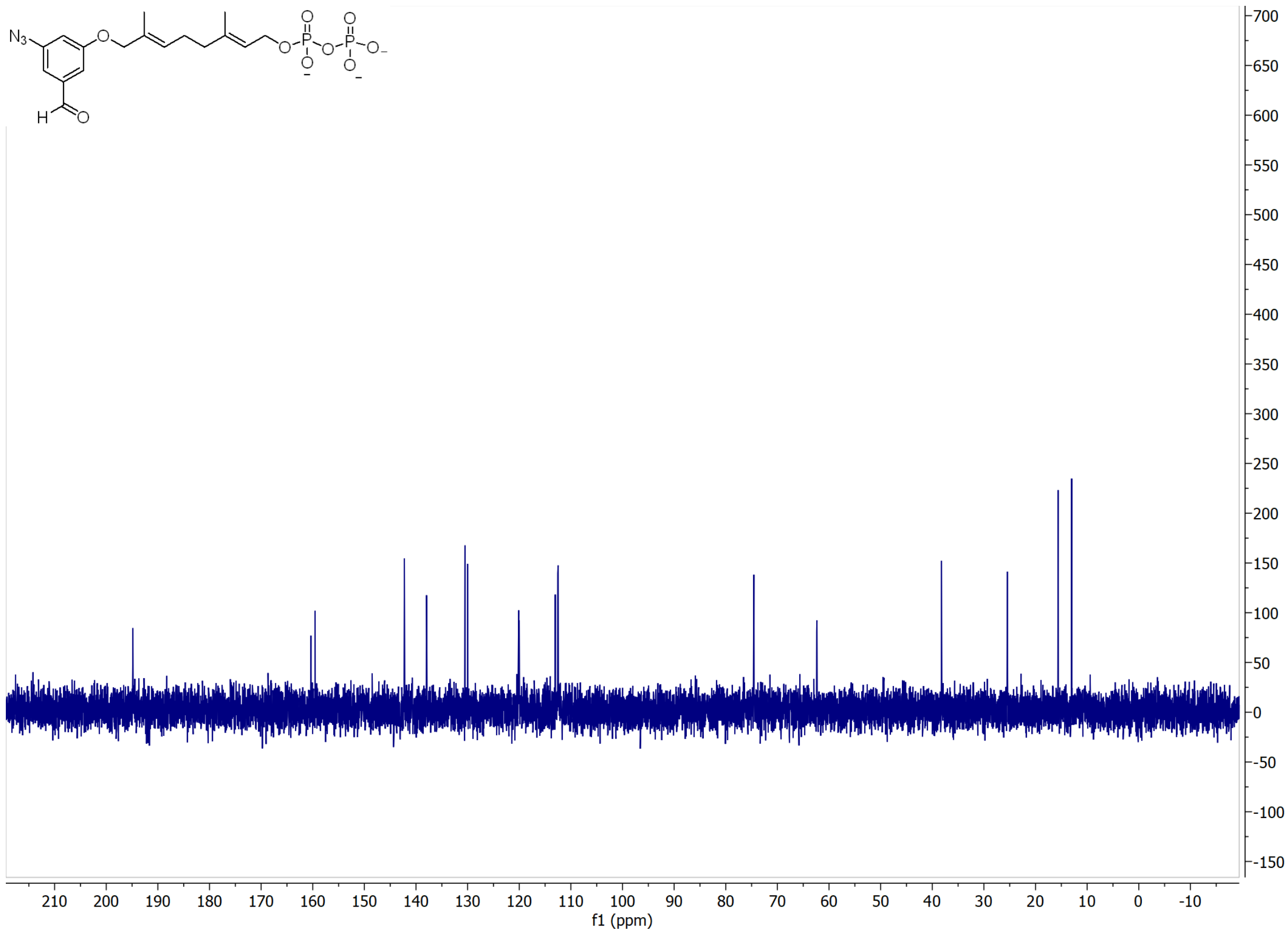

Compound **2** ^{13}C NMR

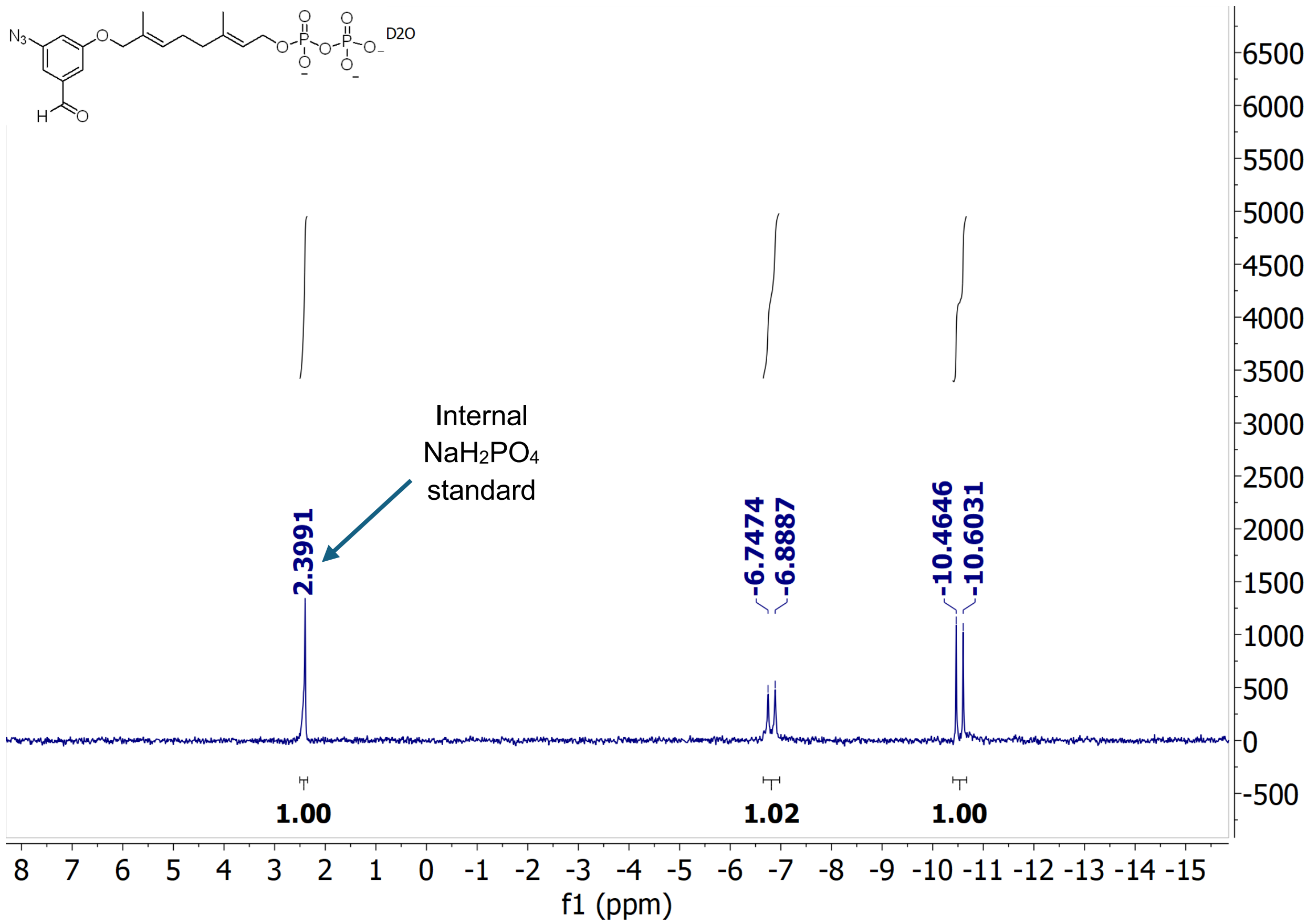

Compound **2** ^{31}P NMR

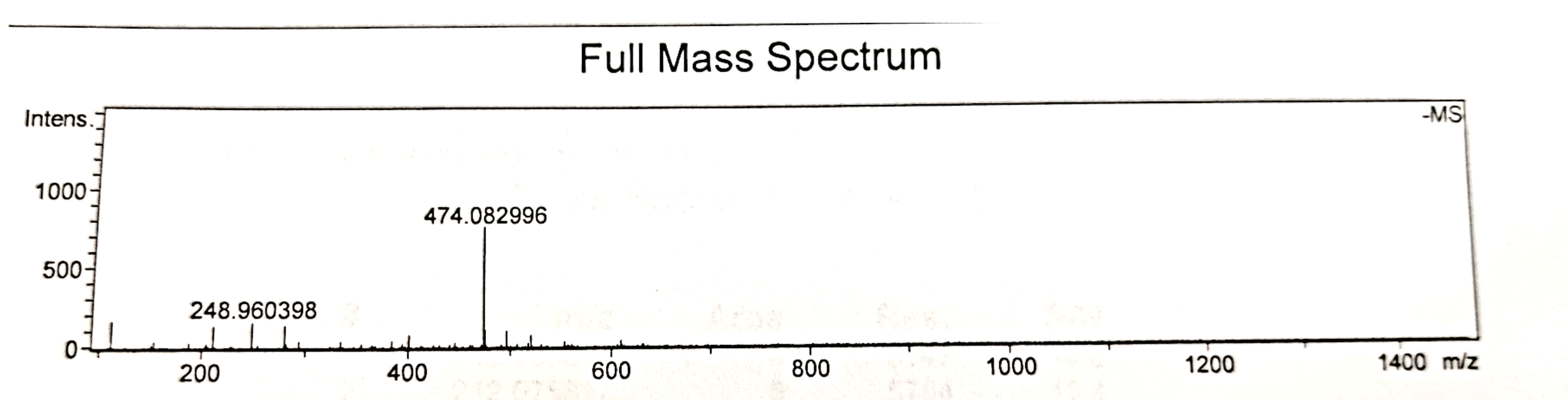

HRMS Compound **2**

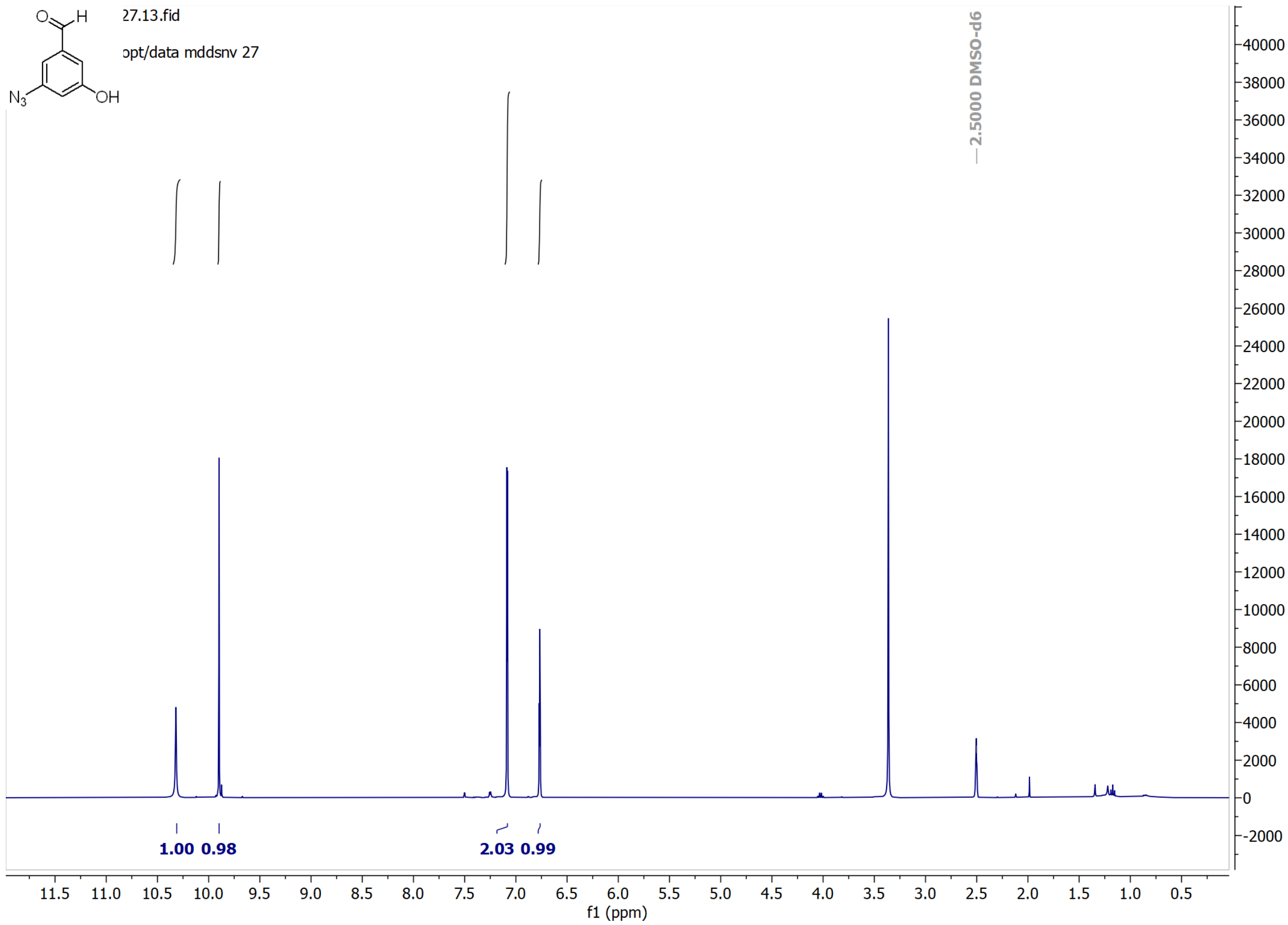

Compound **6** ^{1}H NMR

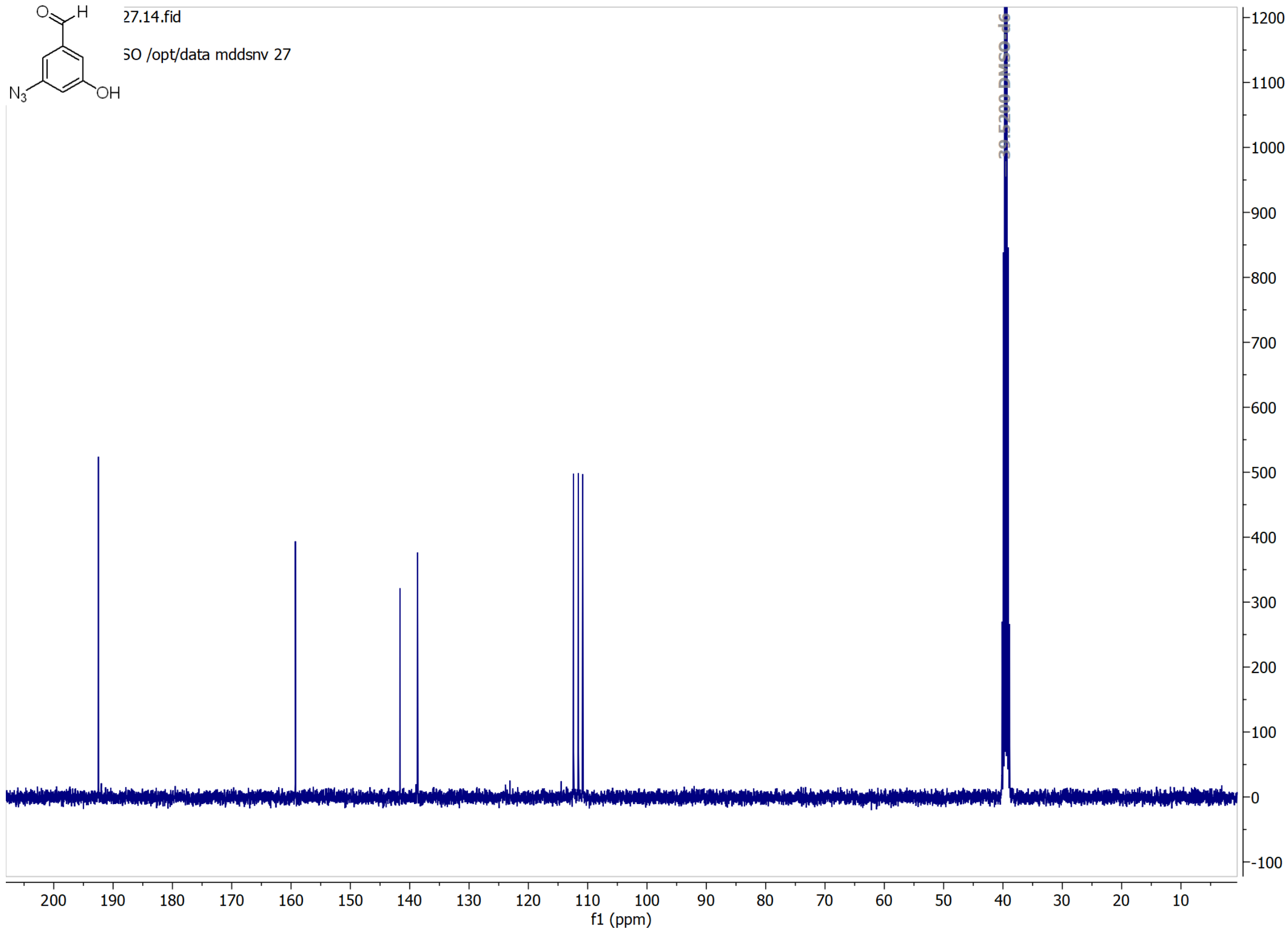

Compound **6** ^{13}C NMR

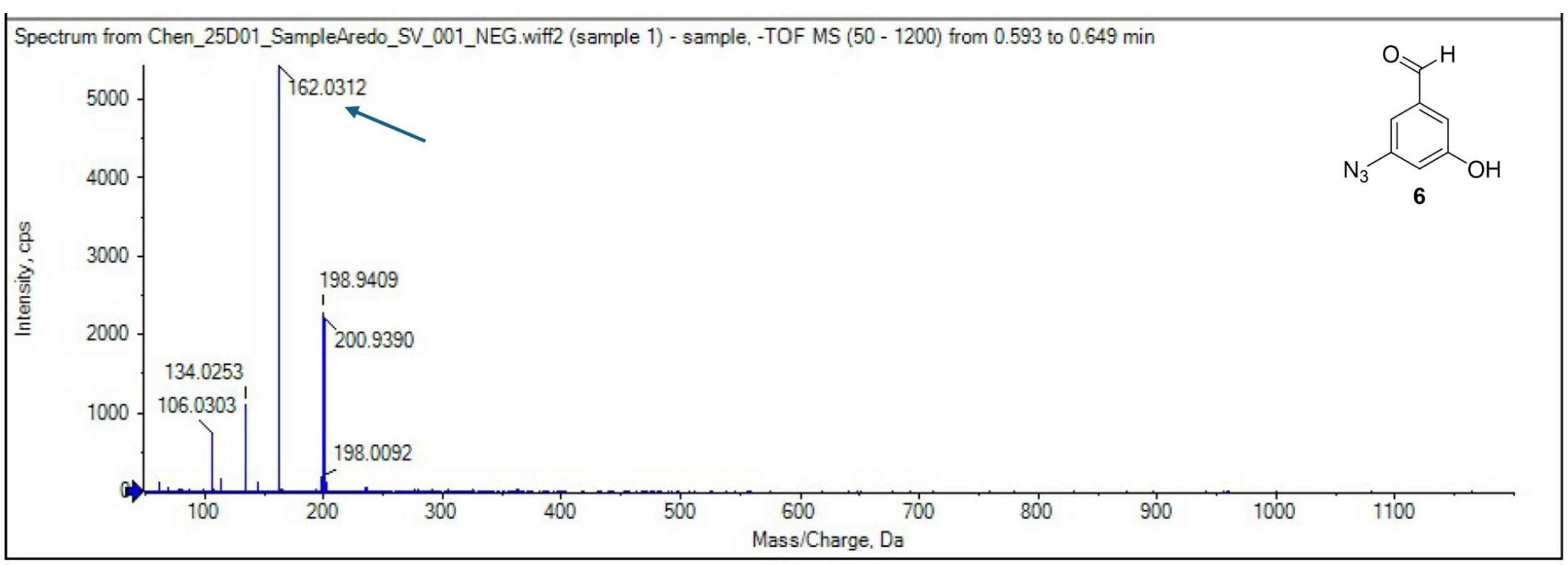

Compound **6** HRMS

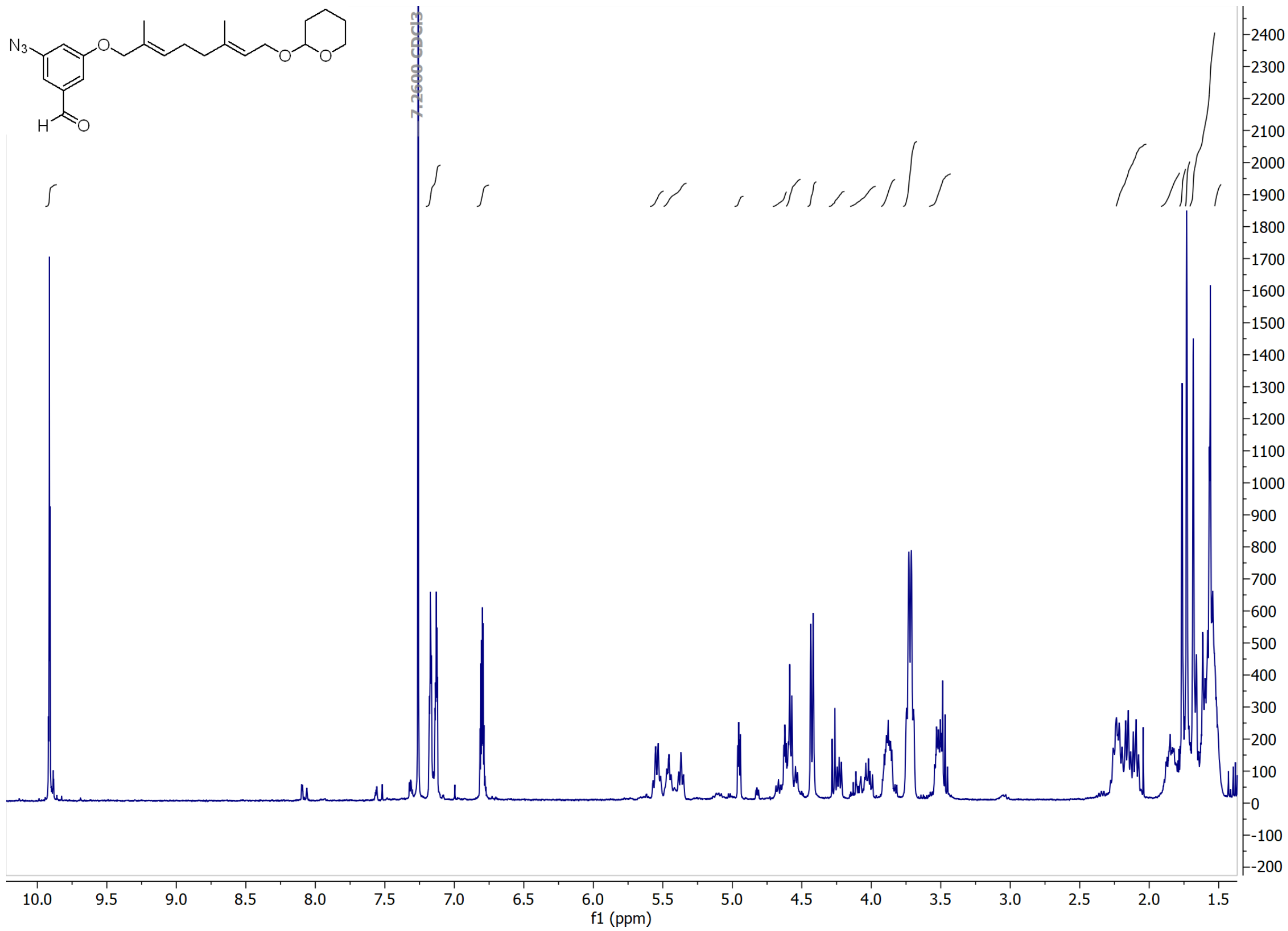

Compound **7** ^{1}H NMR

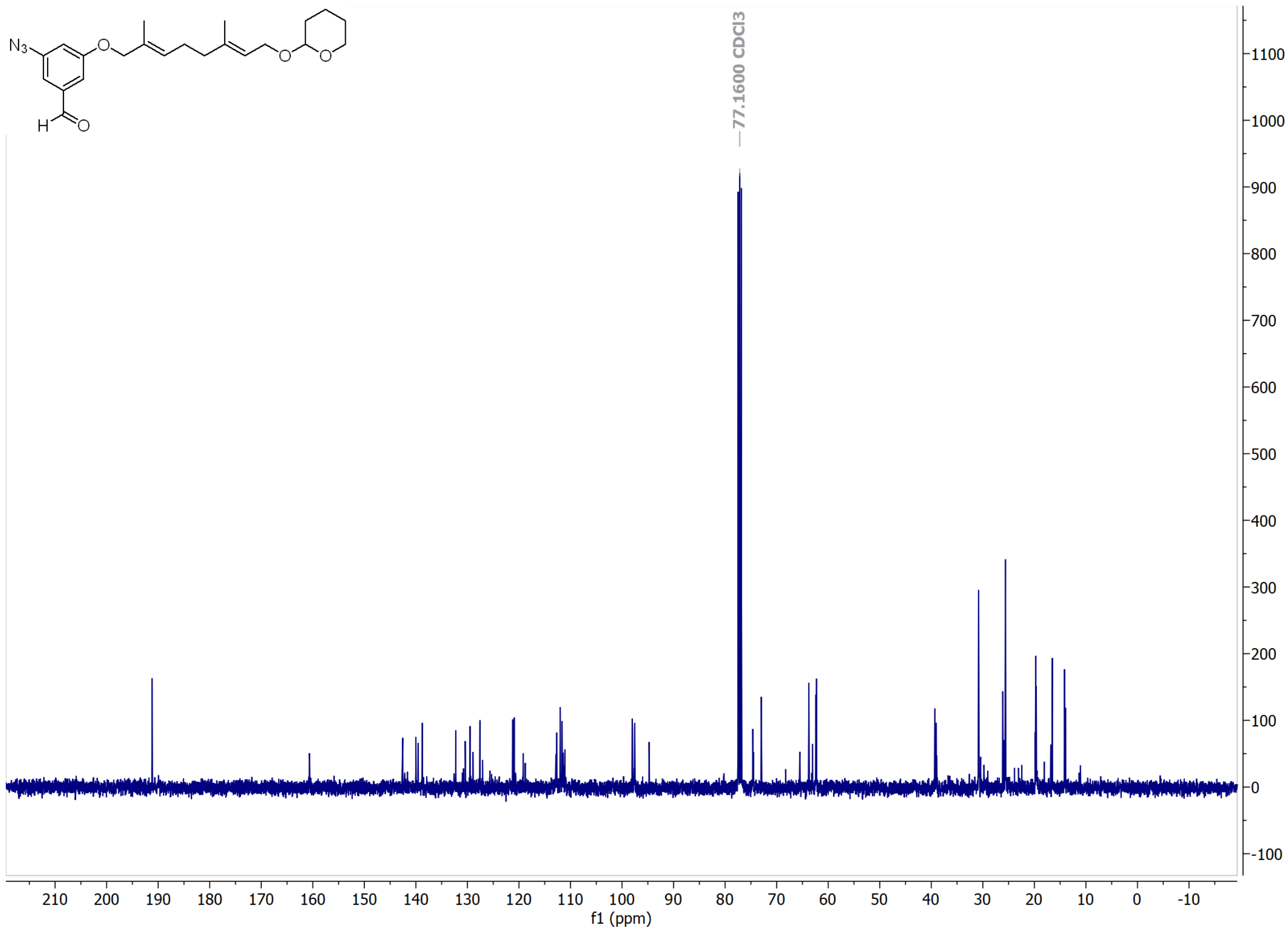

Compound **7** ^{13}C NMR

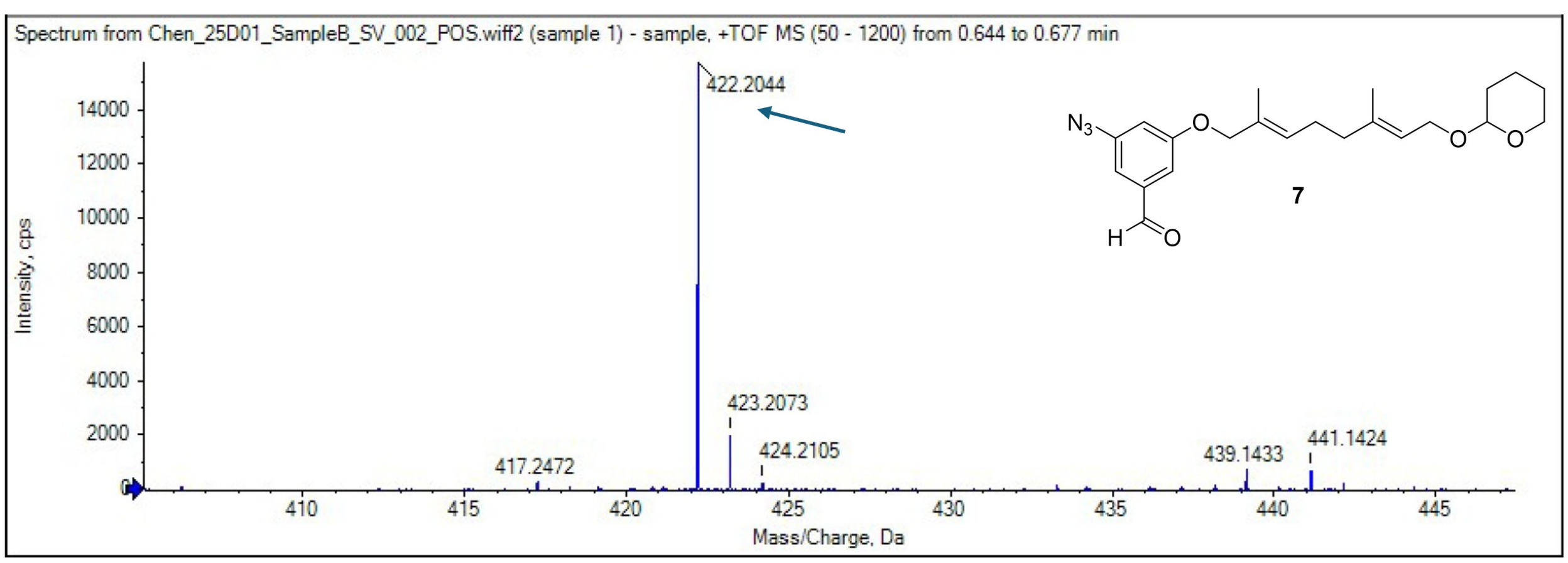

Compound **7** HRMS

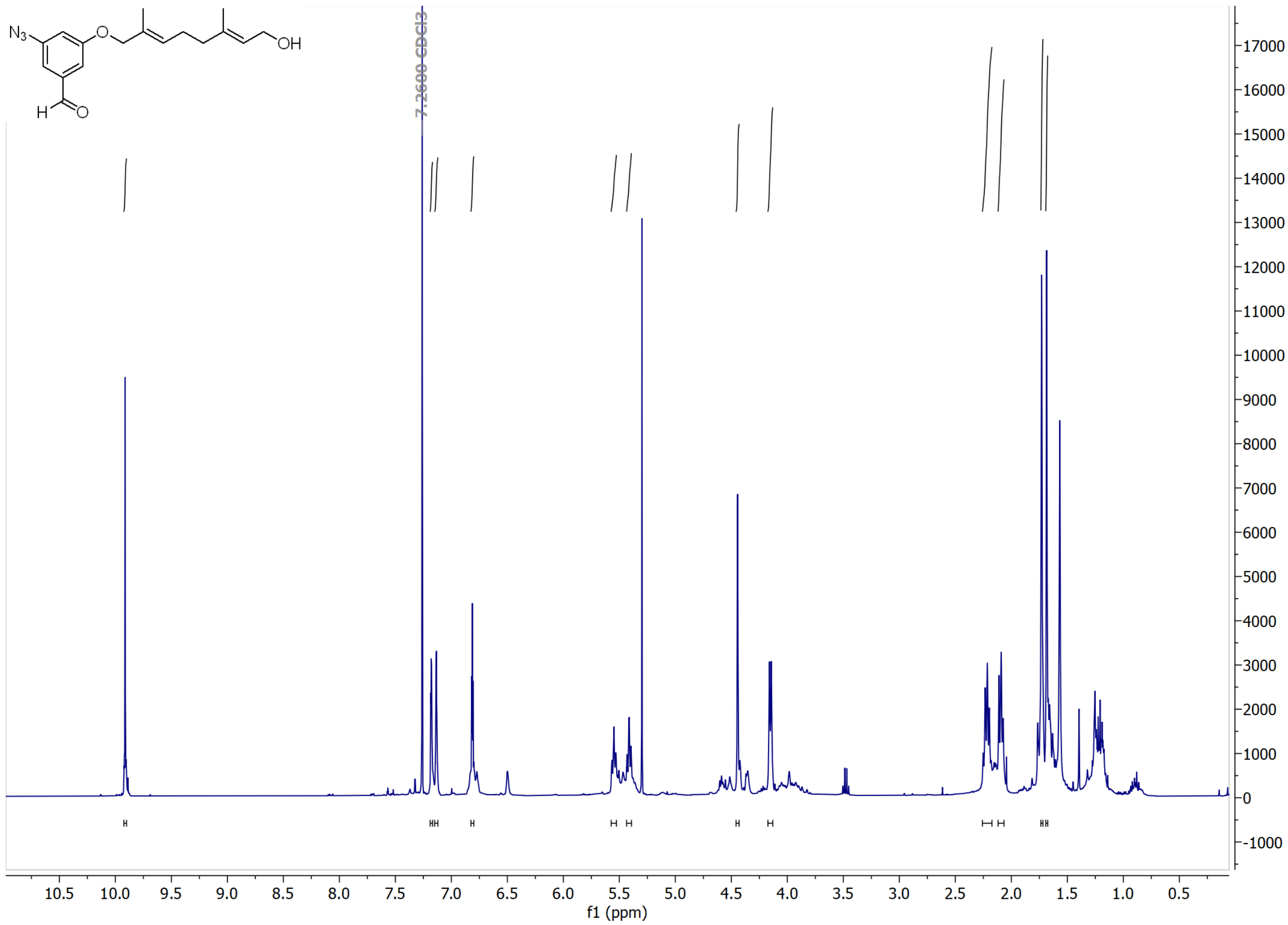

Compound **8** ^{1}H NMR

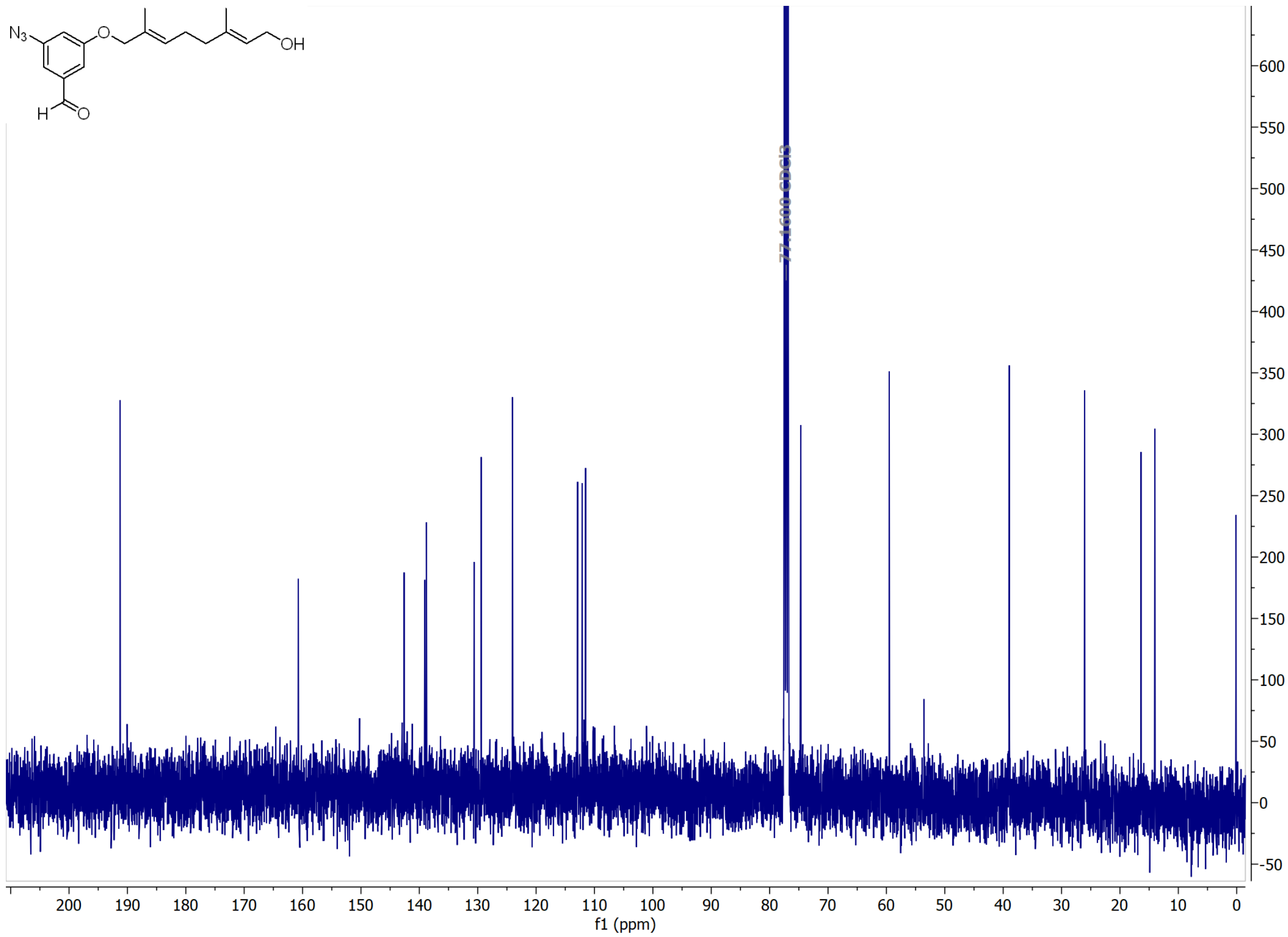

Compound **8** ^{13}C NMR

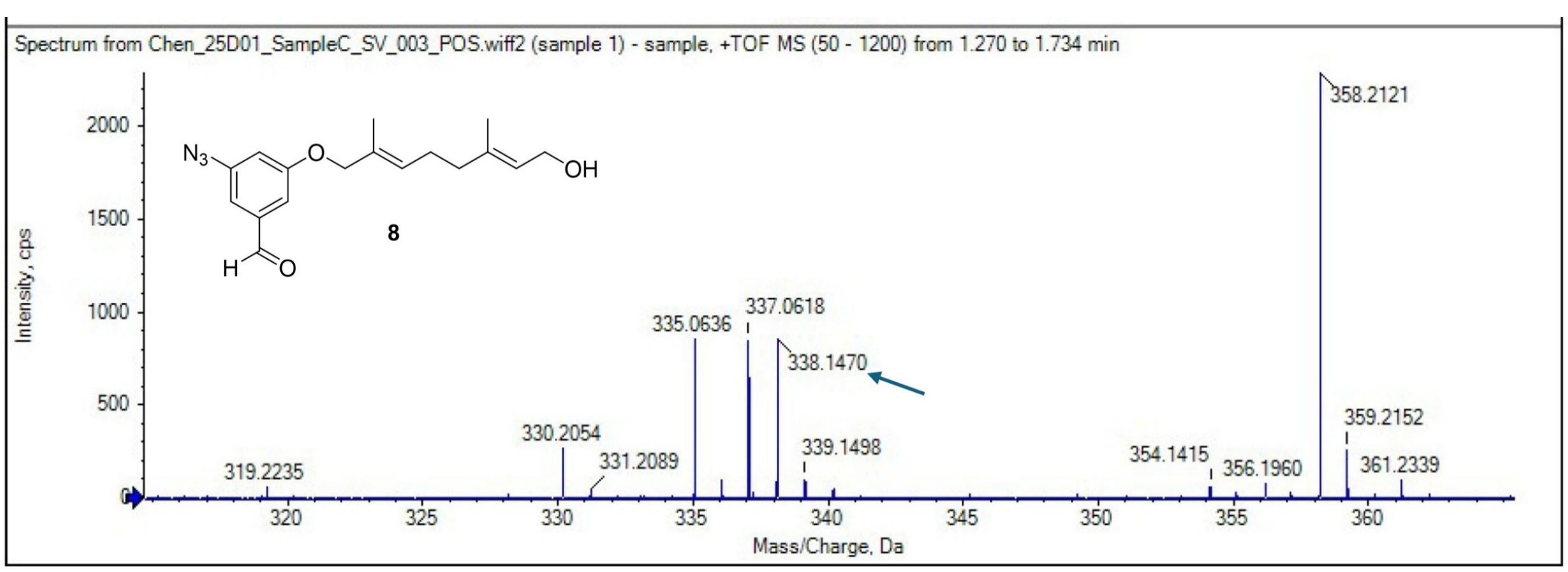

Compound **8** HRMS

References

[1] M. W. Rose, N. D. Rose, J. Boggs, S. Lenevich, J. Xu, G. Barany, M. D. Distefano, "Evaluation of geranylazide and farnesylazide diphosphate for incorporation of prenylazides into a CAAX box-containing peptide using protein farnesyltransferase*" *J. Pept. Res.* **2005**, *65*, 529–537.

[2] V. Kumar, B. P. Johnson, P. S. Mandal, D. R. Sheffield, D. A. Dimas, R. Das, S. Maity, M. D. Distefano, S. Singh, "The utility of Streptococcus mutans undecaprenol kinase for the chemoenzymatic synthesis of diverse non-natural isoprenoids" *Bioorganic Chem.* **2024**, *151*, 107707.

[3] S. A. Auger, S. Venkatachalapathy, K. F. G. Suazo, Y. Wang, A. W. Sarkis, K. Bernhagen, K. Justyna, J. V. Schaefer, J. W. Wollack, A. Plückthun, L. Li, M. D. Distefano, "Broadening the Utility of Farnesyltransferase-Catalyzed Protein Labeling Using Norbornene–Tetrazine Click Chemistry" *Bioconjug. Chem.* **2024**, *35*, 922–933.

[4] M. M. Mahmoodi, M. Rashidian, Y. Zhang, M. D. Distefano, "Application of *meta*- and *para*- Phenylenediamine as Enhanced Oxime Ligation Catalysts for Protein Labeling, PEGylation, Immobilization, and Release" *Curr. Protoc. Protein Sci.* **2015**, *79*, DOI 10.1002/0471140864.ps1504s79.

[5] M. Rashidian, S. C. Kumarapperuma, K. Gabrielse, A. Fegan, C. R. Wagner, M. D. Distefano, "Simultaneous Dual Protein Labeling Using a Triorthogonal Reagent" *J. Am. Chem. Soc.* **2013**, *135*, 16388–16396.

[6] J. Andersen, U. Madsen, F. Björkling, X. Liang, "Rapid Synthesis of Aryl Azides from Aryl Halides under Mild Conditions" *Synlett* **2005**, 2209–2213.

[7] C. Fetzer, V. Korotkov, S. Sieber, *Clpx Inhibitory Compounds for the Treatment of Multi Resistant Staphylococcus Aureus Virulence and for the Treatment of Leukemia*, **2018**.
